# Factors Influencing Fidelity to a Calorie Posting Policy in Public Hospitals: A Mixed Methods Study

**DOI:** 10.3389/fpubh.2021.707668

**Published:** 2021-08-13

**Authors:** Claire Kerins, Colette Kelly, Caitlin M. Reardon, Catherine Houghton, Elaine Toomey, Catherine B. Hayes, Fiona Geaney, Ivan J. Perry, Jenny McSharry, Sheena McHugh

**Affiliations:** ^1^Discipline of Health Promotion, School of Health Sciences, National University of Ireland Galway, Galway, Ireland; ^2^VA Center for Clinical Management Research, VA Ann Arbor Healthcare System, Ann Arbor, MI, United States; ^3^School of Nursing and Midwifery, National University of Ireland Galway, Galway, Ireland; ^4^Faculty of Education and Health Sciences, School of Allied Health, University of Limerick, Limerick, Ireland; ^5^Health Research Institute, University of Limerick, Limerick, Ireland; ^6^Public Health and Primary Care, Institute of Population Health, School of Medicine, Trinity College Dublin, Dublin, Ireland; ^7^School of Public Health, University College Cork, Cork, Ireland; ^8^Health Behaviour Change Research Group, School of Psychology, National University of Ireland Galway, Galway, Ireland

**Keywords:** menu labelling, implementation science, implementation fidelity, Consolidated Framework for Implementation Research, mixed methods, multiple case study, triangulation

## Abstract

**Background:** Labelling menus with nutrition information has increasingly become an important obesity policy option. While much research to-date has focused on determining its effectiveness, few studies report the extent to which menu labelling is implemented as designed. The aim of this study was to explore factors influencing fidelity to a calorie posting policy in Irish acute public hospitals.

**Methods:** A mixed methods sequential explanatory study design was employed, with a nested case study for the qualitative component. Quantitative data on implementation fidelity at hospitals were analysed first and informed case sampling in the follow-on qualitative phase. Maximum variation sampling was used to select four hospitals with high and low levels of implementation and variation in terms of geographic location, hospital size, complexity of care provided and hospital type. Data were collected using structured observations, unstructured non-participant observations and in-depth semi-structured interviews. The Consolidated Framework for Implementation Research guided qualitative data collection and analysis. Using framework analysis, factors influencing implementation were identified. A triangulation protocol was used to integrate fidelity findings from multiple sources. Data on influencing factors and fidelity were then combined using joint displays for within and cross-case analysis.

**Results:** Quantitative fidelity data showed seven hospitals were categorised as low implementers and 28 hospitals were high implementers of the policy. Across the four hospitals selected as cases, qualitative analysis revealed factors influencing implementation and fidelity were multiple, and operated independently and in combination. Factors were related to the internal hospital environment (e.g., leadership support, access to knowledge and information, perceived importance of calorie posting implementation), external hospital environment (e.g., national policy, monitoring), features of the calorie posting policy (e.g., availability of supporting materials), and the implementation process (e.g., engaging relevant stakeholders). Integrated analysis of fidelity indicated a pattern of partial adherence to the calorie posting policy across the four hospitals. Across all hospitals, there was a consistent pattern of low adherence to calorie posting across all menu items on sale, low adherence to calorie information displayed per standard portion or per meal, low adherence to standardised recipes/portions, and inaccurate calorie information.

**Conclusion:** Efforts to maximise fidelity require multi-level, multi-component strategies in order to reduce or mitigate barriers and to leverage facilitators. Future research should examine the relative importance of calorie posting determinants and the association between implementation strategies and shifts in fidelity to intervention core components.

## Background

The World Health Organisation estimates that, worldwide, the prevalence of obesity has reached epidemic proportions (1). While there are multifactorial drivers (2), research shows eating outside the home may play a role in the current obesity epidemic (3–6). Eating outside the home has become more frequent (7–9), and is associated with higher energy content of meals (10–13) and consumer underestimation of calorie content (14, 15). In an effort to address this, labelling menus with nutrition information (such as the calorie content of menu items) at the point of sale has become a popular policy option (16–18). A number of countries and regions around the world have menu labelling on a voluntary or mandatory basis for food service businesses (19–23). The process for menu labelling policy development across food businesses is not routinely documented. Furthermore, a number of workplace and healthcare organisations have introduced menu labelling policies at national and local levels (24–26). Evidence from two recent systematic reviews suggests labelling has positive effects on consumer dietary intake (27, 28) and industry practises (i.e., reformulation of menu items) (28).

While much research has been concerned with determining the effectiveness of menu labelling (27, 28), few studies report the extent to which menu labelling is implemented as designed (29–33). Of these, the predominant focus has been in the restaurant setting, with issues identified concerning poor uptake (29, 30), lack of adherence to standardised recipes and portions, and inaccurate calorie information being displayed (31–33). A recent systematic review identified factors influencing implementation in food service businesses (34). Factors were related to the external context of food businesses (e.g., consumers, legislation), internal setting of food businesses (e.g., compatibility, available information and resources) and features of the menu labelling intervention (e.g., perceived benefits, cost) (34). Few studies have explored the implementation process in the workplace (35, 36) and healthcare setting (25). One such study in the workplace found variation in the proportion of canteen menu items labelled with calories (50–99%) and issues relating to accuracy of this information (35). Thus far, no study has examined adherence to menu labelling policies in the healthcare setting.

With employees now spending longer periods of time in the work environment (37–39) and consuming meals prepared in the workplace canteen (40, 41), the health and well-being of employees has moved to the forefront of organisational agendas. In particular, the healthcare industry, being one of the largest employers in many countries, increasingly recognises their leadership role in serving as public health role models as well as health promotion advocates (42). In an effort to promote staff health and wellbeing, and to act as an exemplar across the public service, the Irish Health Service Executive (HSE) introduced a calorie posting policy in 2015 (43). The HSE manages the delivery of all public health services in the Republic of Ireland (ROI) and is the largest employer in the state with over 2,500 workplaces. The policy, which applies to all publicly funded health services, aims to promote awareness and increase consumption of healthier food and drink choices among HSE staff and the visiting public, by highlighting the calorie content of food and drinks provided in HSE facilities (43). Since its introduction in 2015, progress reports suggest inconsistent implementation of the policy across hospitals in Ireland (HSE, personal communication, October, 2018).

With growing evidence that fidelity of implementation is associated with success in achieving intervention outcomes (44–47), a greater understanding of the factors influencing menu labelling implementation in the healthcare setting is required. Research highlights that the process involved in the development and implementation of labelling policies is often context-specific, non-linear and shaped by many different stakeholders and factors (48). The purpose of the current study was to explore the factors that influenced fidelity to a calorie posting policy in Irish public hospitals. To this end, the study objectives were to assess the levels of implementation fidelity to the policy and to identify the perceived factors influencing implementation, and in particular, factors specific to fidelity. This study illustrates methods, applications of theory, and potentially salient factors that may inform future implementation efforts of calorie posting.

## Methods

### Study Design

**Figure 1:**
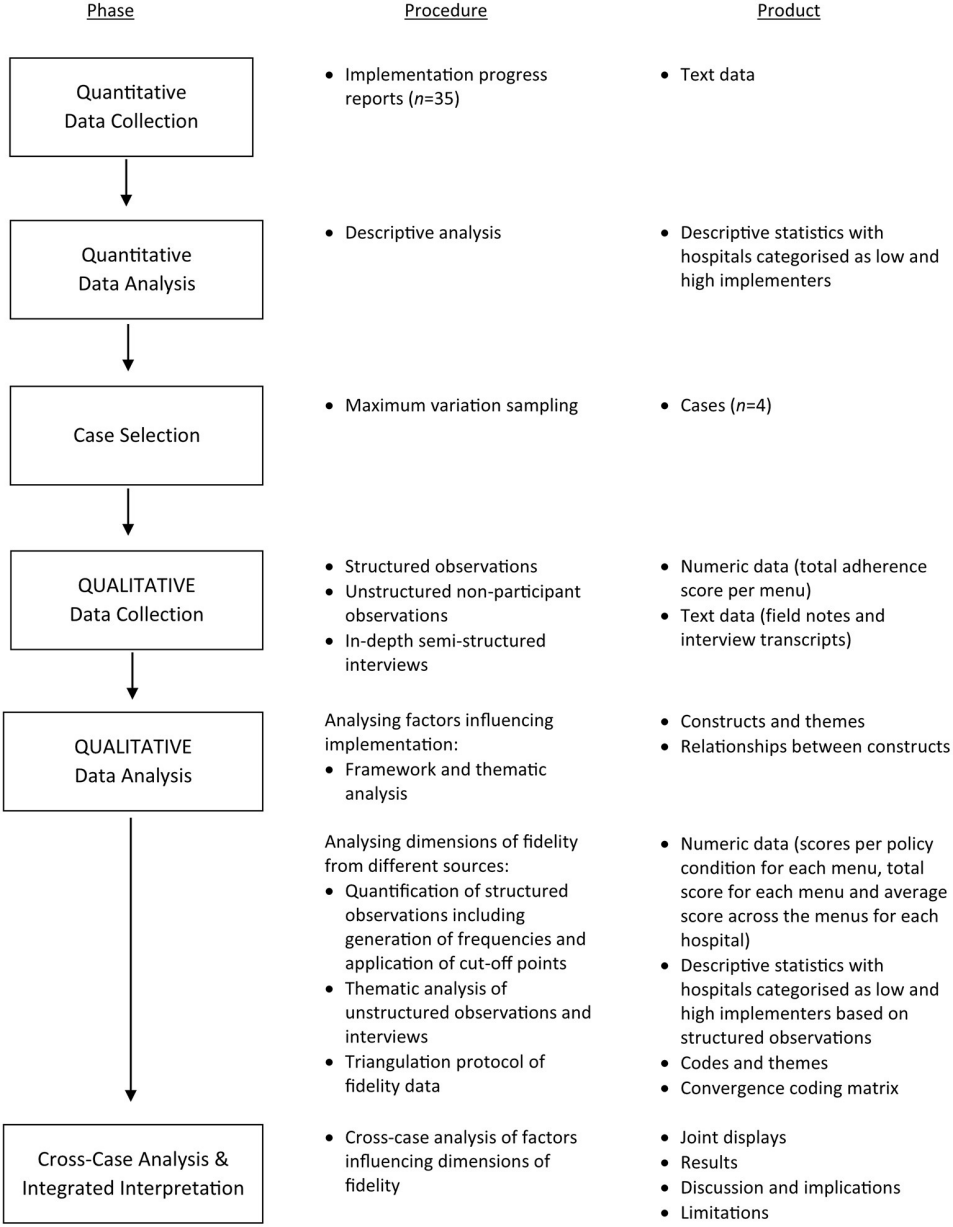
Diagrammatic representation of the mixed methods sequential explanatory design.

The study employed an explanatory sequential mixed methods design (quant → QUAL), with a nested case study for the qualitative component (49). Quantitative data on implementation progress were analysed first, while the qualitative data were collected and analysed second in sequence. The quantitative results provided an initial picture of implementation fidelity across hospitals, while the qualitative analysis refined our understanding by exploring stakeholders' views of the factors that influenced implementation, and more specifically fidelity. In terms of methods, the quantitative and qualitative phases were connected when purposefully selecting cases (hospitals) for the nested case study (50). Finally, the quantitative and qualitative results were integrated during the interpretation of the primary outcome, implementation fidelity as indicated by adherence to the HSE Calorie Posting Policy in hospital staff canteens (see Figure 1 for a diagram of the mixed methods sequential explanatory design).

The Consolidated Framework for Implementation Research (CFIR) was used to guide qualitative data collection and analysis. It is a comprehensive, meta-theoretical framework which guides systematic assessment of multi-level influences on implementation (51, 52). The CFIR consists of 39 constructs organised into five major domains: 1. Characteristics of the Intervention (e.g., Evidence Strength & Quality, Relative Advantage), 2. Outer Setting (e.g., Consumer Needs & Resources, External Policies & Incentives), 3. Inner Setting (e.g., Tension for Change, Compatibility), 4. Characteristics of Individuals (e.g., Self-Efficacy, Knowledge & Beliefs), and 5. Process (e.g., Planning, Reflecting & Evaluating). Ethical approval was obtained from the National University of Ireland Galway (ref: 18-Oct-05) and the four participating hospitals. The study has been reported according to published best practises for mixed methods studies (53) (see Supplementary Material 1).

### Policy Description

In September 2015, the HSE Calorie Posting Policy was introduced to promote awareness and increase consumption of healthier food and drink choices amongst HSE staff and the visiting public (43). The policy specified the following four conditions must be adhered to in implementing calorie posting:

- Condition 1: Calorie posting is in place for all food and drink items on sale.
- Condition 2: Calorie information is displayed clearly at the “point of choice” for the consumer.
- Condition 3: Calorie information is displayed per standard portion or per meal.
- Condition 4: Information on how many calories an average person needs in a day is prominently displayed to help consumers better understand calorie information.

For more detail on the policy, using the Template for Intervention Description and Replication in Population Health and Policy Interventions Guidelines for Intervention Reporting (54), see the published protocol (55).

### Quantitative Phase

The sample consisted of 35 HSE-funded adult acute public hospitals in the ROI with internal catering services who had experience of implementing calorie posting, after the exclusion criteria were applied. Hospitals with external catering services (*n* = 2) were excluded as these caterers have different levels of resourcing to implement calorie posting which may not be dependent on hospital resources. Specialist hospitals (maternity, paediatric etc., *n* = 7) were excluded as they were not considered representative of adult acute public hospitals. Hospitals with no experience of calorie posting in staff canteens were also excluded (*n* = 6). Implementation progress reports were provided by the 35 hospitals (October 2018), and were analysed to assess implementation fidelity and inform case sampling. These reports described whether calories were posted on the breakfast menu only or across the full menu (i.e., adherence to policy condition one). Based on this information, hospitals were categorised as low implementers (i.e., calories on breakfast menu only) and high implementers (i.e., calories across full menu) of the calorie posting policy.

### Qualitative Phase

A multiple case study design was used in the qualitative phase. In link with the explanatory sequential design, quantitative data on implementation fidelity at hospitals were used to inform the sampling criteria for cases. Maximum variation sampling was used to select four hospitals with high and low levels of implementation, and variation in terms of geographic location, hospital size, complexity of care provided and hospital type (non-voluntary hospitals–owned and funded by the HSE, and voluntary hospitals–funded but not owned by the HSE). According to Stake (56), a minimum of four cases is recommended; while Creswell (57) suggests no more than four cases to allow individual cases to be adequately explored. Thereafter, a combination of snowball and purposive sampling techniques were used to recruit participants with roles and responsibilities for implementation, including hospital direct (e.g., catering management and staff) and indirect (e.g., hospital senior management, dietitians, health promotion and improvement staff) stakeholders.

Data were collected by the lead investigator (CKer) using multiple methods and included: (1) structured observations; (2) unstructured non-participant observations; (3) in-depth semi-structured interviews. Structured observations, using an observer-rated implementation checklist developed by the study team and refined through initial testing at a pilot hospital, were used to assess implementation of the policy conditions for each menu. Observations were carried out over a 12-h period in staff canteens. Adherence to the four policy conditions for each menu was rated (by CKer) on a scale between 0 and 2 (0 = *no*, 1 = *partially*, 2 = *yes*); thus, generating a total adherence score per menu ranging from 0 (*no condition implemented*) to 8 (*all four conditions fully implemented*). The study also used overt, unstructured non-participant observations in staff kitchens/canteens. Field notes were recorded during and/or immediately following the observations. Interviews with direct and indirect stakeholders at each hospital were also conducted. The interview guide was informed by CFIR constructs identified by a recent systematic review of menu labelling determinants (34). Interviews were audio-recorded and transcribed verbatim.

### Analysis

The first phase of analysis involved identifying factors influencing implementation, including factors specific to fidelity. In the second phase, data on fidelity were analysed. Findings from both phases were then integrated. The analysis was performed at two levels: within each case and then across the cases (56, 58).

#### Factors Influencing Implementation

Using NVivo 11 software for data management, framework analysis (59, 60) was performed, with the CFIR as the *a priori* framework. Thematic analysis was used where data did not fit within the framework (61), and new constructs were added to the codebook. See constructs denoted with an asterisks in Supplementary Material 2. Two researchers (CKer and SMH) independently coded data from one hospital to check for coding consistency and to modify CFIR construct definitions where necessary. For example, the construct *Patient Needs & Resources* in the *Outer Setting* domain was modified to *Consumer Needs & Resources* to reflect that this was a staff facing intervention (see Supplementary Material 2). Once agreed, the lead investigator (CKer) analysed data from the remaining hospitals (see Supplementary Material 2 for final codebook). Using causation coding (61), relationships between constructs were also identified by the lead investigator (CKer) based on interview and observational data for each hospital and were checked independently by another researcher (CR). A consensus-building coding process was adopted throughout the analysis (61).

#### Implementation Fidelity

To assess fidelity, descriptive statistics were calculated for each hospital using data from structured observations. This included scores per policy condition for each menu and a total score for each menu. An average score across the menus for each hospital was then calculated. Finally, hospitals were categorised into high (average score >4) and low (average score ≤ 4) levels of implementation fidelity by applying cut-off points. Similar to other research (62), these cut-off points were based on minimally accepted practises following discussion and consensus within the research group.

These data were supplemented with other sources of evidence relating to implementation fidelity, not specified in the protocol (55). Information on fidelity was generated during semi-structured interviews and unstructured observations without formal assessment or questioning. Data were analysed thematically to further explore implementation fidelity at each hospital (61).

A triangulation protocol was used to integrate fidelity findings from four sources (i.e. HSE progress reports, structured observations, semi-structured interviews and unstructured observations) (63–65). A “convergence coding matrix” was created with independent findings (as rows) mapped across the data sources (columns). The relationships between data was categorised as: (1) silence (only one data source out of the two being compared contained data on a particular finding), (2) dissonance (conflicting findings in the data), (3) partial agreement (complementarity between data) or (4) agreement (convergence in the data) (65). The triangulation process facilitated the identification of meta-themes that cut across the four sources of fidelity data (63, 64). In line with best practise (63, 64), multiple researchers (CKer, SMH and ET) with combined expertise in qualitative and quantitative methods worked together during triangulation.

#### Cross-Case Analysis and Integrated Interpretation

Initially, as per the protocol and purposive sampling strategy, the study set out to identify determinants of implementation success by comparing hospitals with high and low levels of fidelity based on the progress reports (55). However, the integrated analysis of fidelity from multiple sources called into question this dichotomy. As a result, the cross-case analysis focused on identifying patterns of fidelity and influencing factors across hospitals. For the cross-case analysis, data were analysed for each hospital first, as outlined above (i.e., examining fidelity per hospital, examining CFIR constructs per hospital). Then findings were compared across hospitals using matrices (displaying results in rows and hospitals in columns). Quantitative and qualitative data were integrated using joint displays to identify meaningful similarities, differences and case-specific experiences (50). In a deviation from the protocol (55), the perspectives of different stakeholders were not compared through formal analysis; however, the type of stakeholder was taken into account when presenting commonly cited influencing factors.

## Results

Of the 35 hospitals included in the analysis of fidelity, seven hospitals were categorised as low implementers (i.e., calories on breakfast menu only) and 28 hospitals were high implementers (i.e., calories across full menu) of the calorie posting policy. Characteristics of the four cases are provided in Table 1. The following sections describe factors influencing implementation and within that, factors linked to fidelity and implementation fidelity across hospitals. In an effort to minimise repetition/fragmentation, the results of the cross-case analysis are weaved throughout the results section.

**Table 1:** Hospital profiles and unstructured observation/interview characteristics.

**Hospital profile**	**Hospital 1**	**Hospital 2**	**Hospital 3**	**Hospital 4**
Model^a^	3	4	3	4
Type^b^	Non-voluntary	Non-voluntary	Non-voluntary	Voluntary
Size	<1500 WTE staff	>1500 WTE staff	<1500 WTE staff	>1500 WTE staff
Menus served in staff canteen	Breakfast, lunch & tea	Breakfast & lunch	Breakfast & lunch	Breakfast & lunch
Observation duration and location	9 hrs (staff kitchen & canteen)	7.5 hrs (staff kitchen & canteen)	7 hrs (staff kitchen & canteen)	8 hrs (staff kitchen & canteen)
Interview numbers and stakeholder type	9 (6 direct & 3 indirect)	7 (4 direct & 3 indirect)	6 (5 direct & 2 indirect)^c^	8 (6 direct & 2 indirect)
Interview average duration and mode	36 min (face-to-face & telephone)	33 min (face-to-face & telephone)	34 min (face-to-face & telephone)	35 min (face-to-face & telephone)

a * Hospital model–hospitals are categorised into one of four models which determines the complexity of patient care delivered in each hospital. Model 3 is a general hospital, while model 4 is a tertiary hospital - university hospital or specialist centre*.

b * Hospital type–non-voluntary hospitals (owned and funded by the HSE) and voluntary hospitals (funded but not owned by the HSE)*.

c * Two direct stakeholders were interviewed together at their request due to limited time availability*.

### Factors Influencing Implementation

Factors influencing implementation of the calorie posting policy were coded to constructs across four CFIR domains (as outlined below), with no data coded to the domain which focuses on individual-level constructs (i.e., *Characteristics of Individuals*) (see Table 2). Most acted simultaneously as barriers and facilitators within and across hospitals. For detailed within and across-case results, including themes and illustrative quotes/observational field notes, see Supplementary Material 3. A matrix of perceived barriers, facilitators and recommendations for future implementation efforts coded to domains and constructs by hospital are provided in Supplementary Materials 4, 5 and 6, respectively. While some factors influencing implementation operated independently, others acted in combination (i.e., with unidirectional and bi-directional relationships). Supplementary Material 7 provides detail of factors which operated in combination, including illustrative quotes/observational field notes. A narrative summary of commonly occurring factors and how they manifested within and across hospitals is presented below.

**Table 2:** Summary of perceived barriers and facilitators to implementation of calorie posting policy.

**Domains & Constructs**	**Hospitals that identified facilitators**	**Hospitals that identified barriers**
**Intervention Characteristics**	**1, 4**	**1, 2, 3, 4**
Intervention Source	No data	No data
Evidence Strength & Quality	No data	No data
Relative Advantage	4	2
Adaptability	No data	No data
Trialability	No data	No data
Complexity	No data	1^×^, 2, 3, 4^×^
Design Quality & Packaging	1	1^×^, 2^×^, 3^×^, 4^×^
Cost	No data	1, 2, 4
**Outer Setting**	**1, 2, 3, 4**	**1, 2, 3, 4**
Consumer Needs & Resources (OS)	1, 2, 4	No data
Cosmopolitanism	1, 3	1, 2
Peer Pressure	1, 2, 3	No data
External Policy & Incentives	1, 2, 3, 4	1, 2, 3^×^, 4
Economic Climate^*^	No data	1
Educational System^*^	4	2
Culture (OS)^**^	No data	2, 4^×^
**Inner Setting**	**1, 2, 3, 4**	**1, 2, 3, 4**
Structural Characteristics	1, 2, 3, 4	1^×^, 2^×^, 3, 4^×^
Networks & Communications	1, 2, 3, 4	1, 2, 3, 4
Culture (IS)	1, 3, 4^×^	1^×^, 2^×^, 3, 4^×^
Consumer Needs & Resources (IS)^**^	1, 2, 4	1^×^, 2^×^, 3^×^, 4^×^
Implementation Climate	1, 2, 3, 4	1, 2, 3, 4
Tension for Change	1, 2, 4^×^	1, 2^×^, 4
Compatibility	1, 2, 3, 4^×^	1, 2, 3, 4^×^
Relative Priority	4	1^×^, 2^×^, 3, 4
Hospital Incentives & Rewards	3	2, 3, 4
Goals & Feedback	1	No data
Learning Climate	1, 2, 4	No data
Readiness for Implementation	1, 2, 3, 4	1, 2, 3, 4
Leadership Support	1^×^, 2, 3, 4^×^	1, 2^×^, 3, 4^×^
Available Resources	1, 2	1^×^, 2^×^, 3, 4^×^
Access to Knowledge & Information	1, 2, 3, 4	1^×^, 2^×^, 3^×^, 4^×^
**Characteristics of Individuals**	**No data**	**No data**
**Process**	**1, 2, 3, 4**	**1, 2, 3, 4**
Planning	1, 4	1, 2, 3
Engaging	1, 2, 3, 4	1, 2, 3, 4
Opinion Leaders	No data	No data
Formally Appointed Internal Implementation Leaders	1^×^, 2, 3, 4^×^	1, 2^×^, 3, 4^×^
Champions	3	3
Internal Key Stakeholders^*^	1, 2, 3, 4^×^	1^×^, 2^×^, 3, 4^×^
Consumers (IS)^*^	1^×^	1^×^, 2^×^, 3^×^, 4^×^
External Key Stakeholders^*^	1, 2, 3, 4	1, 2^×^, 3, 4^×^
External Change Agents	1, 2, 3^×^, 4^×^	1, 2, 3^×^, 4^×^
Executing	No data	1, 2
Reflecting & Evaluating	1^×^, 4^×^	1, 2^×^, 3, 4^×^
Adapting the Intervention^*^	1	No data
Adapting the Organisation^*^	1, 2, 3, 4	No data
Scaling Up^*^	1, 2, 3, 4	No data
Strategy^**^	3, 4	No data

* * new construct generated inductively from recent systematic review (34)*;

** * new construct generated inductively from the data; ^×^ factor influencing fidelity within hospital*.

*IS, inner setting; OS, outer setting*.

#### Domain: Inner Setting

The internal environment of the hospital was both a barrier and facilitator to implementation across all hospitals. Factors such as the general level of implementation receptivity and readiness presented obstacles and (future) opportunities for implementation. Intervention compatibility with existing work processes exerted a positive and negative influence across all hospitals. Factors which undermined compatibility included changing ingredients or menus, consumers requesting menu variation and required ingredients being unavailable from suppliers. A dietitian manager described this challenge:

…* if you're working where you're changing menus regularly… there is extra resource requirement on an ongoing basis to maintain calorie posting in place. It's not like you do it once and that's it…* [Dietitian Manager]

Across all hospitals, calorie posting implementation aligned with existing practises or initiatives and thus, made implementation efforts easier. Existing practises or initiatives which enhanced compatibility, noted in interviews and observations, included: undertaking nutritional analysis of patient menus, serving similar meals to patients and staff, posting allergens and implementing workplace health initiatives. In some hospitals, compatibility with work processes led to access to information required for calorie posting implementation. As noted by a senior dietitian:

*Some of the food that's available for the patient menus is the same as what's served in the canteen. So a lot of the time while she was processing the patient menus she could just take that information and put it on to the calorie posting for the canteen*. [Senior Dietitian]

The lack of perceived importance of calorie posting implementation was a barrier across all hospitals. Stakeholders spoke about calorie posting not being a priority, due to greater importance being placed on patient care, food safety, allergens and other initiatives. In all hospitals, a robust culture of patient-centred care decreased the priority of implementing interventions for staff health and well-being. A dietitian described this challenge:

*I don't think there was a huge amount of emphases on calorie posting in the canteen before we had to do it for the patient menus. When it had to be done for the patient menus it was a big priority. Being done for the staff it was take it or leave it*. [Senior Dietitian]

The lack of access to knowledge and information about calorie posting and the lack of, or inadequate, training for catering staff was another cited barrier across all hospitals. The reason cited for lack of information was a poorly designed policy with no supporting materials and/or best practise guidelines. In some hospitals, direct stakeholders spoke about overcoming this barrier by accessing information through Google. Across all hospitals, stakeholders highlighted the need for access to knowledge and information to assist with implementation efforts in the future. Recommendations included providing access to structured training for catering staff and clear information on calorie posting implementation. As noted by one member of catering staff:

…* there was no supporting information to help us. We were all kind of working at it ourselves through the internet… It was up to yourself to get on with it… we really would have benefited from more information and guidance*. [Catering Staff]

Support from leadership was a facilitator across all hospitals. Hospital management were supportive of policy implementation and in some hospitals, provided recognition for implementation efforts and funding for nutrition analysis software. In some hospitals, support from hospital management was considered an important factor for engaging catering staff and management in calorie posting implementation. As one dietitian manager described:

*And thankfully hospital management here have been very supportive and they've agreed to fund the licence. We can't do good analysis… without a nutritional analysis software package…* [Dietitian Manager]

Stakeholders also highlighted the supportive role of catering management and noted in most hospitals, implementation efforts were led by the assistant catering manager with support from students on placement. Catering management support was driven by a staff-centred or improvement culture within the catering department, improving consumer health, external accreditation/awards and audit/monitoring via researcher visit. As one senior hospital manager described:

…* there's a culture within the catering department firstly where they just love to serve staff and they want to give them the nicest and the best food possible… So this was an easy one really because we just knew it was going to build on a good tradition and a good sound belief that they could do better*. [Clinical Support Services Director]

The needs and preferences of consumers using the canteen were identified as barriers to implementation. Direct stakeholders discussed the lack of demand for standardised portions, with consumers wanting value for money and, in particular, male consumers having preference for larger portions. Across all hospitals, this led to consumer lack of compliance with standardised portions (e.g., asking for more). As noted by one head chef:

*People always want bang for their buck as they say. Some people have smaller appetites and some people have bigger appetites. You can standardise as much as you like within a hot serve but you are going to be dealing with people that will look for more*. [Head Chef]

#### Domain: Process

The process of implementing calorie posting was both a barrier and facilitator across all hospitals. In particular, stakeholders highlighted challenges in engaging catering staff and dietitians in implementation. The lack of or limited engagement of catering staff was attributed, in large part, to catering management not involving staff in implementation. Inadequate staffing levels was another reason cited by stakeholders for lack of or limited engagement of catering staff and dietitians. With competing priorities such as patient services, existing staff had limited time to implement calorie posting. A dietitian manager described these challenges:

*Patients are absolutely the priority because we don't have enough staff across the board. It's extremely stretched. Yeah. We don't have resources to take on anything outside. We don't even have the resources to manage the clinical service never mind the health promotion type aspects which are very, very important but we just have to prioritise the patient work*. [Dietitian Manager]

Across all hospitals, stakeholders highlighted the positive influence of engaging individuals who were affiliated with an outside entity. Examples of successful engagement with external change agents included obtaining an award from the Irish Heart Foundation for implementing calorie posting, and receiving support from nutrition students on placement in terms of standardising recipes and undertaking nutritional analysis. Stakeholders highlighted that engaging students in implementation compensated for the limited involvement of catering/dietetic management and staff due to time constraints. In addition, the successful engagement of students and the Irish Heart Foundation facilitated direct and indirect stakeholder engagement in hospitals and provided access to information required for implementation. As a catering manager and staff member described:

…* my student is doing it because I haven't got the time directly to give it…* [Catering Manager]

*Oh the Irish Heart Foundation award was very significant… There was huge buy-in then within the catering department*. [Catering Staff]

#### Domain: Outer Setting

The external environment to the hospital facilitated implementation across all hospitals. In particular, external policies and incentives such as (impending) menu labelling legislation, HSE national policy and external accreditation/awards exerted a positive influence on implementation. These external policies and incentives led to hospital/catering management support and engagement, and created an urgency to implement calorie posting. In some hospitals, stakeholders noted that policy requirements to implement calorie posting were more important than consumer demand/interest. As a catering and dietetic manager described:

…* external accreditation holds a lot of weight… it makes it easier to negotiate and to kind of keep a door open… particularly at the executive level with the hospital CEO…* [Catering Manager]

…* it's our national policy the calorie posting in terms of the HSE… Regardless of whether or not consumers want it… its policy so it should be implemented*. [Dietitian Manager]

#### Domain: Intervention Characteristics

Features of the calorie posting policy presented barriers to implementation across all hospitals. In particular, the perceived quality of policy packaging and presentation, including materials and supports available, had a negative influence. Stakeholders described the lack of supporting materials, impractical display options and non-user friendly design as obstacles to implementation. Stakeholders recommended adapting the intervention for future implementation efforts, including introducing a template for calorie posting to help standardise across all hospitals and a more practical calorie display method such as menus electronically displayed with calories. As one catering manager highlighted:

*The logistics of trying to keep the poster updated with new menu items and calories…just doesn't work… We've actually applied for funding only last week for a digital menu board… once the information is typed up it will be easier to maintain*. [Catering Manager]

### Factors Influencing Fidelity

There were barriers and enablers to fidelity to the policy overall and specific dimensions which contributed to an overall pattern of partial adherence evident across hospitals (see Supplementary Material 8).

In some hospitals, inter-related factors cited by direct stakeholders which negatively influenced adherence to the overall policy included lack of internal monitoring of implementation progress over time due to hospital tick-box culture. As highlighted by one head chef:

*I think they take on these projects and they look and sound good… They're ticking boxes and that you know… But it's a tick the box. It looks done. But is it really done? No one really checks… I don't think we have fully implemented calorie posting yet*. [Executive Head Chef]

In some hospitals, catering staff and management reported the lack of adherence to calorie posting across all food and drink items on sale (policy conditions 1) was due to impractical calorie display methods. As one catering supervisor highlighted:

*Now there's still a lot of calories that's not on the big chart. It's difficult to keep it updated with new menu items as need to re-do poster each time*. [Catering Supervisor]

Across all hospitals, lack of adherence to standardised portions was attributed, in large part, to lack of consumer demand for standardised portions and expressed preference for larger portions. As one catering manager described:

*So like there is a standard portion but like when somebody asks for more they receive more…* [Catering Manager]

In some hospitals, indirect stakeholders highlighted lack of training for catering staff was a contributing factor to lack of adherence to standardised recipes and portions. Direct stakeholders in one hospital reported the lack of internal monitoring hindered adherence to standardised recipes; while, direct stakeholders in another hospital noted the presence of internal monitoring enabled greater adherence to standardised portions. As noted by one dietitian manager:

*Not giving correct portions is more to do with lack of training, than resistance from the catering people*. [Dietitian Manager]

Inaccurate calorie information was largely attributed to lack of adherence to standardised recipes and/or portions across all hospitals. Direct stakeholders in some hospitals highlighted challenges in serving precise standardised quantities of composite dishes such as curries and stews. Student supervision/input was also linked to accuracy of calorie information. Indirect stakeholders in one hospital reported inaccurate calorie information was due to lack of supervision by dietitians; while, direct stakeholders in another hospital noted accurate calorie information was linked to catering management providing student input/guidance. Tied to this, stakeholder level of nutrition knowledge, in particular catering staff and students, was also highlighted by indirect stakeholders as an influential factor in terms of achieving accurate calorie information. Stakeholders described these challenges in obtaining accurate calorie information:

*So two ladles of chicken curry could contain different quantities of chicken or veg… So we are not getting an accurate calorie count there…* [Executive Head Chef]

*I noticed on the posters like some calories straight off as a dietitian I could tell that's not right, that's wrong… because there was no direct supervision from a dietitian I think there are probably a lot of errors in the calculations*. [Dietitian Manager]

### Implementation Fidelity Across Hospitals

Most comparisons between data sources resulted in “silence”, with the remaining comparisons in “agreement” and some instances of “dissonance” (see Supplementary Material 9). In terms of adherence to the four policy conditions, conflicting findings were noted between data sources: two hospitals were each categorised as having high and low levels of fidelity based on structured observations; while interviews revealed partial adherence across all hospitals. Conflicting findings on adherence to calorie posting across all food and drink items on sale (policy conditions 1) were also noted between the HSE progress reports and that of structured observations and interviews.

Both structured observations and interviews showed a consistent pattern of low adherence to calorie posting across all food and drink items on sale (policy conditions 1) and calorie information displayed per standard portion or per meal (policy condition 3) across all hospitals. There was variability in adherence to the remaining policy conditions (2 and 4) across hospitals.

Overall, the integrated analysis of fidelity findings from four sources showed a pattern of partial adherence to the calorie posting policy across all four hospitals, with evidence of variation in specific fidelity dimensions (see Table 3).

**Table 3:** Summary of indicators of fidelity according to data sources.

		**Fidelity Dimension**	**Hospital 1**	**Hospital 2**	**Hospital 3**	**Hospital 4**
**Case Selection**	**HSE Progress Reports** ^a^	Adherence to policy condition 1: calories posted on breakfast menu only (low) or across full menu (high)	High	Low	Low	High
**Study Data Collection**	**Quantitative findings from structured observations** ^b^	Adherence to four policy conditions across each menu	Low (avg. score 3) Across menus: Condition 1 – avg. score 1 Condition 2 – avg. score 1 Condition 3 – avg. score 1 Condition 4 – avg. score 0	Low (avg. score 3) Across menus: Condition 1 – avg. score 1 Condition 2 – avg. score 0 Condition 3 – avg. score 1 Condition 4 – avg. score 1	High (avg. score 5) Across menus: Condition 1 – avg. score 1 Condition 2 – avg. score 2 Condition 3 – avg. score 1 Condition 4 – avg. score 1	High (avg. score 5) Across menus: Condition 1 – avg. score 1 Condition 2 – avg. score 1 Condition 3 – avg. score 1 Condition 4 – avg. score 2
	**Qualitative findings from observations and interviews**	Adherence to overall policy (i.e. four conditions)	Perceived partial implementation of policy over time (previous full implementation), including: • Calories not in place for all menu items (policy condition 1) • not clearly displayed at “point of choice” (policy condition 2) and, • not always displayed per standard portion or per meal (policy condition 3).	Perceived partial implementation of policy, including: • limited calories on display from previous implementation efforts (policy condition 1) and, • not always displayed per standard portion or per meal (policy condition 3).	Perceived partial implementation of policy over time (previous full implementation), including: • Calories not in place for all menu items (policy condition 1) and, • not always displayed per standard portion or per meal (policy condition 3).	Perceived partial implementation of policy, including: • Calories not in place for all menu items (policy condition 1) and, • not always displayed per standard portion or per meal (policy condition 3).
		Adherence to standardised recipes or portions	Perceived lack of adherence to standardised portions but not recipes: • larger portions being served. • Also noted during unstructured observation.	Perceived lack of adherence to standardised portions and recipes: • larger portions being served. • Also noted during unstructured observation.	Perceived lack of adherence to standardised portions and recipes: • larger portions being served. • Also noted during unstructured observation.	Perceived lack of adherence to standardised portions and recipes: • larger portions being served. • Not noted during unstructured observation.
		Accuracy of calorie information	Perceived inaccuracies in calorie information. Also noted during unstructured observation.	Perceived inaccuracies in calorie information. Also noted during unstructured observation.	Perceived inaccuracies in calorie information. Also noted during unstructured observation.	Perceived inaccuracies in calorie information. Not noted during unstructured observation.
		Fidelity (overall interpretation)	Partial	Partial	Partial	Partial

a * HSE progress reports (from October 2018) were provided by individual hospitals. Information on adherence to policy condition one (i.e. calorie posting is in place for all food and drink items on sale) were provided in these reports. Based on the information provided, hospitals were broadly categorised into low implementers (i.e., calories on breakfast menu only) and high implementers (i.e., calories across full menu) of the calorie posting policy*.

b * Structured observations using an observer-rated implementation checklist were used to assess adherence to the four specific conditions outlined in the HSE Calorie Posting Policy. For each individual menu, adherence to each of the four policy conditions were rated on a scale between 0 and 2 (0 = no, 1 = partially, 2 = yes), thus generating a total adherence score per menu ranging from 0 (no condition implemented) to 8 (all four conditions fully implemented). Hospitals with an average score across menus >4 were categorised as having high levels of implementation fidelity, while hospitals with an average score across menus ≤ 4 were categorised as having low levels of implementation fidelity*.

## Discussion

This mixed methods study explored factors influencing fidelity to a calorie posting policy in Irish acute public hospitals. Integrated findings from multiple data sources revealed different levels of fidelity depending on the aspect of the policy under consideration and the source of data. There was an overall pattern of partial adherence across hospitals. Across all hospitals, there was a consistent pattern of low adherence to calorie posting across all menu items on sale, low adherence to calorie information displayed per standard portion or per meal, low adherence to standardised recipes/portions, and inaccurate calorie information. Factors influencing fidelity operated independently and in combination, and were evident across four domains: the internal and external environment of the hospital, the process involved in implementation and features of the calorie posting policy. For example, external policies and incentives (e.g., national policy, monitoring) and features of the calorie posting policy (e.g., availability of supporting materials) influenced hospital receptivity and readiness to implement (e.g., leadership support, access to knowledge and information, perceived importance of calorie posting implementation) and subsequently, the process involved in implementation (e.g., engaging relevant stakeholders). Findings highlight the complex and dynamic influences at play during implementation.

Recent research highlights a nuanced relationship between direct (i.e., observer report) and indirect (i.e., self-report) measures of fidelity, where agreement between both measures is stronger for some intervention components than others (66–68). The current study found good agreement between direct and indirect measures across most dimensions of fidelity; however, there was some instances of disagreement. This included conflicting findings between HSE progress reports (October 2018) and that of structured observations and interviews (September/October 2019), which may reflect differences in fidelity assessment across time (68). Disagreement between structured observations and interviews was also evident, where stakeholders from two hospitals rated themselves lower than the observer as regards adherence to the four policy conditions. This finding is in contrast with previous research which show individuals report higher fidelity than observers (69–73). A possible explanation for this may include differences in conceptualisation of high and low fidelity by researchers and implementers. For example, cut-off points were decided arbitrarily by the research team and may have benefited from stakeholder engagement in this process.

Analysis of fidelity using direct and indirect measures highlighted core intervention components not specified in the calorie posting policy, including adherence to standardised recipes/portions and accuracy of calorie information. This may reflect the low level of specificity with which essential components (i.e., the four policy conditions) were defined (74, 75) or the lack of interrogation of the intervention logic for calorie posting in the literature to enable specification of intervention components (76, 77). Nevertheless, research shows clear specificity of what the intervention or policy entails is necessary to ensure effective implementation (44, 78). Hawes (76, 79) also argues that intervention components should be clearly identified as relating to form (i.e., variable aspect of the intervention–calorie display method) and function (i.e., fixed aspect of the intervention–accurate calorie information per standard portion or per meal). So there is standardisation by function rather than form across sites, which allows for adaptation to context while maintaining fidelity (79).

Similar to research on other innovations in the health care setting (80), the lack of access to information and training was an obstacle to implementation. Studies show training of key staff is a predictor of implementation success (81–84). Although expressed as a facilitator to implementation, accessing information via the web may be an unreliable source of information and thus, may have contributed to inaccurate calorie information. Study participants were unaware of a “Calorie Posting Toolkit” which had been designed to assist hospitals when implementing the policy (85); this may have contributed to partial adherence across hospitals. According to a recent study (86), implementation of evidence-based interventions in real-world settings become a futile effort when effective strategies to help implementation are not used.

There is evidence to suggest that organisations with cultures that are more supportive of employee health and well-being are more effective in implementing evidence-based practises (87). In the current study a lower priority was placed on implementing interventions such as calorie posting designed for staff health and well-being due to inadequate staffing levels and a culture which prioritised patient-centred care. While this was a common barrier to implementation across all hospitals, it was specifically linked to lack of adherence to the overall policy in some hospitals. These findings are similar to previous research which indicate that health care resources are under pressure and subsequently, patient-based tasks are the priority, with little or no time available for staff to implement new or existing evidence-based practises (80). Furthermore, other research has shown that lack of perceived importance due to limited resource availability is associated with low levels of implementation success (88–91).

In response to staff shortages and time restraints, students on placement were engaged in calorie posting implementation across all hospitals. Although the supportive role of external change agents is well documented (92), the current study identified issues with lack of appropriate student supervision and their varying levels of nutrition knowledge. According to a recent study (93), intervention responsibility and accountability need to be considered when engaging change agents. The authors of that study and others highlight the potential harm of over-reliance on these individuals and its impact on sustainable intervention practise (51, 93, 94).

The needs and preferences of staff utilising the canteen (i.e., consumers) was perceived to undermine adherence to standardised portions and subsequently, result in inaccurate calorie information. As noted in a recent systematic review of menu labelling determinants (34), these findings may reflect the lack of consumer education about how to utilise menu labelling effectively. The CFIR places the service user (i.e., patients/consumers) under the outer setting domain, suggesting their peripheral role in the implementation process (95). In the current study, the addition of a new construct on consumer needs and preferences to the inner setting domain demonstrated the central role of consumers in the implementation process. This is in line with recommendations in the implementation science literature (95–97), were greater consideration of the service user role in implementation is required.

In general, policies and incentives external to hospitals exerted a positive influence by garnering hospital/catering management support and generating an urgency to implement calorie posting. These findings reflect key aspects of institutional theory which suggest that organisations change as an adaptive response to coercion, or to strong pressures to comply with rules, regulations, and mandates (98, 99). Research also shows organisations will implement innovations to comply with accrediting bodies (100). While it is recognised that national policy/regulation is required to advance public health (17, 48, 101), the risk of tokenism in the form of superficial implementation needs to be averted (102). In the current study, a hospital tick-box culture led to lack of monitoring implementation which hindered policy adherence. These finding highlight the need for effective mechanisms to be put in place to ensure rigorous monitoring alongside policy (31, 103).

### Implications and Recommendations for Policy, Practice, and Research

The study findings point to the need for multi-level, multi-component strategies to maximise fidelity. Efforts to promote fidelity should begin with the initial process of policy development. “Designing for fidelity” requires adequate documentation of intervention components and a description of activities so that they can be replicated (104). Ensuring the availability of supporting materials, such as a calorie posting toolkit, and the provision of training opportunities are necessary to equip stakeholders with the required knowledge and information (86, 105). For these resources to be properly utilised, stakeholders involved in implementation need to know how to access and interpret them (80). Supporting implementation also requires consideration of human resources and its impact on time and capacity to implement new innovations (106, 107). In the face of competing demands, there is a need to prioritise staff health and create a culture that promotes and supports a healthy workforce (108).

To maximise fidelity and reduce “intervention drift”, implementation needs to be adequately and continuously monitored via internal and external sources (68, 77, 104, 109, 110). A feedback loop is also required along with encouragement, reward or recognition for implementation efforts (104). Both monitoring and the provision of feedback are considered key to maximising stakeholder engagement and contributing to effective implementation (104). The Plan-Do-Study-Act cycle is a commonly used process improvement strategy in health care settings, which allows organisations to initiate, evaluate and refine over a short time period (111–113). Furthermore, given the volunteer nature associated with student engagement, training and supervision must also be a priority to ensure a high degree of fidelity (114).

Few implementation models/frameworks, including the CFIR (51), recognise that different factors may influence implementation at different points in the implementation process (115). Future research should determine the factors relevant to different phases of implementation, so as to help stakeholders to anticipate and address factors in sequence or in tandem for effective implementation (34). A newly developed method called Coincidence Analysis (116), offers the potential to identify combinations of conditions that are minimally necessary or sufficient for effective implementation. Furthermore, the association between implementation strategies and shifts in fidelity to intervention core components could be examined in future studies (68).

### Limitations and Strengths

While the sample of hospitals was diverse in terms of geographic region, size and type, they were all public hospitals with internal catering services. Thus, findings may not be transferable to private hospitals and those with external catering services. In an effort to help minimise the effects of recall and response bias, data from multiple sources and types were used (58, 117). Despite the recognised benefits of using multiple measures of fidelity (77, 118), there were challenges to integrating fidelity data due to different levels or aspects of fidelity evident in different sources. A diffractive approach, whereby patterns of difference and entanglement are identified, may have offered another means to capture the complexity and messiness of fidelity findings (119). While multiple perspectives from different stakeholders were obtained, the study did not consider the consumer perspective. Finally, the study analysis goes beyond simply listing influential factors by highlighting interactions and their effect on adherence to a calorie posting policy.

## Conclusion

This study found partial adherence to a calorie posting policy across hospitals, where influential factors were multiple, and operated independently and in combination. Factors were related to the hospital internal and external environment, features of the calorie posting policy and the process involved in implementation. The importance of multiple measures of fidelity to generate accurate and more comprehensive fidelity findings was also evident. Findings point to the need for multi-level, multicomponent strategies to maximise fidelity. Future research should assess the relative importance of calorie posting determinants and the association between implementation strategies and shifts in fidelity to intervention core components.

## Data Availability Statement

The data analysed during the current study are not publicly available in order to protect the privacy and confidentiality of all study hospitals and participants.

## Ethics Statement

Ethical approval was obtained from the National University of Ireland Galway (ref: 18-Oct-05) and the four participating hospitals. Verbal consent was obtained from hospital gatekeepers to conduct the study. Written informed consent was obtained from study participants in each hospital.

## Author Contributions

CKer was the lead investigator and drafted the manuscript. CKer, CKel, CHo, ET, CHa, FG, IP, and SMH contributed to the concept and design of the study. CKer collected data and led the analysis with input from CR, SMH, CKel, and ET. All authors were involved in the interpretation of results, and critically reviewed and approved the final manuscript.

## Conflict of Interest

The authors declare that the research was conducted in the absence of any commercial or financial relationships that could be construed as a potential conflict of interest.

## Publisher's Note

All claims expressed in this article are solely those of the authors and do not necessarily represent those of their affiliated organizations, or those of the publisher, the editors and the reviewers. Any product that may be evaluated in this article, or claim that may be made by its manufacturer, is not guaranteed or endorsed by the publisher.
