# Supplementary material for: Factors Influencing Fidelity to a Calorie Posting Policy in Public Hospitals: A Mixed Methods Study
Source: Front Public Health. 2021 Aug 13;9:707668. doi: 10.3389/fpubh.2021.707668 (PMC8414889; doi:10.3389/fpubh.2021.707668)
Supplement: Supplementary file 1 [file Table_1.DOCX]

**Additional file 1: Good Reporting of A Mixed Methods Study (GRAMMS) checklist**

| **Guideline** | **Section: page** |
| --- | --- |
| Describe the justification for using a mixed methods approach to the research question | Methods – pg.7 |
| Describe the design in terms of the purpose, priority and sequence of methods | Methods – pg. 7 |
| Describe each method in terms of sampling, data collection and analysis | Methods – pg. 8-12 |
| Describe where integration has occurred, how it has occurred and who has participated in it | Methods – pg. 8, 12 |
| Describe any limitation of one method associated with the present of the other method | Discussion – pg. 24-25 |
| Describe any insights gained from mixing or integrating methods | Discussion – pg. 28 |
