# Supplementary material for: Factors Influencing Fidelity to a Calorie Posting Policy in Public Hospitals: A Mixed Methods Study
Source: Front Public Health. 2021 Aug 13;9:707668. doi: 10.3389/fpubh.2021.707668 (PMC8414889; doi:10.3389/fpubh.2021.707668)
Supplement: Supplementary file 2 [file Table_2.DOCX]

**Additional File 2. Final Codebook**

The codebook below is a result of (a) operationalisation/adaptation of a previously published CFIR codebook in this topic area [[1](#_ENREF_1)], (b) adaptation of existing construct definitions and eligibility criteria to improve coding consistency (i.e. to help distinguish constructs where unclear boundaries exist) and (c) newly developed construct definitions and eligibility criteria (from the inductive analysis).

| **Intervention Characteristics** | **Perceived characteristics of the calorie posting policy regardless of context** |
| --- | --- |
| 1. Intervention Source | Definition: Perception of stakeholders about whether the calorie posting policy is externally or internally developed.  Inclusion Criteria: Include statements about the source of the policy and the extent to which stakeholders viewed the introduction of this as internal or external to the hospital.  Exclusion Criteria: Exclude statements related to who participated in the decision making process to implement the policy and code to ‘Engaging’, as an indication of early (or late) engagement. |
| 1. Evidence Strength & Quality | Definition: Stakeholders’ perceptions of the quality and validity of evidence supporting the belief that calorie posting will have desired outcomes.  Note: Evidence Strength & Quality is a characteristic of the innovation: does it have an evidence base (before coming into the current organisation) – not the outcome in terms of effectiveness once implemented.  Inclusion Criteria: Include statements regarding awareness of evidence and the strength and quality of evidence, as well as the absence of evidence or a desire for different types of evidence (e.g. consumer feedback) instead of evidence from the literature.  Include statements that refer to the effectiveness of the intervention pre-implementation (i.e. a determinant as opposed to an outcome).  Exclusion Criteria: Exclude statements that refer to the effectiveness of the intervention post-implementation (i.e. these are outcomes, not determinants) – code statements regarding how calorie posting is working in a particular hospital to RE-INT i.e. Reflecting & Evaluating around the effectiveness of the innovation. Exclude statements regarding the receipt of evidence as an engagement strategy and code to ‘[Engaging](http://cfirguide.org/wiki/index.php?title=Engaging)’. |
| 1. Relative Advantage | Definition: Stakeholders’ perception of the advantage of implementing calorie posting compared to the status quo or an alternative.  Inclusion Criteria: Include statements that demonstrate calorie posting is better (or worse) than the status quo or an alternative intervention.  Exclusion Criteria: Exclude statements that do or do not demonstrate a strong need for calorie posting and/or that the current situation is untenable (e.g. statements that calorie posting is absolutely necessary or that calorie posting is redundant with other interventions) and code to ‘[Tension for Change](http://cfirwiki.net/wiki/index.php?title=Tension_for_Change)’. |
| 1. Adaptability | Definition: Perceived inherent ability/allowance to adapt the calorie posting policy.  Inclusion Criteria: Include statements related to perceptions of the (in)ability to adapt the calorie posting policy.  Exclusion Criteria: Exclude statements regarding actual or suggested adaptations to the policy to meet local needs and code to the construct ‘Adapting the Policy’ under the CFIR ‘Process’ domain. |
| 1. Trialability | Definition: Perceived inherent ability/allowance to trial calorie posting on a small scale, and to be able to reverse course (undo implementation) if warranted.  Inclusion Criteria: Include statements related to perceptions of the (in)ability to trial (i.e. test/pilot) calorie posting.  Exclusion Criteria: Exclude statements regarding actual or suggested trials of calorie posting and code to the construct ‘Trialing’ under the CFIR ‘Process’ domain. |
| 1. Complexity | Definition: Perceived difficulty of calorie posting, reflected by duration, scope, radicalness, disruptiveness, centrality, and intricacy and number of steps required to implement.  Inclusion Criteria: Code statements regarding the complexity of calorie posting, which are not dependent on context.  Exclusion Criteria: Exclude statements which refer to factors operating within the internal setting of the hospital which effect implementation (e.g. lack of standardised recipes, lack of time) and code to relevant construct under the ‘Inner Setting’ domain. |
| 1. Design Quality & Packaging | Definition: Perceived quality of policy packaging and presentation, including materials and supports available.  Inclusion Criteria: Include statements regarding the quality of policy packaging and presentation, including materials and supports available.  Exclusion Criteria: Exclude statements regarding the receipt of materials as an engagement strategy and code to ‘[Engaging](http://cfirwiki.net/wiki/index.php?title=Engaging)’. |
| 1. Cost | Definition: Costs of the calorie posting policy and costs associated with implementing the policy.  Inclusion Criteria: Include statements related to the cost of the calorie posting policy and its implementation.  Exclusion Criteria: Exclude statements which refer to the presence or absence of resources in the internal setting of the hospital and code to ‘Available Resources’ (e.g. money, time). |
| **Outer Setting** | **The external environment to the hospital** |
| 1. Consumer Needs & Resources (OS) | Definition: The extent to which the needs and preferences of consumers (external to the hospital), as well as barriers and facilitators to meet those needs, are accurately known and prioritized by the hospital policy implementers.  Note: Consumers (OS) refers to consumers external to the hospital e.g. general public; whereas, consumers (IS) refers to consumers internal to the hospital i.e. staff utilising the canteen.  Inclusion Criteria: Include statements demonstrating (lack of) awareness of consumer needs, preferences and resources (e.g. consumer demand for calorie posting, barriers and facilitators of consumer participation in calorie posting).  Exclusion Criteria: Exclude statements regarding consumers internal to the hospital i.e. staff utilising the canteen and code to ‘Consumer Needs & Resources (IS)’. |
| 1. Cosmopolitanism | Definition: The degree to which the hospital is networked with other external organisations (i.e. external people and groups).  Inclusion Criteria: Include descriptions of outside group memberships and networking done outside the hospital, which are independent of calorie posting.  Exclusion Criteria: Exclude statements of networking with external organisations that did not exist prior to calorie posting and code to ‘Engaging’ construct. Exclude statements about general networking, communication, and relationships in the hospital, which are independent of calorie posting and code to ‘[Networks & Communications](http://cfirwiki.net/wiki/index.php?title=Networks_%26_Communications)’. |
| 1. Peer Pressure | Definition: Mimetic or competitive pressure to implement the calorie posting policy; typically, because most or other key peer or competing hospitals have already implemented or are in a bid for a competitive edge.  Inclusion Criteria: Include statements about perceived pressure or motivation from other hospitals to implement the calorie posting policy. |
| 1. Media & Societal Pressure* | Definition: Perceived pressure from media and societal expectations/watch-dogging.  Inclusion Criteria: Include statements about perceived pressure or motivation from media or society (i.e. mass media, the community, the public).  Exclusion Criteria: Exclude statements about perceived pressure or motivation from other hospitals and code to ‘Peer Pressure’. |
| 1. External Policy & Incentives | Definition: A broad construct that includes external strategies to spread calorie posting, including policy and regulations (governmental or other central entity).  Inclusion Criteria: Include descriptions of external strategies (outside the hospital) to spread calorie posting (e.g. policies, regulations, monitoring, awards).  Double code ‘Engaging: External Key Stakeholders’ or ‘Engaging: External Change Agents’ were either individuals have roles in the following, which influences implementation: external policy provision and/or targets, monitoring or enforcement, providing incentives such as awards. |
| 1. Economic Climate* | Definition: The extent to which the economic climate is known and prioritised by the hospital.  Inclusion Criteria: Include statements demonstrating awareness of the economic climate and its influence on implementation of calorie posting.  Exclusion Criteria: Exclude statements which refer to the presence or absence of resources in the internal setting of the hospital and code to ‘Available Resources’ (e.g. money). |
| 1. Educational System* | Definition: A broad construct which refers to the educational system.  Inclusion Criteria: Include statements about the educational system and its influence on implementation of calorie posting.  Exclusion Criteria: Exclude statements which refer to access to knowledge and information about calorie posting within the hospital and code to ‘[Access to Knowledge & Information](http://cfirwiki.net/wiki/index.php?title=Access_to_Knowledge_%26_Information)’. |
| 1. Culture (OS) ** | Definition: Norms, values, and basic assumptions which exist external to the hospital.  Note: Culture (OS) refers to a culture which exists external to hospital; whereas, culture (IS) refers to a culture internal to the hospital.  Inclusion Criteria: Include statements regarding norms, values, and basic assumptions which exist external to the hospital e.g. chef culture – individual cooking styles.  Exclusion Criteria: Exclude statements regarding a culture internal to the hospital and code to ‘Culture (IS)’. |
| **Inner Setting** | **The internal environment of the hospital** |
| 1. Structural Characteristics | Definition: The social architecture, age, maturity, and size of the hospital.  Inclusion Criteria: Include statements about the size, age, maturity and degree of specialisation of the hospital. Furthermore, include statements on decision-making autonomy, staff turnover and ratio of managers to total employees in the hospital.  Note: Include statements which refer to hospital characteristics which are independent of calorie posting – such as existing resources in hospital (not specific to implementation) e.g. staffing levels, under-resourced departments.  Exclusion Criteria: Exclude statements regarding resources specific to implementation and code to ‘Available Resources’. |
| 1. Networks & Communications | Definition: The nature and quality of webs of social networks and the nature and quality of formal and informal communications within the hospital.  Inclusion Criteria: Include statements about general networking, communication, and relationships in the hospital (such as descriptions of meetings, email groups, or other methods of keeping people connected and informed) and statements related to team formation, quality, and functioning – needs to be independent of calorie posting (i.e. exist prior to calorie posting).  Exclusion Criteria: Exclude statements about networking and communication that did not exist prior to calorie posting and code to ‘Engaging’ construct. Exclude statements related to implementation leaders' and staffs access to knowledge and information regarding using calorie posting and code to ‘[Access to Knowledge & Information](http://cfirwiki.net/wiki/index.php?title=Access_to_Knowledge_%26_Information)’. Exclude descriptions of outside group memberships and networking done outside the hospital, which are independent of calorie posting and code to ‘[Cosmopolitanism](http://cfirwiki.net/wiki/index.php?title=Cosmopolitanism)’. |
| 1. Culture (IS) | Definition: Norms, values, and basic assumptions of the hospital.  Note: Culture (OS) refers to a culture which exists external to hospital; whereas, culture (IS) refers to a culture internal to the hospital.  Inclusion Criteria: Include statements regarding the norms, values, and basic assumptions of the hospital e.g. resistance to change, traditional vs. progressive, patient vs. staff centred.  Exclusion Criteria: Exclude statements regarding a culture which exists external to the hospital and code to ‘Culture (OS)’. |
| 1. Consumer Needs & Resources (IS)** | Definition: The extent to which the needs and preferences of consumers (internal to the hospital), as well as barriers and facilitators to meet those needs, are accurately known and prioritized by the hospital policy implementers.  Note: Consumers (OS) refers to consumers external to the hospital e.g. general public; whereas, consumers (IS) refers to consumers internal to the hospital i.e. staff utilising the canteen.  Inclusion Criteria: Include statements demonstrating (lack of) awareness of consumer needs, preferences and resources (e.g. consumer demand for calorie posting, barriers and facilitators of consumer participation in calorie posting).  Exclusion Criteria: Exclude statements regarding consumers external to the hospital and code to ‘Consumer Needs & Resources (OS)’. |
| 1. Implementation Climate | Definition: The absorptive capacity for change, shared receptivity of involved individuals to the calorie posting policy, and the extent to which use of that policy will be rewarded, supported, and expected within their hospital.  Inclusion Criteria: Include statements regarding the general level of receptivity to implementing calorie posting.  Exclusion Criteria: Exclude statements regarding the general level of receptivity that are captured in the sub-codes below. |
| 1. Tension for Change | Definition: The degree to which stakeholders perceive the current situation as intolerable or needing change.  Inclusion Criteria: Include statements that (do not) demonstrate a strong need for calorie posting and/or that the current situation is untenable (e.g. statements that calorie posting is absolutely necessary or that it is redundant with other interventions/policies).  Exclusion Criteria: Exclude statements that demonstrate calorie posting is better (or worse) than existing interventions/policies and code to ‘[Relative Advantage](http://cfirwiki.net/wiki/index.php?title=Relative_Advantage)’. |
| 1. Compatibility | Definition: The degree of tangible fit between meaning and values attached to calorie posting by involved individuals, how those align with individuals’ own norms, values, and perceived risks and needs, and how calorie posting fits with existing workflows and systems.  Inclusion Criteria: Include statements that demonstrate the level of compatibility calorie posting has with the hospital mission/purpose/values and ways of working. |
| 1. Relative Priority | Definition: Individuals’ shared perception of the importance of implementing calorie posting within the hospital.  Inclusion Criteria: Include statements that reflect the relative priority of calorie posting (e.g. statements related to change fatigue in the hospital due to implementation of many other interventions/policies). |
| 1. Hospital Incentives & Rewards | Definition: Extrinsic incentives such as goal-sharing awards, performance reviews, promotions, and raises in salary, and less tangible incentives such as increased stature or respect.  Inclusion Criteria: Include statements related to whether incentive systems are in place to foster (or hinder) implementation, e.g. rewards or disincentives for staff engaging in calorie posting. |
| 1. Goals & Feedback | Definition: The degree to which goals are clearly communicated, acted upon, and fed back to staff, and alignment of that feedback with goals.  Inclusion Criteria: Include statements related to the (lack of) alignment of calorie posting with larger goals of the hospital, as well as feedback to staff regarding those goals.  Note: ‘Goals and Feedback’ is about two things:   1. The extent to which a hospital is data driven, i.e., do they set goals and update staff on progress? This part of the construct is independent of the intervention being implemented; it’s a characteristic of the context generally. 2. The extent to which the goals of the innovation are in line with existing facility goals. This part of the construct is tied to the innovation.   Double code ‘Reflecting and Evaluating’ where there is evidence of integration of evaluation components used as part of ‘Reflecting and Evaluating’ into on-going or sustained hospital structures and processes.  Exclusion Criteria: Exclude statements that refer to the process used in implementation, i.e., the implementation team’s (lack of) on-going review of implementation progress, that are not integrated into on-going or sustained hospital structures and processes, and code to ‘Reflecting & Evaluating’. |
| 1. Learning Climate | Definition: A climate in which: a) leaders express their own fallibility and need for team members’ assistance and input; b) team members feel that they are essential, valued, and knowledgeable partners in the change process; c) individuals feel psychologically safe to try new methods; and d) there is sufficient time and space for reflective thinking and evaluation.  Inclusion Criteria: Include statements that support (or refute) the degree to which key components of the hospital exhibit a ‘learning climate’. |
| 1. Readiness for Implementation | Definition: Tangible and immediate indicators of hospital commitment to its decision to implement calorie posting.  Inclusion Criteria: Include statements regarding the general level of readiness for implementation.  Exclusion Criteria: Exclude statements regarding the general level of readiness for implementation that are captured in the sub-codes below. |
| 1. Leadership Support | Definition: Commitment, involvement, and accountability of leaders and managers with the implementation.  Inclusion Criteria: Include statements regarding the level of commitment/support of leadership in the hospital.  Double code statements regarding leadership commitment/support to ‘Engaging: Opinion Leader’, ‘Engaging: [Formally Appointed Internal Implementation Leaders](http://cfirwiki.net/wiki/index.php?title=Formally_Appointed_Internal_Implementation_Leaders)’, ‘Engaging: Champion’ or ‘Engaging: Internal Key Stakeholders’ where applicable. |
| 1. Available Resources | Definition: The level of resources dedicated for implementation and on-going operations, including time, money, space, equipment, etc.  Inclusion Criteria: Include statements related to the presence or absence of resources described above or resources specific to calorie posting implementation.  Exclusion Criteria: Exclude statements related to hospital resources independent of calorie posting and code to ‘Structural Characteristics’. Exclude statements related to training and education and code to ‘[Access to Knowledge & Information](http://cfirwiki.net/wiki/index.php?title=Access_to_Knowledge_%26_Information)’. |
| 1. Access to Knowledge & Information | Definition: Ease of access to digestible information and knowledge about calorie posting and how to incorporate it into work tasks.  Note: Information and knowledge includes all sources such as experts, other experienced staff, training, documentation, and computerized information systems.  Inclusion Criteria: Include statements related to stakeholder access to knowledge and information regarding the calorie posting policy. Include knowledge and information available from within the implementing hospital (e.g. training provided by staff) or those available from external entities/stakeholders to the hospital (e.g. via Irish Heart Foundation, students or HSE providing information).  Exclusion Criteria: Exclude statements about general networking, communication, and relationships in the hospital, which are independent of the calorie posting policy and code to ‘[Networks & Communications](http://cfirwiki.net/wiki/index.php?title=Networks_%26_Communications)’. Exclude statements about the general skill set or knowledge of a particular stakeholder involved in implementation and code to relevant ‘Engaging’ sub-construct. |
| **Characteristics of Individuals** | **Characteristics of the individuals involved in implementation**  Code information about individuals to their relevant role code under ‘Engaging’. |
| **Process** | **Process involved in the implementation calorie posting** |
| 1. Planning | Definition: The degree to which a scheme or sequence of tasks for implementing calorie posting are developed in advance, and the quality of those schemes or tasks.  Inclusion Criteria: Include evidence of pre-implementation diagnostic assessments and planning, as well as refinements to the plan. |
| 1. Engaging | Definition: Attracting and involving appropriate individuals in the implementation and use of calorie posting.  Inclusion Criteria: Include statements related to engagement strategies and outcomes (i.e. if and how stakeholders became engaged with calorie posting and what their role is in implementation). Although both strategies and outcomes are coded here, the outcome of engagement efforts determines the rating, i.e., if there are repeated attempts to engage a stakeholder that are not successful, or if a role is vacant, the construct receives a negative rating (i.e. a barrier). In addition, code the “quality” of stakeholders – their capabilities, motivation, and skills, i.e., how good they are at their job, and this affects the rating as well.  Note: The engaging process can be bi-directional and to code statements related to outcomes of this process (which stakeholder is engaged or not engaged and their involvement).  Exclusion Criteria: Exclude statements that are captured in the sub-codes below. |
| 1. Opinion Leaders | Definition: Individuals in the hospital who have formal or informal influence on the attitudes and beliefs of their colleagues with respect to implementing calorie posting.  Note: There is general agreement that there are two different types of opinion leaders, experts and peers. Expert opinion leaders exert influence through their authority and status. Peer opinion leaders exert influence through their representativeness and credibility.  Inclusion Criteria: Include statements related to engagement strategies and outcomes (e.g. how the opinion leader became engaged with calorie posting and what their role is in implementation). Although both strategies and outcomes are coded here, the outcome of engagement efforts determines the rating, i.e. if there are repeated attempts to engage an opinion leader that are not successful, or if the opinion leader steps down from their role or leaves the hospital and this role is vacant, the construct receives a negative rating (i.e. a barrier). In addition, you may also want to code the “quality” of the opinion leader here – their capabilities, motivation, and skills, i.e. how good they are at their job, and this affects the rating as well.  Double code statements to ‘Leadership Support’ (under the ‘Inner Setting’ domain) if the opinion leader is also a hospital leader/manager. |
| 1. Formally Appointed Internal Implementation Leaders | Definition: Individuals from within the hospital who have been formally appointed with responsibility for implementing calorie posting as coordinator, project manager, team leader, or other similar role.  Inclusion Criteria: Include statements related to engagement strategies and outcomes (e.g. how the formally appointed internal implementation leader became engaged with calorie posting and what their role is in implementation). Although both strategies and outcomes are coded here, the outcome of engagement efforts determines the rating, i.e. if there are repeated attempts to engage an implementation leader that are not successful, or if the implementation leader steps down from their role or leaves the hospital and this role is vacant, the construct receives a negative rating (i.e. a barrier). In addition, you may also want to code the “quality” of the implementation leader here – their capabilities, motivation, and skills, i.e. how good they are at their job, and this affects the rating as well.  Double code statements to ‘Leadership Support’ (under the ‘Inner Setting’ domain) if the formally appointed internal implementation leader is also a hospital leader/manager. |
| 1. Champions | Definition: Individuals who dedicate themselves to supporting, marketing, and ‘driving through’ an implementation, overcoming indifference or resistance that a calorie posting policy may provoke in a hospital.  Inclusion Criteria: Include statements related to engagement strategies and outcomes (e.g. how the champion became engaged with calorie posting and what their role is in implementation). Although both strategies and outcomes are coded here, the outcome of engagement efforts determines the rating, i.e. if there are repeated attempts to engage a champion that are not successful, or if the champion steps down from their role or leaves the hospital and this role is vacant, the construct receives a negative rating (i.e. a barrier). In addition, you may also want to code the “quality” of the champion here – their capabilities, motivation, and skills, i.e., how good they are at their job, and this affects the rating as well.  Double code statements to ‘Leadership Support’ (under the ‘Inner Setting’ domain) if the champion is also a hospital leader/manager. |
| 1. External Change Agents | Definition: Individuals who are affiliated with an outside entity (related or unrelated to the hospital) who formally influence or facilitate calorie posting in a desirable direction.  External change agents may include researchers, students on placement and a health agency external to the hospital.  Inclusion Criteria: Include statements related to engagement strategies and outcomes (e.g. how the external change agent became engaged with calorie posting and what their role is in implementation (e.g. how they supported implementation efforts). Although both strategies and outcomes are coded here, the outcome of engagement efforts determines the rating, i.e. if there are repeated attempts to engage an external change agent that are not successful, or if the external change agent role becomes vacant, the construct receives a negative rating (i.e. a barrier). In addition, you may also want to code the “quality” of the external change agent here – their capabilities, motivation, and skills, i.e. how good they are at their job, and this affects the rating as well.  Double code ‘External Policy & Incentives’ were external change agents (e.g. researcher, Irish Heart Foundation) have a role in the following, which influences implementation: monitoring or enforcement, and/or providing incentives such as awards. |
| 1. Internal Key Stakeholders* | Definition: Individuals from within the hospital with responsibility for implementing calorie posting.  Note: Internal key stakeholders may include direct (catering staff) and indirect (health care professionals, hospital management) stakeholders within the hospital with responsibility for implementing calorie posting.  Inclusion Criteria: Include statements related to engagement strategies and outcomes, e.g., how internal key stakeholders became engaged with calorie posting and what their role is in implementation. Although both strategies and outcomes are coded here, the outcome of engagement efforts determines the rating, i.e. if there are repeated attempts to engage an internal key stakeholder that are not successful, or if the internal key stakeholder steps down from their role or leaves the hospital and this role is vacant, the construct receives a negative rating (i.e. a barrier). In addition, you may also want to code the “quality” of the internal key stakeholder here – their capabilities, motivation, and skills, i.e. how good they are at their job, and this affects the rating as well.  Double code statements to ‘Leadership Support’ (under the ‘Inner Setting’ domain) if the internal key stakeholder is also a hospital leader/manager.  Exclusion Criteria: Exclude statements related to internal stakeholders who act as an opinion leader and code ‘Engaging: Opinion Leaders’. Exclude statements related to internal stakeholders who act as a champion for calorie posting and code ‘Engaging: Champions’. Exclude statements related to internal stakeholders who have been formally appointed as implementation leaders and code to ‘Engaging: Formally Appointed Internal Implementation Leaders’. |
| 1. External Key Stakeholders* | Definition: Individuals from outside the hospital with an indirect role in implementing calorie posting.  Note: External key stakeholder may include food suppliers/purveyors.  Inclusion Criteria: Include statements related to engagement strategies and outcomes, e.g., how external key stakeholders became engaged with calorie posting and what their role is in implementation. Although both strategies and outcomes are coded here, the outcome of engagement efforts determines the rating, i.e. if there are repeated attempts to engage an external key stakeholder that are not successful, or if the external key stakeholder role becomes vacant, the construct receives a negative rating (i.e. a barrier). In addition, you may also want to code the “quality” of the external key stakeholder here – their capabilities, motivation, and skills, i.e. how good they are at their job, and this affects the rating as well.  Double code ‘External Policy & Incentives’ were external key stakeholders have a role in the following, which influences implementation: external policy provision and/or targets, monitoring or enforcement and/or providing incentives such as awards  Exclusion Criteria: Exclude statements related to external change agents and code to ‘External Change Agents’. |
| 1. Consumers (IS)* | Definition: Engagement of staff utilising the canteen (i.e. consumers).  Inclusion Criteria: Include statements related to engagement strategies and outcomes, e.g., how consumers became engaged with calorie posting. Although both strategies and outcomes are coded here, the outcome of efforts to engage consumers determines the rating, i.e., if there are repeated attempts to engage consumers that are not successful, the construct receives a negative rating (i.e. a barrier).  Exclusion Criteria: Exclude statements demonstrating (lack of) awareness of the needs and resources of consumers internal to the hospital and code to ‘[Consumer Needs & Resources](https://cfirguide.org/constructs/patient-needs-and-resources/) (IS)’. Exclude statements demonstrating (lack of) awareness of the needs and resources of consumers external to the hospital and code to ‘[Consumer Needs & Resources](https://cfirguide.org/constructs/patient-needs-and-resources/) (OS)’. |
| 1. Executing | Definition: Carrying out or accomplishing the implementation according to plan.  Inclusion Criteria: Include statements that demonstrate how implementation occurred with respect to the implementation plan.  Note: Fidelity measures can be used to assess executing, as an indication of the degree to which implementation was accomplished according to plan. |
| 1. Reflecting & Evaluating | Definition: Quantitative and qualitative feedback about the progress and quality of implementation accompanied with regular personal and team debriefing about progress and experience.  Inclusion Criteria: Include statements that refer to the implementation team’s (lack of) assessment of the progress toward and/or impact of implementation.  Note: ‘Reflecting and Evaluating’ (RE) is specific to the process of implementation; the extent to which they are monitoring progress/impact of implementation. This construct can be spilt into RE IMP and RE INT, i.e., RE around implementation and RE around the effectiveness of calorie posting. RE-INT is a process to assess how it’s working in a hospital.  Evidence of the integration of evaluation components used as part of ‘Reflecting and Evaluating’ into on-going or sustained hospital structures and processes may be double coded to ‘Goals and Feedback’.  Exclusion Criteria: Exclude statements related to the (lack of) alignment of calorie posting goals with larger hospital goals, as well as feedback to staff regarding those goals and code to ‘[Goals & Feedback](http://cfirwiki.net/wiki/index.php?title=Goals_%26_Feedback)’. Exclude statements related to whether the calorie posting policy had an evidence based (before coming into the current hospital) and code to ‘Evidence Strength & Quality’. |
| 1. Adapting the Organisation* | Definition: Activities which are related to refining existing workflows to accommodate the calorie posting policy (i.e. adapting the hospital/canteen).  Inclusion Criteria: Include statements which describe activities to refine existing work practices to accommodate calorie posting. |
| 1. Adapting the Intervention* | Definition: Actual or suggested adaptations to the calorie posting policy to meet local needs.  Inclusion Criteria: Include statements which describe actual or suggested adaptations to the calorie posting policy to meet local needs.  Exclusion Criteria: Exclude statements related to perceptions of the (in)ability to adapt the calorie posting policy and code to ‘Adaptability’. |
| 1. Trailing* | Definition: Actual or suggested trials of calorie posting.  Inclusion Criteria: Include statements which describe actual or suggested trials (i.e. testing/piloting) of calorie posting.  Exclusion Criteria: Exclude statements related to perceptions of the (in)ability to trial calorie posting and code to ‘Trialability’. |
| 1. Scaling Up* | Definition: Activities to assist with scaling up of calorie posting.  Inclusion Criteria: Include statements which describe activities undertaken to scale up calorie posting within the hospital. |
| 1. Strategy** | Definition: Strategies to implement calorie posting, outside of what is captured in other constructs within this domain.  Inclusion Criteria: Include statements which describe strategies used to assist with implementation (not captured in other constructs within this domain).  Exclusion Criteria: Exclude statements which describe strategies captured in other constructs within this domain. |

**Symbols: * = new construct generated inductively from recent systematic review [**[**1**](#_ENREF_1)**], ** = new construct generated inductively from the data.**

**Abbreviations: IS = inner setting, OS = outer setting.**

**Reference**

1. Kerins C, McHugh S, McSharry J, Reardon CM, Hayes C, Perry IJ, et al. Barriers and facilitators to implementation of menu labelling interventions from a food service industry perspective: a mixed methods systematic review. ‎Int J Behav Nutr Phys Act. 2020;17:48.
