# Supplementary material for: Factors Influencing Fidelity to a Calorie Posting Policy in Public Hospitals: A Mixed Methods Study
Source: Front Public Health. 2021 Aug 13;9:707668. doi: 10.3389/fpubh.2021.707668 (PMC8414889; doi:10.3389/fpubh.2021.707668)
Supplement: Supplementary file 3 [file Table_3.DOCX]

| **Additional File 3. List of constructs and sample quotes following the deductive and inductive coding** | | | | |
| --- | --- | --- | --- | --- |
| **CFIR Domain** | **Barriers of Implementation** | **Facilitators to Implementation** | **Future Recommendations** | **Sample Quotes** |
| **Intervention Characteristics** |  |  |  |  |
| Intervention Source | No data | No data | No data |  |
| Evidence Strength & Quality | No data | No data | No data |  |
| Relative Advantage | Loss of business (sales down, external caterer take over canteen) [Hospital 2] | Makes good customer and business sense [Hospital 4] | Need to see benefits [Hospital 1] | *In one way I suppose another disincentive purely from the business end of it was you know the more you were calorie posting the contents of some of the items they were losing business. You know the scones were dropping. The sausage rolls were dropping… So rather than having their business drop off a cliff altogether it was easier not to calorie post and keep selling the more expensive or the less nutritious items.* [Hospital 2 – barrier]  *If it makes sense and it makes good customer… or business sense you know… we will do it.* [Hospital 2 – facilitator]  *You know people need to see what’s the value. You know how does this add value to their organisation or to their department or to their staff. What’s in it for them locally, you know the benefits of it.* [Hospital 1 – future facilitator] |
| Adaptability | No data | No data | No data |  |
| Trialability | No data | No data | No data |  |
| Complexity | Large volume of work required [Hospital 1,2,3,4]  High level of precision required [Hospital 1,2,3,4]  Implementation a time consuming process [Hospital 1,3,4] | No data | No data | *We could do up to 35 portions of fresh fruit salad every day and it depends on what’s in it. Grapes, mandarins, whatever. And the calorie counts like there could be four grapes in this one, there could be two in this one because you’re spooning them up so fast and putting the lid on them. You’re not going to be saying one grape for you, one grape for you, one grape for you.* [Hospital 2 - barrier]  *The scones and the brown bread we find are the hardest to have a standard thing... A slice of brown bread can vary from about 65 grams to 85 grams. The brown bread if it goes in the tin and it rises or it doesn't… We changed all the tins to loaf tins and they're all the same size. But it’s still an issue.* [Hospital 3 - barrier]  *You know so it takes an awful lot of work just to do one dish you know… It’s so time-consuming*. [Hospital 4 - barrier] |
| Design Quality & Packaging | Lack of supporting materials [Hospital 1,2,3,4]  Non-user friendly for customers [Hospital 1,2,3,4]  Impractical display option [Hospital 1,2,4]  Lack of (clear) guidance on best practice [Hospital 2,3]  Lack of guidance on auditing the policy [Hospital 4] | Policy supporting materials [Hospital 1] | No data | *There was no supporting information to help us. We were all kind of working at it ourselves through the internet… It was up to yourself to get on with it.* [Hospital 2 – barrier]  *Just even recommendations or what you have to actually have in place for the calorie posting. It’s like we knew we had to have the nutritional analysis done and we were getting the energy content and the calories up for that. So therefore you know we just put them up. Whether we put them up per ounce or per portion I think it was just down to the student kind of feeling this was the most appropriate way to put it out there.* [Hospital 3 – barrier]  *Ordered clear perspex signs to display calories…the heat of the lights with the hot food was melting them. You know it was very difficult to find appropriate display merchandise.* [Hospital 2 – barrier]  *I remember there was the implementation plan, there was the policy and then there was the check list… Oh it was very helpful.* [Hospital 1 – facilitator] |
| Cost | Cost of releasing staff from work [Hospital 2,4]  Cost of purchasing nutrition analysis software [Hospital 1] | No data | No data | *You’re looking at you know for a five year licence you’re probably looking at something like five grand or more. You know it’s a good bit of money…* [Hospital 1 – barrier]  *If staff need to be released and their role backfilled… it does have cost implications for the hospital.* [Hospital 2 – barrier] |
| **Outer Setting** |  |  |  |  |
| Consumer Needs & Resources (OS) | No data | Improving consumer health [Hospital 1,2,4] | No data | *But when you look at the bigger picture of Ireland as a nation I suppose and where we are in terms of obesity and all that kind of stuff you can say well all this makes sense.* [Hospital 4 – facilitator] |
| Cosmopolitanism | Hospitals working in silos [Hospital 1,2]  Lack of communication or teamwork between hospital canteens [Hospital 1] | Hospital group ‘Healthy Ireland Implementation Plan’ (includes nutrition KPIs) [Hospital 1,3]  Reporting on implementation progress to hospital group [Hospital 3]  HSE Policy Group [Hospital 1] | Communication and teamwork between hospital canteens [Hospital 1]  Develop relationship with universities [Hospital 4] | *There’s another hospital up the road… I don’t think the ladies working here 30/40 years have ever spoke to somebody in another place like, do you know what I mean… Every hospital and canteen sticks to itself… no sharing of recipes, nutritional information…* [Hospital 1 - barrier]  *There was also a Healthy Ireland Implementation Plan and the [hospital group name], they would have devised their own Healthy Ireland Implementation Plan 2015/2017 and included in that there was a nutrition section… there was some good nutrition KPIs in that and they included the analysis of the hospital food for patients in terms of nutrient content and also portion sizes but also included by the implementation of the calorie posting… So for every hospital in the group this was one of the nutrition KPIs you had to deliver on. So I think it got management support at a high level or at least on paper it did.* [Hospital 1 - facilitator]  *Well as I said before I do think communication would probably be the most important… If hospital canteens worked together it would be so much easier...* [Hospital 1 – future facilitator] |
| Peer Pressure | No data | Gaining a competitive edge over other hospitals [Hospital 1,3]  Pressure to keep up with other hospitals [Hospital 2,3] | No data | *I think we were one of the first hospitals to get the golden healthy heart which we are very proud of so there was that little bit of competition because we got it very early. We got it before anybody else in the group. So we were quite proud of that.* [Hospital 1 – facilitator]  *Yeah, we like to be top of the class* [Hospital 3 - facilitator] |
| External Policy & Incentives | Lack of (ongoing, rigorous/comprehensive) monitoring [Hospital 2,3,4]  Lack of menu labelling legislation [Hospital 2,3]  No external incentives or recognition [Hospital 1,4]  Less liability risk with calories (compared with allergens) [Hospital 2]  Union causing delays with recruitment (bureaucracy) [Hospital 2] | (Impending) menu labelling legislation [Hospital 1,2,3,4]  HSE national policy (and standards) [Hospital 1,2,3,4]  Irish Heart Foundation award [Hospital 1,2,3,4]  External monitoring [Hospital 2,3,4]  Healthy Ireland (a Government-led initiative) [Hospital 1,2,3]  Operation Transformation (popular health and fitness programme airing on Irish TV) [Hospital 1,4]  HSE national award [Hospital 1]  National Obesity Strategy in Ireland [Hospital 1]  National Workplace Wellness Day [Hospital 2]  Nutrition & Hydration Week (global movement) [Hospital 2]  Pressure from government [Hospital 2] | External monitoring (ongoing - bi-yearly audits and unannounced, rigorous/ comprehensive - kitchen and service area) [Hospital 1,2,3,4]  External incentives and recognition [Hospital 1,4]  HSE national award (external quality assurance award - status symbol - standards that you have to meet) [Hospital 1]  Irish Heart Foundation award [Hospital 1]  KPI for calorie posting [Hospital 2] | *There was no checking in what you do with the canteen but there was red marks and there was people checking up in terms of are you meeting the HIQA nutrition patient calorie requirements.* [Hospital 1 - barrier]  *Audits are not done correctly and thoroughly… that’s why these errors are not picked up.* [Hospital 4 - barrier]  *Oh it’s something about the allergens. It’s a legal requirement. It’s a legislative requirement whereas calorie posting wasn’t.* [Hospital 2 - barrier]  *So you want to be at least thinking the same way that policy makers are thinking… So if you know that stuff is in draft form you say well its draft form, its gonna happen in a couple of years, it might be four or five, let’s just forge ahead.* [Hospital 4 - facilitator]  *They sent out the email to say that it was going to be legislation and that was really what drove it.* [Hospital 3 - facilitator]  *I think probably the main driver would have been Healthy Ireland… Well healthy Ireland was seen as an agenda that the hospital had to do, you had to get on board with it as part of the hospital groups things. So I could get things through under Healthy Ireland that people might have been trying to get through before that they couldn’t. So it was a definite supporter.* [Hospital 3 - facilitator]  *It should be something possibly that’s double checked or every so often spot checked… an external audit is a great stimulus.* [Hospital 3 – future facilitator]  *I would recommend that they need at least yearly, if not bi-yearly audits and unannounced. That’s the way I would do it.* [Hospital 4 – future facilitator] |
| Economic Climate* | Recession [Hospital 1] | No data | No data | *And we’ve gone through you know obviously the situation in the country with all the cutbacks…* [Hospital 1 – barrier] |
| Educational System* | Learning different cooking methods [Hospital 2] | More education on calorie posting in schools [Hospital 4] | No data | *Chefs being taught different ways of doing this…* [Hospital 2 – barrier]  *And what I will say to you is I am getting better as every year goes on. Because the kids that are coming in as well teach me. I teach them an element but they teach me as well because every year I find with the schools they seem to be learning more and more about it.* [Hospital 4 – facilitator] |
| Media & Societal Pressure* | No data | No data | Progress reports published publicly [Hospital 1] | *What would help is publishing a report that’s available publically for all to see that is critical or otherwise of your hospital.* [Hospital 1 – future facilitator] |
| Culture (OS)** | Chef culture – individual cooking styles [Hospital 2,4] | No data | No data | *So we looked at trying to standardise the recipes and that was quite difficult really in that… they knew the recipes off the top of their heads so they weren’t really using recipes per say. You know they just knew how to make different things.* [Hospital 2 – barrier]  *Chefs maybe more naturally have kind of a creativity… They’re not used to following a set of rules for a recipe shall we say.* [Hospital 4 – barrier] |
| **Inner Setting** |  |  |  |  |
| Structural Characteristics | Short staffed (due to maternity leaves not filled, high absenteeism, recent retirements, staff on leave or out sick, vacancies, recruitment delays) [Hospital 1,2,3,4]  Under-resourced catering and dietetic departments (1,2,4)  Lack of assistant catering manager [Hospital 1,2]  No dedicated position or person for implementation of such policies [Hospital 3,4]  Long term staff [Hospital 2,4]  Voluntary hospital (not HSE owned) [Hospital 1,4]  Large hospital (or getting bigger) [Hospital 2,4]  Kitchen not fit for purpose or outdated [Hospital 1,2]  Centralised decision making [Hospital 2,4]  Changes to canteen layout [Hospital 1]  Complex patient type (requiring more dietetic and catering input) [Hospital 2]  Exceeding bed capacity [Hospital 2]  No food services dietitian [Hospital 4]  No health promotion department [Hospital 1]  No service/facilities manager [Hospital 1]  Short work contracts [Hospital 1]  Small rural hospital (difficult to get staff) [Hospital 1] | Long term staff [Hospital 1,3]  Designated chef for staff canteen [Hospital 4]  Hospital foundation (charity raising funds to support hospital) [Hospital 4]  Newly renovated canteen (not kitchen) [Hospital 2]  Small hospital (were staff know each other) [Hospital 1]  Hospital staff profile (mostly female and younger age) [Hospital 1] | Create a dedicated position or allocate a person for implementation of such policies [Hospital 1,2,3,4]  Food services dietitian to assist with implementation [Hospital 1,2,4]  Adequate staffing levels [Hospital 1,4]  Adequate middle management in catering department [Hospital 2] | *There have been issues with resourcing in the catering department. Obviously there have been resourcing issues in our department as well at times. Particularly I suppose it’s been maternity vacancies... I’ve two maternity vacancies this year. As long as they’re backfilled you know. Otherwise we’re in total crisis situation. But most certainly if we were down 50% staff we have absolutely no time to look at anything for staff. Patients only you know.* [Hospital 1 – barrier]  *Massive issues with lack of management resources. We’ve plenty of staff on the ground in terms of catering staff, catering assistants and chefs. The issue is management of staff… And then last June I was given control at ward level which is another 100 whole time equivalent staff. I think it’s 126 on the roster but between parental days and days off its 100 WTE. And I was given three supervisors. So on a bad day I could have one supervisor. On a good day two supervisors to manage 100 WTE.* [Hospital 2 - barrier]  *Implementing it in a very large institution can be very difficult… Just purely because of the large numbers and the volume…* [Hospital 4 – barrier]  *I mean we’re not a massive site if you know what I’m trying to say and everyone knows everybody. The people that were part of the group new how to network the system… It’s very easy to network and communicate very quickly.* [Hospital 1 – facilitator]  *You think that it would facilitate the likes of implementing calorie posting and other staff policies if there was a dedicated member of staff in the food services, so whether it was the dietitian in that role or catering staff in that role.* [Hospital 2 – future facilitator]  *Other countries have catering dietitians and catering dietitian resources… we need them here too, to do these key pieces of work…* [Hospital 1 – future facilitator] |
| Networks & Communications | Lack of communication or teamwork between departments or stakeholders [Hospital 1,2,4]  No hospital committee meetings [Hospital 1,3] | Support from hospital committee or group [Hospital 1,2,3,4]  Good relationships (involving communication and teamwork) within or between departments and stakeholders [Hospital 1,4]  Easy to network and communicate [Hospital 1] | Communication between departments or stakeholders [Hospital 1]  Support from hospital committee or group [Hospital 1] | *So the communication bit I think is a weak point I think overall down there.* [Hospital 2 - barrier]  *I think there’s more needs to be done there. But at the time then I stepped down as the Healthy Ireland lead and the group haven’t met since. And that was last September. And the momentum got completely lost.* [Hospital 2 - barrier]  *The hospital committees definitely help. You’re dealing with and you’re engaging with likeminded people. You know like we’re all kind of cousins like dietetics and catering and speech and language. Even though we have different roles the focus is on patients or the focus is on staff. So it does kind of work…So there’s a linkage all the time but there’s about four or five departments and they’re strongly linked.* [Hospital 4 - facilitator]  *I don’t think this would have been done unless there was a good relationship between particularly the dietetics and the catering.* [Hospital 3 - facilitator] |
| Culture (IS) | Patient care the focus (at expense of staff health and wellbeing) [Hospital 1,2,3,4]  Resistance to change [Hospital 1,2,4]  Project orientated (do one thing, then move onto next) [Hospital 2,4]  Traditional culture (not progressive) [Hospital 1] | Staff-centred catering [Hospital 1,3,4]  Culture of improvement [Hospital 1,4]  Ethos of healthy living and wellbeing [Hospital 1,3] | No data | *I would say the patients would always take priority… patients come first. They will prioritise getting everything 100% for the patients but the staff canteen suffers as a result.* [Hospital 1 – barrier]  *Even if we’d had ones that cost €100 and they were all singing and dancing I still don’t know would the canteen staff have put them out. You know there was a resistance.* [Hospital 2 – barrier]  *There’s a culture within the catering department firstly where they just love to serve staff and they want to give them the nicest and the best food possible and present it in the best way… So this was an easy one really because we just knew it was going to build on a good tradition and a good sound belief that they could do better. And everyone bought into it.* [Hospital 1 - facilitator] |
| Consumer Needs & Resources (IS)** | Consumer needs and preferences regarding portions (males need more, consumers don’t want standardised portions but value for money) [Hospital 1,2,3,4]  Confused consumers (lack of understanding of calories) [Hospital 1,2,3,4]  Lack of consumer demand (especially males) [Hospital 1,2,3]  Not meeting consumer needs (preference for different type of intervention, consumers in rush) [Hospital 1,2,3]  Risk of misinforming consumers (with inaccurate calorie information) [Hospital 2] | Consumer demand (especially when first launched and greater demand amongst females) [Hospital 1,2,4]  Improving consumer health [1,2,4]  Enabling informed food choices [Hospital 2,4]  Meeting consumer needs (providing nutrition information and doing the right thing by consumers) [Hospital 1,4] | Consumer demand [Hospital 2,3] | *You’re doing all this work and then it’s just being ignored or you know there’s no uptake on it.* [Hospital 1 – barrier]  *They’re just going to take a scoop of coleslaw and it’s not going to be 3 ounces or 4 ounces or whatever it’s meant to be. It’s going to be quite high… And that’s more to do with getting value for their money.* [Hospital 3 – barrier]  *Well if you’re going to be doing it I feel you should be doing it right. And you’re misinforming people I think if you give one as I said two big slices and another one two small slices. I feel you’re misinforming them especially with that side of it.* [Hospital 2 – barrier]  *Look it’s all to do with giving our customers more information and it’s about providing information that might hopefully make people healthier and more aware of what they’re eating.* [Hospital 4 – facilitator]  *I think if there was more demand it would be implemented better.* [Hospital 3 – future facilitator] |
| Implementation Climate |  |  |  |  |
| Tension for Change | No tension for change (no appetite for change, don’t see need) [Hospital 1,2,4] | Tension for change (appetite for change, see the need, driven by other factors causing need) [Hospital 1,2,4] | Tension for change (catering department see need and have an interest) [Hospital 3,4] | *Whereas in the canteen itself some of the staff I encountered there’s a sense of why are we doing this at all.* [Hospital 2 – barrier]  *They saw the need for it in the context of seeing the need and they bought into it definitely.* [Hospital 1 – facilitator]  *My personal feeling would be the main drive for that would be in the catering department needs to have a massive appetite for you know healthy options, calorie posting, portion control going forward.* [Hospital 3 – future facilitator] |
| Compatibility | Changing ingredients or menu [Hospital 1,2,3,4]  Lack of standardised recipes or portions (for certain menu items) [Hospital 1,2,4]  Menu display signage changing through-out day [Hospital 2,4]  Home-made menu items (not bought-in or pre-packaged) [Hospital 3]  Several point-of-choice areas in canteen [Hospital 2,4]  Several vendors [Hospital 2] | Aligns with existing practices or initiatives (doing nutritional analysis of patient menus, same meals served to patients and staff, posting allergens, Irish Heart Foundation initiative etc.) [Hospital 1,2,3,4]  Bought-in or pre-packaged menu items (not home-made) [Hospital 1,2,4]  Sense of responsibility [Hospital 1,3]  No changes to menu [Hospital 1]  Standardised size products [Hospital 2] | No data | *For us to maintain it especially if you’re working where you’re changing menus regularly and new foods and new recipes and whatever you know there is extra resource requirement on an ongoing basis to maintain calorie posting in place. It’s not like you do it once and that’s it…* [Hospital 1 – barrier]  *Because our food didn’t change that much and our menus didn’t change that much it stayed… it was all very standardised… it was easy to keep it going.* [Hospital 1 – facilitator]  *She did it in conjunction with the analysis of our patient menus… Some of the food that’s available for the patient menus is the same as what’s served in the canteen. So a lot of the time while she was processing the patient menus she could just take that information and put it on to the calorie posting for the canteen.* [Hospital 3 – facilitator] |
| Relative Priority | Not a priority (patients, food safety, allergens and other initiatives more important) [Hospital 1,2,3,4]  Competing initiatives [Hospital 2,3,4]  Overwhelmed with yet another initiative or requirement [Hospital 1,2,3] | Serving healthy food a priority [Hospital 4] | Calorie posting seen as a priority (by hospital management) [Hospital 1,2]  Make serving healthy food for staff a priority [Hospital 1,3] | *I don’t think there was a huge amount of emphases on the calorie posting in the canteen before we had to do it for the patient menus. When it had to be done for the patient menus it was a big priority. Being done for the staff it was take it or leave it.* [Hospital 3 – barrier]  *It’s just when you’re in the cutting throat stuff, the day to day operation things catch up and it gets parked to the side.* [Hospital 4 – barrier]  *… the service of meals and nutritious meals to patients and to our staff would be important to us, it is kind of the driving force.* [Hospital 4 – facilitator]  *You know there’s been a lot of changes here with nutrition and hydration in relation to you know healthy snacks for patients and things being made available. And that is brilliant. And I think the same needs to apply to feeding the staff as to the patients.* [Hospital 3 – future facilitator] |
| Hospital Incentives & Rewards | No incentives [Hospital 2,4]  Lack of recognition [Hospital 2,3] | Recognition [Hospital 3] | More recognition [Hospital 2,3] | *There’s no incentive in it you know if you’re not given the time… like it is on top of what I’m already doing you know, something has to give.* [Hospital 4 – barrier]  *I mean like they put our picture in the paper when we won the happy heart award… Yeah hospital management would recognise our achievement.* [Hospital 3 – facilitator]  *Recognition is important… I think it would probably need to be more than a comment of well-done you know. It would definitely need to be more supportive than that.* [Hospital 3 – future facilitator] |
| Goals & Feedback | No data | In line with hospital goals [Hospital 1] | No data | *I suppose we were getting on the whole Healthy Ireland agenda. So it fell into I suppose from the very beginning of the Healthy Ireland establishment of that the catering department and nutrition was right up there at the top in terms of it probably was one of our main aims and objectives. It was in there as our main objective when we started out. Yeah so it coincided with that yeah so it was an easy one.* [Hospital 1 – facilitator] |
| Learning Climate | No data | Learning climate [Hospital 1,2,4] | No data | *They’re all looking and willing to learn and stuff like that. Since I came here now, and I’m only here a couple of weeks and they’re looking for positives and they’re looking for ways to be shown because everyone likes to learn something new you know.* [Hospital 1 – facilitator]  *We don’t look at those things as criticism. You know whether it’s a hygiene audit or whatever. You learn something from everyone that comes in.* [Hospital 4 – facilitator] |
| Readiness for Implementation |  |  |  |  |
| Leadership Support | Hospital management and leaders not engaged (not supportive of policy and implementation, vacant roles etc.) [Hospital 1,2,3,4]  (Assistant) catering manager not engaged (not supportive of policy and implementation, vacant role, lack of nutrition expertise to supervise students, lack of resources - so require students, new to job so unable to supervise students etc.) [Hospital 1,2,3,4]  Catering middle management (i.e. under assistant catering manager) not engaged (due to lack of buy-in, lack of nutrition expertise) [Hospital 4] | (Assistant) catering manager engaged (supportive of policy, involved in implementation, monitoring compliance, supervising students etc.) [Hospital 1,2,3,4]  Hospital management and leaders engaged (supportive of implementation, providing recognition etc.) [Hospital 1,2,3,4]  Dietitian manager engaged (providing support to catering department, supervising students etc.) [Hospital 1,3]  Catering middle management (i.e. under assistant catering manager) engaged (involved in monitoring compliance, providing training and info for staff etc.) [Hospital 4] | Catering management engaged (manage resistance to change, involved in nutrition analysis, monitoring implementation etc.) [Hospital 2,3,4]  Hospital management and leaders engaged (external audit will lead to buy-in, to monitor compliance) [Hospital 2]  Vacant assistant catering manager role filled so can be engaged in implementation [Hospital 2] | *… we did not have buy-in from all of the managers. So from my point of view I would have felt that they were taking one step forward and two steps back.* [Hospital 4 - barrier]  *No I don’t feel any support from hospital management. I think it’s more it’s being asked of them is it done. And then obviously if they say no it’s been asked why not and get it done. There’s no direct support to get it done.* [Hospital 2 – barrier]  *The fact that all the senior management, so the general manager and the senior administrative were very much behind the initiative and very supportive of the initiative and approached it in a very collective way. I think that’s probably why I think we’ve done reasonably well with it.* [Hospital 1 - facilitator]  *I think we’ve a very enthusiastic catering manager with very good leadership in the department you know and would see the big picture and also would have a very keen interest in nutrition himself.* [Hospital 4 - facilitator] |
| Available Resources | Lack or limited resources [Hospital 1,2,3,4]  Lack of time [Hospital 1,2,3,4]  Lack of money/funding [Hospital 2,3]  Lack of equipment (labelling machine, bread slicing machine, computers) [Hospital 3,4]  No physical space for students to work [Hospital 4] | Money/funding [Hospital 1]  Physical space to display calories [Hospital 2] | Adequate resources [Hospital 1,2,3,4]  Adequate time [Hospital 1,2,4]  Money/funding [Hospital 1,2,4]  Access to equipment [Hospital 2,3] | *Obviously everything is resource dependent. So we would have had a very peripheral role in terms of the actual implementation of calorie posting in the catering department in this instance. We would have been supporters of it but I suppose most of it would have been done by the catering department.* [Hospital 1 – barrier]  *I mean I felt it was loaded on top of me and it was I could either sink or swim. And I think I’m still sinking with it to be honest with you because I haven’t been able to have time to do it myself you know…* [Hospital 4 – barrier]  *Plenty of room in there to calorie post so they had plenty of space when we did do it for that month.* [Hospital 2 – facilitator]  *So if we’re resourced for it we’ll do it, absolutely.* [Hospital 1 – future facilitator] |
| Access to Knowledge & Information | Lack of information (on implementing calorie posting, including best practice guidelines or using nutrition analysis software) [Hospital 1,2,3,4]  Lack of or inadequate training [1,2,3,4]  Lack of access to information required (no pcs, staff not sharing, lost on computer system, or other hospitals within group not sharing) [Hospital 2,4]  Lack of access to suitable nutrition analysis software [Hospital 1] | Access to information (from students, dietitians and national lead on calorie posting) [Hospital 1,2,3,4]  Access to suitable nutrition analysis software (as made available for patient related analysis) [Hospital 1,2,3]  Access to websites on Google (for standardised recipes and calorie content) [Hospital 1,2]  Access to training [Hospital 4] | Access to training (formal training for both existing and new catering staff) [Hospital 1,2,3,4]  Access to (clear) information on implementing calorie posting [Hospital 1,2,3]  Access to suitable nutrition analysis software [Hospital 1] | *You know there’s a lack of consistency in their management of portions because there’s no training for the staff.* [Hospital 4 – barrier]  *I do feel that if they have given resources or given training… And like a day seminar isn’t enough. I do feel like they should have given a week’s training or something to each. Like catering departments have a huge influence on what patients and customers and staff eat.* [Hospital 4 – barrier]  *For the last two years we’ve been getting a student from [county name] doing a degree in nutrition… she concentrated solely on standardising all our recipes… and inputting all that on to Nutritics and was able to give us calorie counts on it… great to get this information that we could then put up then.* [Hospital 2 - facilitator]  *So we got the licence for it because it was driven by the fact that we had to have the nutritional analysis done for the patient menus that was one of our criteria for the HIQA standards that we had to have in the hospital… Once we got the licence for that package that was by far and away more suitable to do calorie posting and it was a lot easier.* [Hospital 3 – facilitator]  *So I suppose we need to do the education of the catering and I suppose the importance of it…* [Hospital 2 – future facilitator] |
| **Characteristics of Individuals** | No data | No data | No data |  |
| **Process** |  |  |  |  |
| Planning | Lack of planning (due to lack of time) [Hospital 1,2,3] | Developing a plan [Hospital 1,4] | Conduct a training needs assessment [Hospital 1]  Use gap analysis tool to plan implementation [Hospital 2] | *But it’s having the time to actually sit down and kind of go okay so this is what we need to do, you know outline the plan and actually have the time to do it.* [Hospital 2 – barrier]  *I suppose we put it on the agenda very early on as to okay what do we need to do to really get buy-in into this. Do we do it a piece at a time? Do we do it for all meals all the time?* [Hospital 1 – facilitator]  *I think even with the recently launched food nutrition policy you know the way we can do a gaps analysis on that. So you identify what we’re not doing and then we have to put things in place in order to meet those deficits. Something like that would help with planning.* [Hospital 2 – future facilitator] |
| Engaging |  |  |  |  |
| Opinion Leaders | No data | No data | Engaging catering staff in positions of influence [Hospital 4] | *More audits will certainly help bring on board people who are in positions of influence within their own section…* [Hospital 4 – future facilitator] |
| Formally Appointed Internal Implementation Leaders | (Assistant) catering manager not engaged (role vacant, lack of resources to assist, unable to supervise students due to lack of time and relevant expertise, lack nutrition expertise, not supportive of policy) [Hospital 1,2,3,4]  Catering middle management not engaged (lack of buy-in, lack of nutrition expertise) [Hospital 4] | (Assistant) catering manager engaged - actively involved in implementation, supervising students and monitoring compliance (due to researcher visit, winning HSE national award, hospital culture, supportive of policy) [Hospital 1,2,3,4]  Catering middle management engaged - involved in implementation, providing training and monitoring compliance (in anticipation of researcher visit) [Hospital 4] | (Assistant) catering manager engaged (ensure vacant role filled, supportive of policy, managing resistance to change, monitoring compliance) [Hospital 1,2,3]  Catering middle management engaged (involved in nutrition analysis, monitoring and ensuring compliance) [Hospital 2,3] | *In the hospital you know in terms of say the general manager for the catering area, he was I suppose aware that it had to be implemented. But at the same time you know I just still feel that if the staff came back and said well look we have to look after the patient menus, we have to be compliant with HIQA requirements he was quite happy for the calorie posting to fall into second place.* [Hospital 2 – barrier]  *And yeah [catering manager name] absolutely led it with me. Like the two of us would have led it I suppose. But because [catering manager name] has an interest in it you know I found that… Ours was led way before other hospitals that I would have been in touch with in relation to it.* [Hospital 4 – facilitator]  *I think it might be best coming from the manager or the person over the canteen… You know people only ever listen to their own manager or the person who’s actually in charge of them… their best placed to monitor compliance.* [Hospital 3 – future facilitator] |
| Champions | Hospital committee leader stepping down (advocate for calorie posting - momentum now lost) [Hospital 3] | Hospital committee leader - advocate for calorie posting [Hospital 3] | Staff member committed to and driving calorie posting [Hospital 3] | *There’s more needs to be done there. But at the time then I stepped down as the Healthy Ireland lead and the group haven’t met since. And that was last September. And the momentum got completely lost… The focus just went totally off it.* [Hospital 3 – barrier]  *If it had a member of staff who was very committed to it themselves and driving it, that would be helpful.* [Hospital 3 – future facilitator] |
| Internal Key Stakeholders* | Catering staff not engaged or limited engagement (due to chef culture, resistance to change, no incentive, no time or resources, lack of consumer demand or engagement, no access to pcs, inadequate staffing levels and under-resourced, lack of knowledge and expertise, no designated person for implementation, risk of unsuccessful implementation and lack of buy-in) [Hospital 1,2,3,4]  Dietitians not engaged or limited engagement (due to lack of time or resources – so patients are priority, inadequate staff levels and under-resourced department - leading to not enough time for existing staff and patients then priority and no food services dietitian to implement) [Hospital 1,2,3,4]  Hospital management and leaders not engaged (Hospital 1,2,3,4]  Indirect stakeholders not engaged or limited engagement (due to vacant role, inadequate staffing levels leading to patients being priority, no committee meetings and poor communication, lack of knowledge and expertise and no health promotion department or staff) [Hospital 1,2,3,4]  Catering staff not engaged by catering management, facilities manager or HSE [Hospital 2,3,4]  Indirect stakeholders not engaged by catering manager [Hospital 1,4] | Catering staff engaged by catering management, facilities manager, dietitians, suppliers, students, researcher and HSE [Hospital 1,2,3,4]  Catering staff engaged (due to hospital culture, good relationships within department, achieving awards, receiving recognition, designated chef for staff canteen, compatible with what they do, internal buy-in and wanting to meet consumer needs) [Hospital 1,3,4]  Dietitians engaged (supportive of policy, involved in nutritional analysis, providing information, student supervision, monitoring accuracy) [Hospital 1,2,3,4]  Dietitians engaged by being part of HSE Policy Group [Hospital 1]  Hospital management and leaders engaged (due to researcher visit, awards, being supportive of policy) [Hospital 1,2,3,4]  Internal key stakeholders engaged (due to hospital culture, buy-in and easy to network and communicate in hospital) [Hospital 1,2,4] | Catering staff engaged (create a dedicated position or allocate a person for implementation, ensure adequate staffing levels and resources, adapt the intervention so more standardised across hospitals) [Hospital 1,2,3,4]  Catering staff engaged by catering management (more involved in implementation) [Hospital 3,4]  Catering staff engaged by colleges providing training (Hospital 2,3]  Dietitians engaged (recruit food services dietitian to help with implementation, ensure adequate staffing and resource levels) [Hospital 1,2,4]  Hospital management engaged (due to more external audits and being supportive of policy) [Hospital 1,2]  Indirect stakeholders engaged (ensure adequate resources, show intervention outcomes, achieve awards) [Hospital 1] | *When they would go up into the kitchen there wasn’t that much buy-in… the kids did find it difficult to access the chefs and get the information that they needed.* [Hospital 4 – barrier]  *I felt I wasn’t in a position resource wise to do much with it. Okay now priority was patients and we were struggling to deliver on that side. Struggling on the side of delivering the minimum standards that needed to be delivered in terms of patient care. So how on earth could I deliver and allocate any resources into the staff side.* [Hospital 1 - barrier]  *Well I know it was coming on board but I don’t think we really had any involvement in it as catering staff. No we had no real involvement.* [Hospital 3 – barrier]  *The fact that all the senior management, so the general manager and the senior administrative were very much behind the initiative and very supportive of the initiative and approached it in a very collective way. I think that’s probably why I think we’ve done reasonably well with it.* [Hospital 1 – facilitator]  *… there was a huge input from the dietetics department. I think that was really key as well, the support in the dietetics department I would say would have been.* [Hospital 1 - facilitator]  *If I had the time I could be able to sit down and get my head around it and get an understanding of it you know. An understanding of the MenuCal you know and how it works and that.* [Hospital 4 – future facilitator]  *I do think there needs to be a sense from the top management that it is as important as the patient down into the food.* [Hospital 2 – future facilitator] |
| Consumers (IS)* | Lack of consumer compliance with standardised portions [Hospital 1,2,3,4]  Consumers not engaged (don't notice the calories or don't know they are there) [Hospital 1,3]  No consumer (service user group) input into implementation [Hospital 1] | Consumers engaged (providing feedback) [Hospital 1] | Consumers engaged and supportive of calorie posting [Hospital 2] | *Well what I would say to you in answer to that is they would all know what a portion is. But a lot of the time what happens is people will say will you give me a bit more, can I have more. A lot of the time. You’d never have anyone asking for less.* [Hospital 4 – barrier]  *Staff coming behind it would certainly help.* [Hospital 2 – future facilitator] |
| External Key Stakeholders* | HSE not engaged (provide limited or none of the following: follow-up support, monitoring, recognition, resources for implementation. Also delays or embargo on recruitment and don’t see the importance of staff health and wellbeing etc.) [Hospital 1,2,3,4]  Suppliers not engaged (don’t have the required ingredients) [Hospital 1,2,4]  External key stakeholders not engaged (by catering manager) [Hospital 1,4] | HSE engaged (via developing policy and standards, providing awards, monitoring and training) [Hospital 1,2,3,4]  Suppliers engaged (providing nutrition information) [Hospital 3] | HSE engaged (to purchase national licence for nutrition analysis software, to provide calorie display units or specify which ones, provide resources and training, provide recognition for hospital progress with implementation, introduce monitoring and national award for achievement) [Hospital 1,2,3,4] | *I was hoping that a plan would have been put in place by the HSE to actually help hospitals to implement this thing but they didn’t. It was left basically to each hospital to do their own.* [Hospital 4 – barrier]  *Well nobody from the time that national lead on calorie posting left, nobody really came back to us and said well where are you on the calories, do you need help, do you need more training, do you need resources. There has been no contact.* [Hospital 3 – barrier]  *It was a national policy so I suppose like any of the national policies we would within reason try and do what we could to make it happen.* [Hospital 1 – facilitator]  *I think things like awards would be great… is something that will motivate the other stakeholders as well to do the different pieces of work that they need to do so that we can get this recognition, this external quality assurance award. So I think something like that that can be built into some kind of internal HSE award system would be good.* [Hospital 1 – future facilitator] |
| External Change Agents | Limited engagement of students (delayed in completing tasks due to no desk/pc, difficulty in accessing information required for implementation due to lack of buy-in from catering middle management, lack of appropriate supervision or monitoring, lack relevant nutrition expertise and no longer involved in implementation as placement finished) [Hospital 1,2,3,4]  Limited engagement of Irish Heart Foundation (no on-site support, lack of enforcement) [Hospital 3,4] | Students engaged (completing tasks related to calorie posting and sharing knowledge and information with catering staff) [Hospital 1,2,3,4]  Obtaining an award from the Irish Heart Foundation [1,2,3,4]  Students engaged by catering and dietetic department (receiving supervision, input and feedback and support) [Hospital 3,4]  Researcher conducting study [Hospital 2,4]  External catering group assisted with implementation [Hospital 2]  Public health experts promoting calorie posting [Hospital 1] | Students to complete tasks related to calorie posting (on placement each year) [Hospital 1,2,3,4]  Colleges to train up hospital staff [Hospital 3]  Irish Heart Foundation to conduct more audits [Hospital 3]  Irish Heart Foundation awards [Hospital 1]  Students to receive appropriate supervision (from dietitians) [Hospital 4] | *Now she’s gone and there won’t be much done until a student comes here again next January.* [Hospital 3 – barrier]  *And to be fair to the students who came in they came in raw and they wouldn’t be familiar with the catering end of it. I mean they’re okay at the office end of it. But they wouldn’t have the knowledge of the catering end… So their inexperience in that sense made it even harder to do the MenuCal for each dish you know.* [Hospital 4 – barrier]  *Without the student we wouldn't be this far at all… You might have a small section of the menu but you wouldn't have it all done.* [Hospital 3 – facilitator]  *If you had the calories displayed and all them boxes ticked, you got the gold. I suppose that drove us… we wanted to get the gold... we wanted to achieve the gold.* [Hospital 3 – facilitator]  *When you were coming [researcher name] we used it as a tool kit for us if you like to say that while it wasn’t an audit we did use the word audit. And we found which was surprising, I actually found it very surprising that some people really went checking and double checking everything which they should have been doing from day dot anyway… definitely when we told them that you were coming on site to do the audit we did see that there was a little bit of interest shown in relation to checking it.* [Hospital 4 – facilitator] |
| Executing | Inability to implement successfully (and provide accurate calorie information) [Hospital 1,2] | No data | Ability to implement successfully [Hospital 4] | *Well if you’re going to be doing it I feel you should be doing it right. And you’re misinforming people I think if you give one as I said two big slices and another one two small slices. I feel you’re misinforming them especially with that side of it.* [Hospital 2 – barrier] |
| Reflecting & Evaluating | Lack of or inadequate monitoring of implementation progress overtime [Hospital 2,3,4]  Lack of monitoring implementation outcomes (i.e. adherence) [Hospital 1,4] | Monitoring implementation outcomes (i.e. adherence and accuracy) – by catering management and dietitians [Hospital 1,4]  Monitoring implementation progress and providing feedback [Hospital 4] | Monitoring implementation outcomes (i.e. adherence) - by catering management and students [Hospital 1,2,3,4]  Use gap analysis tool to monitor implementation progress [Hospital 2] | *Like implementing it and then just expecting it to be done I think. And no feedback. Like no feedback to say if we are doing it right.* [Hospital 4 – barrier]  *One of the dietitians, she was a fantastic help. Like while we were implementing I had asked [dietitian name] would she check every day, so she’d come in we’ll say check the salad bar, have a look, throw her eye at different things and come back with feedback which she did. And we’d rectify that and move on to the next thing.* [Hospital 4 – facilitator]  *What they would do is I think it would make sense, it would be prudent to do at least a yearly review where what we have achieved as a standard and the calorie posting implementation is sticking so it has sustained itself. That is not a case of grand we’ve implemented it and you walk away… So the student that we would have coming in in January who has been secured now, she will be, her first task will be to actually review where we are, has there been any slippages, are there any gaps to the standard that we’re at, and what further refinements can we do to improve what we’re doing.* [Hospital 4 – future facilitator] |
| Adapting the Intervention* | No data | Adopting an approach were calorie posting forms part of a comprehensive programme [Hospital 1]  Analysing three portions of each menu item to get average calorie content [Hospital 1]  Printing menu with calories as oppose to writing on blackboard [Hospital 1] | A template for calorie posting to help standardise across all hospitals [Hospital 2,3]  Introduce a more practical calorie display method [Hospital 2,4]  Policy to include a tool to help measure compliance (e.g. a gap analysis tool) [Hospital 2,4]  User-friendly guidelines on best practice [Hospital 2]  Group related policies together (so only required to undertake one gap analysis) [Hospital 2]  Adopt an approach were calorie posting forms part of a comprehensive programme [Hospital 3] | *So I suppose in terms of resources we had to get a bit clever when the new chef came in. They were spending half an hour writing this stuff up every morning on a blackboard but now it’s printed and it’s posted so it’s much easier.* [Hospital 1 – facilitator]  *Until we get to a format where we have everything electronically displayed we’re always gonna have that where it can happen where one item we think that we have calorie posted all our dishes for lunch but one has been missed for whatever reason as human error. It will be easier in so far as you can update the information pretty much instantaneously. So there’ll be no more printing and it’s just there.* [Hospital 4 – future facilitator] |
| Adapting the Organisation* | No data | Introducing utensils to assist with standardising portions [Hospital 1,2,3,4]  Introducing set menus (no changes or variations) [Hospital 2]  Reducing the number of menu items [Hospital 1]  Introducing standardised recipes and portions [Hospital 1]  Establishing the yield per batch [Hospital 4]  Using more bought in or pre-packaged menu items (less home-made) [Hospital 2]  Using whole chicken breasts for curry, not chopped up [Hospital 3] | Introducing standardised recipes and portions (for different cultural dishes) [Hospital 2] | *But did anyone ever really know the recipe except the person? You know there was some secrets in there… So yes, they eventually came up with the magic formula for all the recipes and it just grew out of there.* [Hospital 1 – facilitator]  *So we have all our utensils in place… that we need for portion control. But now particularly with the salad bar we have all our salad spoons all the exact size required and we have the calorie posting for each level spoon of the spoon that we have highlighted on the salad bar.* [Hospital 4 – facilitator]  *There is but we have different cultures working here with us. We have Indians so they like to do Indian curry which can be very nice and tasty. So what I would like to see done is standardised recipes and Indian curry used on a certain day and a calorie count done on that the way everyone will know exactly what they’re eating.* [Hospital 2 – future facilitator] |
| Scaling Up* | No data | Gradually introduced calorie posting across menus (i.e. breakfast, then lunch menu) [Hospital 1,3,4]  Gradually undertook steps relating to calorie posting (i.e. entered store cupboard items into analysis software, then weighed recipe ingredients, then wrote recipe, then entered into software for calories) [Hospital 2,3]  Gradually implemented calorie posting over a number of years [Hospital 3,4] | No data | *And I think change is better slow and steady all the time rather than this knee jerk, you change it too fast it’ll just sit equally as fast. And it’s a bit like I heard, and I won’t name the hospital but we were asked the question how long did it take us to implement it and we said well we’re still implementing calorie posting but in terms of where we are and the standard we’re at its probably taken us four or five years. And they nearly fell off their seats because they had achieved it in two or three days. And I said it’s not possible to do it in two or three days.* [Hospital 4 – facilitator] |
| Strategy** | No data | Preparatory work (in advance of implementation) [Hospital 3]  Sharing plan publicly to have accountability [Hospital 4] | Develop business case to highlight resource need [Hospital 1] | *So we flag customers what’s pending and the dates that we were hoping to implement because when you’ve put a date and you put it out there you kind of stick to it. And we do it a lot because it puts pressure on us to make sure that we do it when we say we’re gonna do it.* [Hospital 4 – facilitator] |

**Symbols: * = new construct generated inductively from recent systematic review [**[**1**](#_ENREF_1)**], ** = new construct generated inductively from the study data**

**Abbreviations: IS = inner setting, OS = outer setting, KPI = key performance indicator**

**Reference**

1. Kerins C, McHugh S, McSharry J, Reardon CM, Hayes C, Perry IJ, et al. Barriers and facilitators to implementation of menu labelling interventions from a food service industry perspective: a mixed methods systematic review. ‎Int J Behav Nutr Phys Act. 2020;17:48.
