# Supplementary material for: Factors Influencing Fidelity to a Calorie Posting Policy in Public Hospitals: A Mixed Methods Study
Source: Front Public Health. 2021 Aug 13;9:707668. doi: 10.3389/fpubh.2021.707668 (PMC8414889; doi:10.3389/fpubh.2021.707668)
Supplement: Supplementary file 4 [file Table_4.DOCX]

| **Additional file 4. Matrix of barriers coded to domains and constructs by hospital** | | | | |
| --- | --- | --- | --- | --- |
| **Domains & Constructs** | **Hospital 1**  **(n = 9 interviews)** | **Hospital 2**  **(n = 7 interviews)** | **Hospital 3**  **(n = 6 interviews)** | **Hospital 4**  **(n = 8 interviews)** |
| **Intervention Characteristics** | **Most** | **Many** | **Manyˣ** | **Allˣ** |
| Intervention Source | None | None | None | None |
| Evidence Strength & Quality | None | None | None | None |
| Relative Advantage | None | Few | None | None |
| Adaptability | None | None | None | None |
| Trialability | None | None | None | None |
| Complexity | Some | Some | Someˣ | Most |
| Design Quality & Packaging | Most | Many | Manyˣ | Mostˣ |
| Cost | Few | Few | None | Few |
| **Outer Setting** | **Some** | **Most** | **Many** | **Some** |
| Consumer Needs & Resources (OS) | None | None | None | None |
| Cosmopolitanism | Few | Few | None | None |
| Peer Pressure | None | None | None | None |
| External Policy & Incentives | Few | Many | Many | Some |
| Economic Climate* | Few | None | None | None |
| Educational System* | None | Few | None | None |
| Culture (OS)** | None | Many | None | Few |
| **Inner Setting** | **Allˣ** | **Allˣ** | **Allˣ** | **Manyˣ** |
| Structural Characteristics | Some | Mostˣ | Some | Some |
| Networks & Communications | Few | Some | Some | Few |
| Culture (IS) | Many | Mostˣ | Many | Some |
| Consumer Needs & Resources (IS)** | Allˣ | Many | Mostˣ | Many |
| Implementation Climate | Most | Allˣ | Mostˣ | Manyˣ |
| Tension for Change | Some | Many | None | Few |
| Compatibility | Most | Most | Many | Someˣ |
| Relative Priority | Most | Mostˣ | Most | Some |
| Hospital Incentives & Rewards | None | Some | Fewˣ | Few |
| Goals & Feedback | None | None | None | None |
| Learning Climate | None | None | None | None |
| Readiness for Implementation | Most | Mostˣ | All | Many |
| Leadership Support | Some | Manyˣ | Many | Some |
| Available Resources | Some | Mostˣ | Many | Some |
| Access to Knowledge & Information | Many | Many | Most | Many |
| **Characteristics of Individuals** | **None** | **None** | **None** | **None** |
| **Process** | **Allˣ** | **Allˣ** | **Allˣ** | **Allˣ** |
| Planning | Few | Few | Few | None |
| Engaging | Allˣ | Allˣ | Allˣ | Allˣ |
| Opinion Leaders | None | None | None | None |
| Formally Appointed Internal Implementation Leaders | Few | Manyˣ | Few | Many |
| Champions | None | None | Some | None |
| Internal Key Stakeholders* | Most | Allˣ | All | Most |
| Consumers (IS)* | Manyˣ | Some | Manyˣ | Some |
| External Key Stakeholders* | Some | Someˣ | Someˣ | Manyˣ |
| External Change Agents | Few | Few | Some | Some |
| Executing | Few | Few | None | None |
| Reflecting & Evaluating | Few | Some | Some | Many |

**Note on magnitude: none = 0, few = 1-25%, some = 26-50%, many = 51-75%, most = 76-99%, all =100% of interviews in each hospital that mentioned the domain and construct as a barrier.**

**Symbols: * = new construct generated inductively from recent systematic review [**[**1**](#_ENREF_1)**], ** = new construct generated inductively from the data, ˣ = domain or construct noted in unstructured observation as a barrier.**

**Abbreviations: IS = inner setting, OS = outer setting**

**Reference**

1. Kerins C, McHugh S, McSharry J, Reardon CM, Hayes C, Perry IJ, et al. Barriers and facilitators to implementation of menu labelling interventions from a food service industry perspective: a mixed methods systematic review. ‎Int J Behav Nutr Phys Act. 2020;17:48.
