# Supplementary material for: Factors Influencing Fidelity to a Calorie Posting Policy in Public Hospitals: A Mixed Methods Study
Source: Front Public Health. 2021 Aug 13;9:707668. doi: 10.3389/fpubh.2021.707668 (PMC8414889; doi:10.3389/fpubh.2021.707668)
Supplement: Supplementary file 5 [file Table_5.DOCX]

| **Additional file 5. Matrix of facilitators coded to domains and constructs by hospital** | | | | | |
| --- | --- | --- | --- | --- | --- |
| **Domains & Constructs** | **Hospital 1**  **(n = 9 interviews)** | **Hospital 2**  **(n = 7 interviews)** | **Hospital 3**  **(n = 6 interviews)** | **Hospital 4**  **(n = 8 interviews)** |  |
| **Intervention Characteristics** | **Few** | **None** | **None** | **Few** |  |
| Intervention Source | None | None | None | None |  |
| Evidence Strength & Quality | None | None | None | None |  |
| Relative Advantage | None | None | None | Few |  |
| Adaptability | None | None | None | None |  |
| Trialability | None | None | None | None |  |
| Complexity | None | None | None | None |  |
| Design Quality & Packaging | Few | None | None | None |  |
| Cost | None | None | None | None |  |
| **Outer Setting** | **Most**ˣ | **Most**ˣ | **Many**ˣ | **Many**ˣ |  |
| Consumer Needs & Resources (OS) | Few | Few | None | Few |  |
| Cosmopolitanism | Few | None | Few | None |  |
| Peer Pressure | Some | Few | Few | None |  |
| External Policy & Incentives | Mostˣ | Mostˣ | Manyˣ | Manyˣ |  |
| Educational System* | None | None | None | Few |  |
| **Inner Setting** | **Allˣ** | **Manyˣ** | **Allˣ** | **Allˣ** |  |
| Structural Characteristics | Many | Fewˣ | Some | Few |  |
| Networks & Communications | Many | Few | Some | Many |  |
| Culture (IS) | Many | None | Some | Someˣ |  |
| Consumer Needs & Resources (IS)** | Most | Many | None | Some |  |
| Implementation Climate | Mostˣ | Many | Mostˣ | Manyˣ |  |
| Tension for Change | Some | Some | None | Some |  |
| Compatibility | Manyˣ | Some | Manyˣ | Manyˣ |  |
| Relative Priority | None | None | None | Few |  |
| Hospital Incentives & Rewards | None | None | Some | None |  |
| Goals & Feedback | Few | None | None | None |  |
| Learning Climate | Few | Few | None | Few |  |
| Readiness for Implementation | Mostˣ | Many | Mostˣ | Mostˣ |  |
| Leadership Support | Most | Many | Manyˣ | Manyˣ |  |
| Available Resources | Few | Few | None | None |  |
| Access to Knowledge & Information | Someˣ | Some | Some | Many |  |
| **Characteristics of Individuals** | **None** | **None** | **None** | **None** |  |
| **Process** | **Allˣ** | **Manyˣ** | **Mostˣ** | **Allˣ** |  |
| Planning | Few | None | None | Some |  |
| Engaging | Allˣ | Manyˣ | Mostˣ | Allˣ |  |
| Opinion Leaders | None | None | None | None |  |
| Formally Appointed Internal Implementation Leaders | Most | Some | Someˣ | Manyˣ |  |
| Champions | None | None | Few | None |  |
| Internal Key Stakeholders* | Most | Many | Fewˣ | Most |  |
| Consumers (IS)* | Few | None | None | None |  |
| External Key Stakeholders* | Many | Some | Many | Few |  |
| External Change Agents | Mostˣ | Manyˣ | Mostˣ | Allˣ |  |
| Executing | None | None | None | None |  |
| Reflecting & Evaluating | Few | None | None | Many |  |
| Adapting the Intervention* | Few | None | None | None |  |
| Adapting the Organisation* | Most | Few | Some | Some |  |
| Scaling Up* | Some | Few | Some | Few |  |
| Strategy** | None | None | Fewˣ | Few |  |

**Note on magnitude: none = 0, few = 1-25%, some = 26-50%, many = 51-75%, most = 76-99%, all =100% of interviews in each hospital that mentioned the domain and construct as a facilitator.**

**Symbols: * = new construct generated inductively from recent systematic review [**[**1**](#_ENREF_1)**], ** = new construct generated inductively from the data, ˣ = domain or construct noted in unstructured observation as a facilitator.**

**Abbreviations: IS = inner setting, OS = outer setting**

**Reference**

1. Kerins C, McHugh S, McSharry J, Reardon CM, Hayes C, Perry IJ, et al. Barriers and facilitators to implementation of menu labelling interventions from a food service industry perspective: a mixed methods systematic review. ‎Int J Behav Nutr Phys Act. 2020;17:48.
