# Supplementary material for: Factors Influencing Fidelity to a Calorie Posting Policy in Public Hospitals: A Mixed Methods Study
Source: Front Public Health. 2021 Aug 13;9:707668. doi: 10.3389/fpubh.2021.707668 (PMC8414889; doi:10.3389/fpubh.2021.707668)
Supplement: Supplementary file 7 [file Table_7.DOCX]

| **Additional file 7. Construct relationships** | | |
| --- | --- | --- |
| **Relationships** | **Sample Quotes** | **Hospital** |
| Structural Characteristics (-) > Culture (IS) (-) | *We’re a very traditional hospital and we’re a country hospital so it would have been believed that you know the more you gave the better.* [Quote from direct stakeholder – hospital 1] | 1 |
| Structural Characteristics (-) AND Culture (IS) (-) > Relative Priority (-) | *You know your plan is you have your bed capacity but when you go beyond the capacity there’s the demand that takes from us, coupled then with high absenteeism of staff. So it’s kind of a juggling game most days. And they are doing their best I suppose to try and address the basic requirement and then working on these other items that are still on the agenda as well.* [Quote from indirect stakeholder]  *Equally the building they’re in or the facility they’re in is just, it’s not fit for purpose anymore either. You know the number of people they’re catering for. You know I’ve often said to them they should be doing one or the other because I don’t know how they do it all in there. You know at the end of the day their priority is always going to be in patients. And from the dietitian’s perspective you know I would equally say that my priority will be for the patients. So if I’m going to be deciding whether to do a project on calorie posting for staff, even though staff welfare is hugely important if I’m more concerned about modified consistencies and not being the right consistency that’s going to take precedence.* [Quote from indirect stakeholder]  *We have a much bigger hospital… greater commitment to the therapeutic diets required here…But I do feel that it’s not for the lack of trying here. You know I do feel it’s just other things will take precedence over it.* [Quote from indirect stakeholder] | 2 |
| Structural Characteristics (+) > Networks & Communications (+) | *I mean we’re not a massive site if you know what I’m trying to say and everyone knows everybody. The people that were part of the group new how to network the system… It’s very easy to network and communicate very quickly.* [Quote from indirect stakeholder] | 1 |
| Structural Characteristics (+) > Available Resources (+) | *But other than that I mean the size of the canteen… It had actually just been refurbed and moved back. It had been moved out a few times because construction work was going on above it and then they moved back into it again. So that was probably maybe 2016. So it was quite a new canteen at that point. Plenty of room in there to calorie post so they had plenty of space when we did do it for that month. You know we put the A4 stand up along the hot counter. Smaller ones in along the walls beside the toast and the butter and the jams and yoghurts.* [Quote from indirect stakeholder] | 2 |
| Structural Characteristics (+) > Available Resources (+) > Adapting the Intervention (+) | *Yeah we’ve actually applied for funding only last week for that digital menu board. Through one of the foundations within the hospital, the Tallaght Hospital foundation. They have grants so we’ve applied for a grant for that. That should really help.* [Quote from direct stakeholder] | 4 |
| Structural Characteristics (-) > Available Resources (-) > Relative Priority (-) | *Resources are definitely worse now. They’re worse now because we only started off with like nine wards. We’ve twenty odd wards. We still have the same amount of staff. And we have plating. We’ve way more people coming into the canteen you know so it’s just literally crazy every day. Like it’s crazy like. Calorie posting just not a priority.* [Quote from direct stakeholder]  *We have a much bigger hospital than others, with more complex patient type requiring more therapeutic diets… so we just don’t have the time to do anything else unlike the other hospitals within this group that are smaller… But I do feel that it’s not for the lack of trying here. You know I do feel it’s just other things will take precedence over it.* [Quote from indirect stakeholder] | 2 |
| Structural Characteristics (-) > Available Resources (-) AND Culture (-) > Relative Priority (-) > Engaging: Internal Key Stakeholders (-) | *There have been issues with resourcing in the catering department. Obviously there have been resourcing issues in our department as well at times. Particularly I suppose it’s been maternity vacancies... I’ve two maternity vacancies this year. As long as they’re backfilled you know. Otherwise we’re in total crisis situation. But most certainly if we were down 50% staff we have absolutely no time to look at anything for staff. Patients only you know.* [Quote from indirect stakeholder – hospital 1]  *There was a lack of resources and there is a lack of resources in the canteen… not enough staff. Those resources were always being placed first and foremost on the patient food.* [Quote from indirect stakeholder – hospital 2]  *Patients are absolutely the priority because we don’t have enough staff across the board. It’s extremely stretched. Yeah. We don’t have resources to take on anything outside. We don’t even have the resources to manage the clinical service never mind the health promotion type aspects which are very very important but we just have to prioritise the patient work.* [Quote from indirect stakeholder – hospital 4]  *I suppose they’d know really that we are very very stretched and our time has been limited. And they would have known that from previous projects because they know that we haven’t even been able to do nutritional analysis for patient menus so they knew that we were very stuck… we don’t have a dedicated dietitian to work with. We don’t have a patient food services dietitian to work on such initiatives with patients or all the health promotion initiatives in the catering department. So as I said we support them where we could but there would be a very limit to what we would do.* [Quote from indirect stakeholder – hospital 4] | 1, 2, 4 |
| Structural Characteristics (-) > Available Resources (-) > Engaging: Internal Key Stakeholders (-) | *But again the dietitian department is under resourced and they might have given some support but it wasn’t on the ground in the kitchen.* [Quote from indirect stakeholder – hospital 2]  *There was kind of an issue at the time with staff and that. You know we were kind of short staffed so we just really didn’t have the time as such to be implementing calorie posting.* [Quote from direct stakeholder – hospital 2]  *The reason why the relationship isn’t great is because the catering departments need this resource working with them but they need to see someone who is working with them, not someone who actually… Its not that dietitians don’t want to get involved. It’s just that they don’t have the time or the resources because they’re not resourced to do so. And that then creates a bad relationship, bad atmosphere you know.* [Quote from indirect stakeholder – hospital 4] | 2, 4 |
| Structural Characteristics (-) > Relative Priority (-) > Engaging: Internal Key Stakeholders (-) | *We’re just… I don’t think we have enough staff to do that you know so it’s kind of been put on the long finger you know.* [Quote from direct stakeholder – hospital 4] | 4 |
| Structural Characteristics (-) > Leadership Support (-) AND Engaging: Internal Key Stakeholder (-) | *So as I said to you earlier I’ll be very interested to see now after your report is published what actually happens. If your findings rule that we need a project team or extra staff to implement the calorie counting in hospitals that don’t have it done, will that be supported and who supports it. Because obviously if I require one staff member to do it, will the money be forthcoming from my budget or will our CEO request that the budget comes from Healthy Ireland or what not. So there’s a lot of politics in play to implement it.* [Quote from direct stakeholder] | 2 |
| Structural Characteristics (-) > Leadership Support (-) AND Engaging: Formally Appointed Internal Implementation Leaders (-) | *There was only one catering manager in there with no assistant trying to run a whole big service, catering service… and she had very little time you know… there’s a finite amount of capacity there to deliver on all of these things here.* [Quote from indirect stakeholder – hospital 1]  *The primary issue is lack of management staff to implement calorie posting.* [Quote from direct stakeholder – hospital 2]  *There’s a big gap here at the moment in terms of management staff and doing all that… including calories.* [Quote from direct stakeholder – hospital 2] | 1, 2 |
| Structural Characteristics (-) > Leadership Support (-) AND Engaging: Formally Appointed Internal Implementation Leaders (-) > Engaging: Internal Key Stakeholders (-) | *Massive issues with lack of management resources. We’ve plenty of staff on the ground in terms of catering staff, catering assistants and chefs. The issue is management of these staff.* [Quote from indirect stakeholder] | 2 |
| Structural Characteristics (-) > Leadership Support (-) AND Engaging: Internal Key Stakeholders (-) > Access to Knowledge & Information (-) AND Engaging: Internal Key Stakeholders (-) | *We are trying to address this in that we have asked for the facilities manager here to allow us to do a day long training for both ward and main kitchen staff. And we’ve been looking for it for I would say this is our third year. We’re kind of fed up with it now to be honest. They initially kind of wanted us to do it over an hour and I said no, it has to be a full day. So there’s a delay in it being rolled out because to do with staffing levels again. Now we were told last year it would be done this year. We keep moving. It’s just the number at the end. So 2016, 2017, 2018, 2019, and now it’s going to be 2020 we’ve been told that we can roll out this training.* [Quote from indirect stakeholder] | 2 |
| Structural Characteristics (-) > Engaging: Internal Key Stakeholders (-) | *We don’t have any health promotion department at this hospital site… Now there is health promotion staff in many other hospitals but we don’t have any on our site… So there is no assistance with doing these things.* [Quote from indirect stakeholder]  *It’s nearly a full-time job for one person. But that was loaded on top of me shortly after I started in the head chef role you know. So I didn’t know what I was taking on to be honest with you you know… I wasn’t involved really…* [Quote from direct stakeholder – hospital 4]  *But then here you’ve to wait so long for everything and it’s probably the same every place. We’ll just say we wanted something up on the salad bar for signage. So you have to email we’ll say tech services. You’ve to wait for someone to come up. I think that took three weeks by the time they ordered it. You know so you’re depending on so many people.* [Quote from direct stakeholder – hospital 4] | 1, 4 |
| Structural Characteristics (+) > Engaging: Internal Key Stakeholders (+) | *So really to be quite honest with you we need somebody released to do it… to analyse the recipe, feed us the calories* [Direct stakeholder – hospital 1]  *You think that it would facilitate the likes of implementing calorie posting and other staff policies if there was a dedicated member of staff…* [Indirect stakeholder – hospital 2]  *Someone needs to be released for that project to implement it*. [Direct stakeholder – hospital 3]  *Since I came on board there’s an awful lot of change. Because it used to be just general in the kitchen but now I’m just the canteen. So I can concentrate on the staff food and all that…* [Direct stakeholder – hospital 4] | 1, 2, 3, 4 |
| Structural Characteristics (-) AND Compatibility (-) > Adapting the Intervention (+) | *And that would support the implementation so much if it was a case of we have one set of menu boards, they come from this supplier, it’s been done through a national vender. That is all you have to do. Because adding another step in the way of here’s three or four different venders. You’ve to go price it yourselves. You’ve to figure out who’s your cheapest price. You have to show your three quotes. You’ve to protect the quality of it and make sure it’s not a melting device. You know there could be another step there. That would definitely help.* [Quote from indirect stakeholder] | 2 |
| Culture (IS) (+) > Compatibility (+) | *Yeah like I mean if you’re working in a hospital you know you should probably have that whole ethos of you know healthy kind of living. They make it relatively easy for you to do that. They’re always having things up you know about fun runs and things like that so you can keep active and healthy.* [Quote from direct stakeholder] | 3 |
| Culture (IS) (+) > Learning Climate (+) | *They’re all looking and willing to learn and stuff like that. Since I came here now, and I’m only here a couple of weeks and they’re looking for positives and they’re looking for ways to be shown because everyone likes to learn something new you know.* [Quote from direct stakeholder] | 1 |
| Culture (IS) (-) > Relative Priority (-) | *I would say the patients would always take priority… patients come first. They will prioritise getting everything 100% for the patients but the staff canteen suffers as a result.* [Quote from indirect stakeholder – hospital 1]  *Yeah the primary thing here is patient care. Everything else is secondary… calorie posting is just not a priority.* [Quote from direct stakeholder – hospital 2]  *Generally, the patients always come first because if we were short anything the patient would have to get it first. The staff would be second. You know what I mean. The patient would always come first.* [Quote from direct stakeholder – hospital 2]  *All they’re interested in is patients… There’s no-one looking at it from this end at all you know… Nothing to do with staff in the canteen.* [Quote from direct stakeholder – hospital 3]  *When it had to be done for the patient menus it was a big priority. Being done for the staff it was take it or leave it.* [Quote from direct stakeholder – hospital 3]  *I don’t think there’s as big a focus on the health of the staff through the catering food as there is on the nutritional requirements of patients.* [Quote from indirect stakeholder – hospital 3]  *Patients are the focus here… they are our priority, not staff.* [Quote from indirect stakeholder – hospital 4] | 1, 2, 3, 4 |
| Culture (IS) (-) > Relative Priority (-) > Leadership Support (-) AND Engaging: Formally Appointed Internal Implementation Leaders (-) AND Engaging: Internal Key Stakeholders (-) | *In the hospital you know in terms of say the general manager for that area she was I suppose aware that it had to be implemented. But at the same time you know I just still feel that if the staff came back and said well look we have to look after the patient menus, we have to be compliant with HIQA requirements she was quite happy for the calorie posting to fall into second place. You know they’re aware it has to be done and everything couldn’t be done at once, but patients come first.* [Quote from indirect stakeholder] | 2 |
| Culture (IS) (-) > Relative Priority (-) > Leadership Support (-) AND Engaging: Internal Key Stakeholders (-) > Available Resources (-) | *So you know they may say that they are supportive but really we’re not looking for a lot of money at all at all… Because again when you think about it if you’ve got 50 people on a trolley they are above catering and all the rest, which is their priority.* [Quote from indirect stakeholder] | 2 |
| Leadership Support (-) AND Engaging: Internal Key Stakeholders (-) AND Culture (IS) (-) > Relative Priority (-) > Engaging: Internal Key Stakeholders (-) | *In the hospital you know in terms of say the general manager for that area, she was I suppose aware that it had to be implemented. But at the same time you know I just still feel that if the staff came back and said well look we have to look after the patient menus… she was quite happy for the calorie posting to fall into second place. You know they’re aware it has to be done and everything couldn’t be done at once…* [Quote from indirect stakeholder – hospital 2]  *You know this has to come from very senior levels because nutrition is important not just for our patients like even patients out of hospital but it’s also very important for keeping staff healthy. But nobody seems to see that as a starting point. You keep your staff happy and healthy, you’ve healthier patients, happier patients.* [Quote from indirect stakeholder – hospital 4] | 2, 4 |
| Culture (IS) (+) > Leadership Support (+) AND Engaging: Formally Appointed Internal Implementation Leaders (+) AND Engaging: Internal Key Stakeholders (+) AND Tension for Change (+) | *And as I said there’s a culture within the catering department firstly where they just love to serve staff and they want to give them the nicest and the best food possible and present it in the best way. There’s always been that strong tradition. So this was an easy one really because we just knew it was going to build on a good tradition and a good sound belief that they could do better.* [Quote from indirect stakeholder – hospital 1]  *I think if there’s a will there’s a way. There’s a bit of that here as well… We’re always striving to do a bit better. That’s basically it. There’s great energy. If we see the need for it, we will implement it.* [Quote from indirect stakeholder – hospital 1]  *If it’s the right thing to do because it’s an improvement you would.* [Quote from direct stakeholder – hospital 4]  *It wouldn’t be the driving force but kind of quality and doing it right would always supersede increase in revenue… it’s about just doing the right thing for the right reasons.* [Quote from direct stakeholder – hospital 4] | 1, 4 |
| Culture (IS) (-) AND Tension for Change (-) AND Available Resources (-) > Leadership Support (-) AND Engaging: Formally Appointed Internal Implementation Leaders (-) | *The reason for lack of buy is resistance, not wanting change. And I would say maybe part of that could well be personality. Part of it could be couldn’t give a dam. Part of it could be under-resourced.* [Quote from direct stakeholder] | 4 |
| Culture (IS) (-) > Engaging: Internal Key Stakeholders (-) | *But what we didn’t have was a staff that were particularly opened to change. It was a bit of a slog to get things done… anything done.* [Quote from indirect stakeholder – hospital 1]  *They were used to their old ways of working… resistant to change… so difficult to get them on board.* [Quote from indirect stakeholder – hospital 2] | 1, 2 |
| Culture (-) > Reflecting & Evaluating (-) | *I suppose I’ve been working in hospitals for 24 years and this particular site is very much project orientated. So if a project is due to have a deadline or a date the focus then is on that project. But once that date passes then the focus then is put on to something else. So this week it’s calories. Next week it could be hydration. The following week it could be waste management. There’s no oversight over the course of the year. It’s very much project focused… So instead of I suppose having calories on every single week its only project focused. It might be on because you came this week the focus is on calories. But then you’re gone now so the next project focused on calories could be when your report is due out.* [Quote from direct stakeholder – hospital 2]  *I suppose a lot of the work in a hospital is project based. So now that you’re here it’s giving us an incentive to ask questions why isn’t it being done. Where before it was never looked at to be honest. It might have been asked about but there was never really a rush to get it done. Now with you here doing your PHD on this subject there is an effort now to try and get it signed off and being completed. I think at the moment it’s a tick box exercise yeah. Just tick the box you know.* [Quote from direct stakeholder – hospital 2]  *What they would do is I think it would make sense, it would be prudent to do at least a yearly review where what we have achieved as a standard and the calorie posting implementation is sticking so it has sustained itself. That is not a case of grand we’ve implemented it and you walk away. Because a lot of change and a lot of initiatives if you don’t. It’s about the review and the monitoring. It’s like food safety. If you don’t review and monitor its just not gonna stick. And you know you create the kind of atmosphere or the vision or whatever for change. And you do it. And a lot of people say grand yeah that’s it and sit back. So the student that we would have coming in in January who has been secured now, she will be, her first task will be to actually review where we are, has there been any slippages, are there any gaps to the standard that we’re at, and what further refinements can we do to improve what we’re doing.* [Quote from direct stakeholder – hospital 4]  *I think they take on these projects and they look and sound good… They’re ticking boxes and that you know… But it’s a tick the box. It looks done. But is it really done? No one really checks… I don’t think we have fully implemented calorie posting yet...* [Quote from direct stakeholder – hospital 4] | 2, 4 |
| Networks & Communications (-) AND Structural Characteristics (-) > Engaging: Internal Key Stakeholders (-) | *At the time then I stepped down as the Healthy Ireland lead and the group haven’t met since. And that was last September. And the momentum got completely lost… The focus just went totally off it… Because your Healthy Ireland lead already has a job. So there isn’t the identification that there needs to be somebody’s specific role. It’s added on to your role.* [Quote from indirect stakeholder] | 3 |
| Networks & Communication (+) > Leadership Support (+) AND Engaging: Formally Appointed Internal Implementation Leaders (+) AND Engaging: Internal Key Stakeholders (+) | *It’s very easy to network and communicate very quickly… Everybody knows everybody you know and they’re all on first name terms so it’s very easy to get something fixed… this certainly helped with calorie posting.* [Quote from indirect stakeholder] | 1 |
| Networks & Communication (-) > Leadership Support (-) AND Engaging: Formally Appointed Internal Implementation Leaders (-) AND Engaging: Internal Key Stakeholders (-) | *There is a Healthy Ireland steering group but a lot of the meetings I think in the last year have been cancelled for different reasons. So I don’t know how active they’ve been recently but they have been active in the past. So lots of these things, including calorie posting, would have come up and been discussed by various people within the hospital…but no meetings so not discussed* [Quote from indirect stakeholder – hospital 1]  *So the communication bit I think is a weak point I think overall down there. This would not have helped in terms of implementing calorie posting, as people not linking together to get it done.* [Quote from indirect stakeholder – hospital 2] | 1, 2 |
| Networks & Communication (+) > Engaging: Internal Key Stakeholders (+) | *I think we have a good team…Like you don’t want everybody to be yes people. Things have to be challenged. But when an initiative makes sense and you know when you’re trying to bring people to a place it has to make sense to them as well. So yeah we’ve got largely a good team, that work well together. And yeah we’re lucky in that sense.* [Quote from direct stakeholder] | 4 |
| Networks & Communications (+) > Planning (+) | *Even before it came I would have had a link with the catering department in terms of staff health and staff choices and all of that. So when the HSE calorie posting policy was established or was published or whatever the word is. I suppose we put it on the agenda very early on as to okay what do we need to do to really get buy-in into this. Do we do it a piece at a time? Do we do it for all meals all the time? And do we drive it as a healthy Ireland initiative or a nutrition and standards initiative.* [Quote from indirect stakeholder] | 1 |
| Consumer Needs & Resources (OS) (+) AND Consumer Needs & Resources (IS) (+) > Tension for Change (+) | *Consumers need to know what is in their food… Obesity is a huge thing in Ireland and that’s why they need to know.* [Quote from direct stakeholder – hospital 1]  *You see I think that we should be doing it whether they want it or not to be honest with you… by 2030 half of us are going to be overweight. Do you know what I mean? I kind of think just because we don’t want to do it or just because you don’t like the idea doesn’t mean that we shouldn’t do it if it’s the right thing to do. So I think that we should do it…* *I think most people know what they should be doing. A lot of people just decide you know they just don’t want to do it and that’s fine. But I think you know by degrees you would hope that you’ll get the couple. And even if you only get a couple every week you know they’re more aware.* [Quote from indirect stakeholder – hospital 2]  *… it was needed because of you know obesity in the country and weight management and all of that kind of stuff, and we knew it was a good thing to do.* [Quote from direct stakeholder – hospital 4] | 1, 2, 4 |
| Consumer Needs & Resources (IS) (+) AND Culture (+) > Tension for Change (+) | *The incentive is we do genuinely want to do the right thing by the people we’re serving, you know the patients and visitors and staff. So that would be our drive and driving factor.* [Quote from direct stakeholder] | 4 |
| Consumer Needs & Resources (IS) (+) AND Compatibility (+) > Leadership Support (+) AND Engaging: Formally Appointed Internal Implementation Leaders (+) AND Engaging: Internal Key Stakeholders (+) AND Tension for Change (+) | *Look it’s all to do with giving our customers more information and it’s about providing information that might hopefully make people healthier and more aware of what they’re eating. And it’s close to what a couple of us in here, we’re kind of big into all that you know healthy eating, healthy living and sports and stuff. It aligns at a personal level but also at a professional level definitely…* *At a professional level you want to do the right thing by the people that you’re giving the service to… And we always want to if there’s something that needs to be done or needs to be improved… Yeah we knew there was gonna be work and it was gonna be challenging but yeah we said let’s do it.* [Quote from direct stakeholder] | 4 |
| Consumer Needs & Resources (IS) (-) > Compatibility (-) | *You know we have changed the menu here in the last couple of weeks and there’s been a positive response over it. Not ideal for calorie posting, but consumers want new menu items.* [Quote from direct stakeholder] | 1 |
| Consumer Needs & Resources (IS) (-) > Relative Priority (-) | *I’d be more concerned about the allergen information than the calorie information for patients…*  *For staff again I’d say the allergen is probably more important for staff…*  *Well for allergens if you were allergic to something say flour or something more serious that’s gonna cause you lots of issues for the customer… To me allergens is more important than the calories.* [Quote from direct stakeholder] | 2 |
| Consumer Needs & Resources (IS) (-) AND Engaging: External Key Stakeholders > Compatibility (-) | *I suppose a lot of work has been done to standardise the recipes and analyse them. But the menus again change on an ongoing basis again based on food supply and based on feedback probably from staff… So the menus do change as well.* [Quote from indirect stakeholder] | 2 |
| Consumer Needs & Resources (IS) (+) > Leadership Support (+) & Engaging: Formally Appointed Internal Implementation Leaders (+) | *Well [catering manager name] is doing a lot of work. You can see here even by this like you know she’s coming in and she’s breaking all these things down for it as well you know and she’s making sure it’s up on the posters so people are informed.* [Quote from direct stakeholder] | 1 |
| Consumer Needs & Resources (IS) (-) > Engaging: Consumers (-) | *People always want bang for their buck as they say. Some people have smaller appetites and some people have bigger appetites, do you know what I mean. That’s the way. You can standardise as much as you like within a hot serve but you are going to be dealing with people that will look for more.* [Quote from direct stakeholder – hospital 1]  *They request extra portions or load the plate… they want a bit of value.* [Quote from direct stakeholder – hospital 2]  *So there’s no standardisation in terms of quantity… we tried to do it at one stage and we met a lot of resistance from staff using the canteen. They don’t want standardised portions… they want to load their plate.* [Quote from direct stakeholder – hospital 2]  *Now they did get in all the ladles and spoons. They’re all over there. The specific 3 ounce, 4 ounce ladles and spoons for serving for both the hot and cold. The salad portions as well. But like that they’re just going to take a scoop of coleslaw and it’s not going to be 3 ounces or 4 ounces or whatever it’s meant to be. It’s going to be quite high… And that’s more to do with getting value for their money as well you know.* [Quote from direct stakeholder – hospital 3]  *But I think maybe other people might feel a little bit the other way that why are you giving me less today than you were giving me the other day and I don’t want it to be enforced.* [Quote from direct stakeholder – hospital 3]  *Well what I would say to you in answer to that is they would all know what a portion is. But a lot of the time what happens is people will say will you give me a bit more, can I have more. A lot of the time. You’d never have anyone asking for less.* [Quote from direct stakeholder – hospital 4] | 1, 2, 3, 4 |
| Compatibility (+) > Access to Knowledge & Information (+) | *Because we’re the hospital we have to have Nutritics. But like because it’s already here and the licencing of that like that isn’t a huge issue.* [Quote from direct stakeholder – hospital 1]  *A lot of those same foods are served in the kitchen so there would have been some of that information that was all made available for the catering department.* [Quote from indirect stakeholder – hospital 1]  *So we got the licence for it because it was driven by the fact that we had to have the nutritional analysis done for the patient menus that was one of our criteria for the HIQA standards that we had to have in the hospital. So to carry out that analysis we needed Nutritics because that was the recommended package. There was you know no other real competitor for the analysis that would have done an adequate job. Once we got the licence for that package that was by far and away more suitable to do calorie posting and it was a lot easier.* [Quote from indirect stakeholder – hospital 3] | 1, 3 |
| Compatibility (+) > Leadership Support (+) AND Engaging: Internal Key Stakeholders (+) | *[Name of facilities manager] would be extremely supportive. He didn’t have to have really an active role but strangely enough he’s like me and like a lot of us in here he’s active, he’s into his health and he understands and he gets the importance of food and everything to do with food including calorie posting… so it was an easy sell. If I was saying to him look at this initiative we’re running with [name of facilities manager] he’d be enthusiastic about it and he would be inquisitive about it.* [Quote from direct stakeholder] | 4 |
| Compatibility (-) > Adapting the Organisation (+) | *And they have to look at that because we had no standardised recipes. We had a fantastic, there was one particular cook that’s now left years ago, and her brown bread was renowned, let’s put it that way. But did anyone ever really know the recipe except the person? You know there was some secrets in there… So we became much more formal you know to draft the recipe up. You know the menus and the ingredients were actually set out and they tightened all that up.* [Quote from indirect stakeholder – hospital 1]  *The information is there but there could be a 1% chance it might not be displayed depending on someone not changing the calorie display information in the service area… You know we live in the real world. In the real world like we have to flip menus over in the dining-room because it’s the same area that’s serving breakfast, mid-morning and lunch, so you have a different set of menus or a different set of offerings at different times of the day. So yes you’re relying on the guys who are on the ground, the guys who have the responsibility. That’s where you’re relying on people like the food and beverage manager or the dining supervisor and the girls on the ground to be doing the right thing at the right time. And you know occasionally I go down and the porridge is still up and the soup hasn’t been displayed. These are they’re kind of human errors…* *Until we get to a format where we have everything electronically displayed we’re always gonna have that where it can happen where one item we think that we have calorie posted all our dishes for lunch but one has been missed for whatever reason as human error.* [Quote from direct stakeholder – hospital 4] | 1, 4 |
| Incentives & Rewards (-) > Tension for Change (-) | *Why bother… to be honest about it, it’s more work… but no incentive to do it.* [Quote from direct stakeholder] | 2 |
| Tension for Change (-) > Engaging: Internal Key Stakeholders (-) | *But there wasn’t really the drive there… I just got a sense from the kitchen staff and the canteen staff that they just never really bought into it and that it was just as easy not to be putting these signs out every morning.* [Quote from indirect stakeholder]  *Whereas in the canteen itself some of the staff I encountered there’s a sense of why are we doing this at all.* [Quote from indirect stakeholder] | 2 |
| Relative Priority (-) > Leadership Support (-) AND Engaging: Formally Appointed Internal Implementation Leaders (-) | *To me allergens is more important than the calories. That’s not to say that we’re not going to do it. We will be doing it in conjunction with the allergens…* [Quote from direct stakeholder] | 2 |
| Relative Priority (-) > Engaging: Internal Key Stakeholders (-) | *I suppose the Healthy Ireland project management team is extremely busy. And within two months of that launch we were also publishing our own Healthy Ireland implementation plan. So we were completely side-tracked away from it yeah.* [Quote from indirect stakeholder] | 2 |
| Available Resources (+) > Access to Knowledge & Information (+) | *…the need for training being very important for the catering staff and there needs to be dedicated time for that to allow for this training.* [Quote from indirect stakeholder] | 2 |
| Available Resources (-) > Access to Knowledge & Information (-) | *When we went up looking for the Nutritics package we were told there was no money.* [Quote from direct stakeholder] | 3 |
| Available Resources (-) > Access to Knowledge & Information (-) > Engaging: Internal Key Stakeholders (-) | *The worry that I would have would be chefs. I don’t know how they would access the nutritional package. Do you know what I mean? The information would have to be there for them on a hardcopy type of thing because they wouldn’t have access to a PC. So wouldn’t be able to do nutritional analysis.* [Quote from direct stakeholder – hospital 4] | 4 |
| Available Resources (-) > Incentives & Rewards (-) > Engaging: Internal Key Stakeholders (-) | *There’s no incentive in it you know if you’re not given the time… like it is on top of what I’m already doing you know, something has to give.* [Quote from direct stakeholder] | 4 |
| Available Resources (-) > Relative Priority (-) > Engaging: Internal Key Stakeholders (-) | *Now could we have done it without them? We could have but it would have been much more of a challenge. Because a lot of it is around time and resources, particularly time. I think we have the capability and we have the knowledge base. It’s just when you’re in the cutting throat stuff, the day to day operation things catch up and it gets parked to the side.* [Quote from direct stakeholder – hospital 4] | 4 |
| Available Resources (-) > Engaging: Internal Key Stakeholders (-) | *Obviously everything is resource dependent. So we would have had a very peripheral role in terms of the actual implementation of calorie posting in the catering department in this instance. We would have been supporters of it but I suppose most of it would have been done by the catering department.* [Quote from indirect stakeholder – hospital 1]  *Because sometimes you get so busy running the day to day you don’t have time to be doing the nice little pieces or the extra little piece like calorie posting.* [Quote from direct stakeholder – hospital 1]  *And it’s literally down to you know both the dietitian and catering not having the resource to implement it.* [Quote from indirect stakeholder – hospital 2]  *Well I know it was coming on board but I don’t think we really had any involvement in it as catering staff. No we had no real involvement. We just didn’t have the resources.* [Quote from direct stakeholder – hospital 3]  *[Dietitian name] wouldn’t have time. No. No. She wouldn’t have the time to be involved.* [Quote from direct stakeholder – hospital 3]  *I mean I felt it was loaded on top of me and it was I could either sink or swim. And I think I’m still sinking with it to be honest with you because I haven’t been able to have time to do it myself you know.* [Quote from direct stakeholder – hospital 4]  *I feel I got absolutely no help [from the dietetic department] … reason being lack of resources.* [Quote from direct stakeholder – hospital 4] | 1, 2, 3, 4 |
| Available Resources (+) > Engaging: Internal Key Stakeholders (+) | *So if we’re resourced for it we’ll do it, absolutely. We will be more involved in projects such as calorie posting if resourced to do so.* [Quote from indirect stakeholder – hospital 1]  *But a factor in terms of being able to implement and deliver you know on the desired outcomes is you have to resource the departments to deliver.* [Quote from indirect stakeholder – hospital 1]  *If I had the time I could be able to sit down and get my head around it and get an understanding of it you know. An understanding of the Menucal you know and how it works and that.* [Quote from direct stakeholder – hospital 4] | 1, 4 |
| Available Resources (-) > Engaging: Internal Key Stakeholders (-) > Access to Knowledge & Information (-) | *Again it was a piece that we had to educate the caterers on saying you know that you need to make sure you have recipes, you need to make sure that they’re followed consistently, you need to make sure that if a recipe is to yield so many portions that it always does so, otherwise you’re at… And that actually in itself was always you know meeting with them and this talking at an ad hoc basis as opposed to a planned basis… Ad hoc knowledge giving as opposed to I wouldn’t even call it training. Knowledge giving. We just didn’t have the resources to give more to them.* [Quote from direct stakeholder] | 4 |
| Available Resources (-) > Engaging: Internal Key Stakeholders (-) > Reflecting & Evaluating (-) | *And you know I wasn’t able to give him time to even make sure they were doing things right… we should have been able to sit down with them… doing checks to make sure everything was working out.* [Quote from indirect stakeholder] | 4 |
| Available Resources (-) > Leadership Support (-) AND Engaging: Formally Appointed Internal Implementation Leaders (-) > Engaging: External Change Agents (+) | *And you know we had a catering manager who was interested and took in a student because she certainly absolutely didn’t have the time to do it herself.* [Quote from indirect stakeholder – hospital 1]  *But here I don’t personally have time to do it. I can oversee it but my student is doing it because I haven’t got the time directly to give it that time.* [Quote from direct stakeholder – hospital 2]  *It gives her the output of the scone has X amount of calories or a dinner has X amount. And then the students were coming in then. Obviously [catering manager name] hadn’t time. She hasn’t the time to do it all.* [Quote from direct stakeholder – hospital 3] | 1, 2, 3 |
| Available Resources (-) > Leadership Support (-) AND Engaging: Formally Appointed Internal Implementation Leaders (-) > Engaging: External Change Agents (-) | *And we’ve been lucky enough that last year we had an outstanding student. This year again the girl is quite good. So we’re lucky that we can literally give them free hand and I’m not directly supervising them. They are coming in and they’re doing a project away from me. And in fairness I’m not giving them much supervision which isn’t right either at the same time you know. I’m expecting a lot of them without much input to them which is just the way it is at the moment. I just don’t have the time.* [Quote from direct stakeholder – hospital 2]  *I wasn’t best placed to observe the work of the students and its quality. I absolutely feel I did not have the time nor the necessary qualifications to do it.* [Quote from direct stakeholder – hospital 4] | 2, 4 |
| Available Resources (-) > Engaging: Internal Key Stakeholders (-) > Engaging: External Change Agents (+) | *We don’t have the resources to currently to do it. So we will have to wait till the next student comes here again.* [Quote from direct stakeholder – hospital 3]  *We’ve all the knowledge, we could do it… but we don’t have the time to do it. So that’s why they bring in students.* [Quote from direct stakeholder – hospital 4] | 3, 4 |
| Available Resources (-) > Engaging: Internal Key Stakeholders (-) > Engaging: External Change Agents (-) | *And you know I wasn’t able to give him time to even make sure they were doing things right. Now I did crosscheck some of the analysis they came up with. And some were very wrong. And I put them on the right track. And said look you have to be very careful when you see something that’s not… You know porridge made from water is coming up at like 300 calories per portion. You need to go back and check something like that. So there was a lot of in the beginning say looking back and checking that. And it was coming to me initially. And I said look I can’t’ keep doing this, I don’t have the time… we should have been able to sit down with them and be crosschecking the students and doing checks to make sure everything was working out.* [Quote from indirect stakeholder] | 4 |
| Available Resources (-) > Engaging: External Change Agents (-) | *So I had no PC, no desk, no nothing for the kids, the students sorry… So like when they came on to us they could only access the PC when one of us were off it. And as you can imagine we’re under-resourced anyway. It was a nightmare just trying to you know get accommodation for them. I found that was a problem as well and slowed down the work the students could do.* [Quote from direct stakeholder] | 4 |
| Available Resources (-) > Planning (-) | *But it’s having the time to actually sit down and kind of go okay so this is what we need to do, you know outline the plan and actually have the time to do it.* [Quote from indirect stakeholder] | 2 |
| Access to Knowledge & Information (-) > Engaging: Consumers (-) | *Like if we're not being trained in it how are we meant to sell it off to somebody else you know?* [Quote from direct stakeholder] | 3 |
| Access to Knowledge & Information (+) > Engaging: Consumers (+) | *I think maybe some sort of course introduction into calories or something for us so that we can make other people aware.* [Quote from direct stakeholder] | 3 |
| Access to Knowledge & Information (+) > Engaging: Internal Key Stakeholders (+) | *We are trying to address this in that we have asked for the facilities manager here to allow us to do a day long training for both ward and main kitchen staff… We need them to be released so that they can see okay this is important, I’ve been released for this. Also for them to get a certificate for them to know okay well I’ve done this training.* [Quote from indirect stakeholder] | 2 |
| Access to Knowledge & Information (-) > Engaging: Internal Key Stakeholders (-) | *I mean if you’re going to introduce a new policy or something new like that if they don’t embrace it nobody will. It’s not going to work without formal training. You know it’s just not going to work.* [Quote from direct stakeholder – hospital 3]  *I don’t really know because I didn’t do the computer end of it. I just handed over information that I hand wrote and the girls inputted it into the system. I wasn’t involved in the computer end of it, the I. T end of it you know… my I. T skills aren’t great. I’m trying to improve you know the more I get into the job you know. Now I’m not stupid either but the likes of that now I haven’t used it. [Production manager name] has used it as well, the production manager who’s not here. But between the two of them they’ve been doing it rather than me. I would just give them the information and they would input or whatever you know.* [Quote from direct stakeholder – hospital 4] | 3, 4 |
| Access to Knowledge & Information (-) > Leadership Support (-) AND Engaging: Internal Key Stakeholders (-) | *But you know I suppose maybe I haven’t understood the work that is involved to get out the calorie posting. You know my view would be that we just take every item, listing it and giving it a calorie count. Maybe I’m totally wrong. I can’t support them really when I don’t know what’s involved.* [Quote from indirect stakeholder] | 2 |
| Leadership Support (-) AND Engaging: Internal Key Stakeholders (-) > Incentives & Rewards (-) | *I used to work in the Rotunda and they did, they got a Q mark quality award the year I was in it. Now I don’t know what it’s like now or anything like that. I know things are changing. But like that because we got that award, now I’m talking nearly twenty years ago but because we got that award the catering department everybody got to go on a weekend away you know given to us by management, hospital management. Half the catering department went one week, the other half went the other week… It was to say well done and thanks and a great job… In this hospital, we feel undervalued and not seen by hospital management… Yeah if we get the happy heart, [catering manager names] are great like yeah that’s great, well done, everybody had an input. But that’s where it stops. You know why shouldn’t management say well done you know, well done, good job. I’m sure whatever happens on the wards whether it be a managerial position or a nursing position if they do well in something they’re recognised. You know so… But there is no recognition for catering.* [Quote from direct stakeholder] | 3 |
| Leadership Support (-) AND Engaging: Internal Key Stakeholders (-) > Available Resources (-) | *We’re gone to the stage where we have all the calorie information now and we’re looking for a labelling machine that we can label all our stuff. Its €1000 but we’re waiting for it. We’ve been looking for that since last February.* [Quote from direct stakeholder] | 3 |
| Leadership Support (-) AND Engaging: Internal Key Stakeholders (-) > Available Resources (-) > Access to Knowledge & Information (-) | *The facilities management are the ones with the pot of funding to help us set up the training. But they won’t release it… not sure how supportive of nutrition and calorie posting they are.* [Quote from indirect stakeholder] | 2 |
| Leadership Support (+) AND Engaging: Internal Key Stakeholders (+) > Available Resources (+) > Access to Knowledge & Information (+) | *And thankfully hospital management here have been very supportive and they’ve agreed to fund the licence. We can’t do good analysis… without a nutritional analysis software package, and Nutritics was the one we felt was best.* [Quote from indirect stakeholder] | 1 |
| Leadership Support (+) AND Engaging: Internal Key Stakeholders (+) > Relative Priority (+) AND Available Resources (+) | *And then I do think there needs to be a sense from the top management that it is as important as the patient down into the food. You know if there’s time and money resources going into the patient menus there needs to be time and resources going into standardising the staff menus and calorie posting them.* [Quote from indirect stakeholder] | 2 |
| Leadership Support (+) AND Engaging: Internal Key Stakeholders (+) > Engaging: External Change Agents (+) | *In fairness to the dietetic manager she saw an opportunity when we got the two students in and we’ve another round of students in. Every time we get students in we give them projects. It was through their projects we got this work done you know. And it was all supervised by the dietitians.* [Quote from indirect stakeholder] | 1 |
| Leadership Support (-) AND Engaging: Formally Appointed Internal Implementation Leaders (-) > Engaging: External Change Agents (-) | *So I spoke with her and thought it would be good for them you know to have the support of the student but she wouldn’t have been in a position to take the supervision duties of a student so early in her job... so they didn’t take a student.* [Quote from indirect stakeholder – hospital 1]  *… it was very stressful for them [referring to students] because I was their lead and then if I wasn’t here fair enough they had my PC but they didn’t have anyone to link in with or bounce off if you know what I mean… and this was slowed things down.* [Quote from direct stakeholder – hospital 4] | 1, 4 |
| Leadership Support (+) AND Engaging: Formally Appointed Internal Implementation Leaders (+) AND Reflecting & Evaluating (+) > Engaging: External Change Agents (+) | *[Assistant catering manager] would have worked very closely with them on that side of it and I would have. I’d be coming in and kind of just you know you’d look at it and then you’d say okay let’s pick a few and talk us through. Just to get the accuracy right. Yeah.* [Quote from direct stakeholder – hospital 4] | 4 |
| Leadership Support (+) AND Engaging: Formally Appointed Internal Implementation Leaders (+) AND Reflecting & Evaluating (+) > Engaging: Internal Key Stakeholders (+) | *And on a daily basis then [catering supervisor name] would monitor the breakfast and then before lunch would start. And whoever would be serving on the counter she would ask them if a customer asks you for a second spoon you explain to them that this is the portion size, the standardised size. And that would have been every day. And she still does it… so the catering staff were quite receptive… everybody had the knowledge.* [Quote from direct stakeholder – hospital 4] | 4 |
| Leadership Support (+) AND Engaging: Formally Appointed Internal Implementation Leaders (+) AND Strategy (+) > Engaging: External Change Agents (+) | *We had to be ready for the student. There was no point in having her if we didn’t have that information ready… it was then a better use of their time and expertise.* [Quote from direct stakeholder] | 3 |
| Leadership Support (+) AND Engaging: Formally Appointed Internal Implementation Leaders (+) & Engaging: Internal Key Stakeholders (+) > Engaging: External Change Agents (+) | *We shared her between ourselves and the dietitian which was great. Because then the dietitian didn’t have total responsibility or we didn’t. So if you were on a day off or something at least then there was somebody there for the student because somebody has to be with the student. So there has to be supervision with the student as well.* [Quote from direct stakeholder] | 3 |
| Leadership Support (-) AND Engaging: Formally Appointed Internal Implementation Leaders (-) AND Engaging: External Change Agents (-) > Engaging: Internal Key Stakeholders (-) | *Well I would to be doing the nutritional analysis because I like to stand over everything that has my name on it you know. But it’s just I haven’t had a proper input in it you know. When I do something I like to do it right you know. But it was kind of given to me but the students were doing it. I was just handing them over the information you know. So I don’t feel I have the proper knowledge you know. But yet people are still coming to me and asking me where is such and such and I can’t answer… So the students are gone and now they come to me… So there wasn’t enough support in that sense you know from management.* [Quote from direct stakeholder] | 4 |
| Leadership Support (+) AND Engaging: Formally Appointed Internal Implementation Leaders (+) > Engaging: Internal Key Stakeholders (+) | *The catering manager would have come around. But you know actually we used to be amused about everything. You know as I said to you like we couldn’t believe like in a portion of chips how many was in it and a half a portion. You’d be oh my God like. You know but every time that it was done or something different was done we were all brought together and told you know this is it. You know you’d have the portions as well.* [Quote from direct stakeholder – hospital 1]  *We’ll say coming up to that we had to get all the staff together and we had to get all our ladles and our spoons and whatever. And then [catering supervisor name] who works with me who is the supervisor, she got a photograph of each of the ladles different sizes and got the staff together then and said this is for veg, this is for… Because we had the ounces, the portions displayed up of this is what it’s supposed to be. But the staff were excellent and had absolutely no problem.* [Quote from direct stakeholder – hospital 4] | 1, 4 |
| Leadership Support (+) AND Engaging: Formally Appointed Internal Implementation Leaders (+) > Reflecting & Evaluating (+) | *Yeah sometimes you have people in there that want more and more. And then you have [catering manager name] standing beside you saying that’s extra.* [Quote from direct stakeholder – hospital 1]  *Someone needs to monitor compliance… So I think it needs to be the catering manager. You know people only ever listen to their own manager or the person who’s actually in charge of them.* [Quote from indirect stakeholder – hospital 3]  *There was initially an issue with lack of compliance but [catering manager name] would monitor and check... Yeah because if you’re going back to the same person all the time saying, I know you didn’t follow the recipe they know then they’re being watched… So then they were more inclined to follow the recipe.* [Quote from direct stakeholder – hospital 4] | 1, 3, 4 |
| External Policy & Incentives (+) > Consumer Needs & Resources (IS) (+) | *That started from when we introduced Operation Transformation about five years ago. Whenever Operation Transformation started we did it every year for the full what does it go on for, six or eight weeks and we followed the recipes each week from Operation Transformation. And that’s what started people being conscious of their calorie intake etc.* [Quote from direct stakeholder] | 4 |
| External Policy & Incentives (+) AND Structural Characteristics (+) > Consumer Needs & Resources (IS) (+) | *Well like I have found that working here at the moment you know there’s a big awareness of calorie posting because of Operation Transformation and it being the beginning of the new year. Because I find at the beginning of the new year there is a huge uptake in calorie counting and salads and healthy options. Further into the year you don’t get as much of a buy-in. And then come to the holiday time when people are going on holidays in the summer people have an awareness again because they themselves have. And because as well here we have 70% women there would be a greater awareness of calories as against men… we’ve also got if I look at the age profile of our customers I would say it’s on the lower scale. I’d say it’s between maybe 25 and 45 more so than the older cohort. So I think that they’re more interested in calories and you know healthy eating and healthy options as against energy dense food.* [Quote from direct stakeholder] | 1 |
| External Policy & Incentives (+) > Goals & Feedback (+) | *Implementation of calorie posting aligned with other hospital goals because I suppose we were getting on the whole Healthy Ireland agenda… the catering department and nutrition was right up there at the top in terms of it probably being one of our main aims and objectives.* [Quote from indirect stakeholder] | 1 |
| External Policy & Incentives (-) > Relative Priority (-) | *We have to get the allergens done first… It’s a legal requirement. It’s a legislative requirement whereas calorie posting wasn’t.* [Quote from indirect stakeholder]  *There’s be massive liability issues with inaccurate posting of allergens… So allergens would be a priority over calories.* [Quote from direct stakeholder] | 2 |
| External Policy & Incentives (+) > Cosmopolitanism (+) > Tension for Change (+) | *Well Healthy Ireland was seen as an agenda that the hospital had to, you had to get on board with it as part of the hospital groups things. So I could get things through under Healthy Ireland that people might have been trying to get through before that they couldn’t. So it was a definite supporter.* [Quote from indirect stakeholder] | 3 |
| External Policy & Incentives (+) > Cosmopolitanism (+) > Leadership Support (+) AND Engaging: Internal Key Stakeholders (+) | *And I think in the [Hospital Group name] you know Healthy Ireland has got a lot of traction and there’s different KPIs around Healthy Ireland in the [Hospital Group name]. So I think our hospital management have to report back every year on different things that they have done in terms of Healthy Ireland and being able to say that they’ve got calorie posting in place, being able to say they’ve done these different health promotion things for staff.* [Quote from indirect stakeholder] | 1 |
| External Policy & Incentives (-) > Structural Characteristics (-) > Leadership Support (-) AND Engaging: Formally Appointed Internal Implementation Leaders (-) | *Massive issues with lack of management resources. We’ve plenty of staff on the ground in terms of catering staff, catering assistants and chefs… The catering officer retired two years ago and previous to that she was sick for possibly a year. That post was left vacant. It was advertised last year. We had twelve applicants. Only one was deemed qualified through the NRS. That person wasn’t deemed suitable. And at the moment we are looking at addressing that through re-advertising, downgrading the job, changing the job spec, changing the job title. And at the moment we are not able to meet any of those. Specs are controlled by a union. The union are, while they will engage it could take three or four years as a process to initiate that.* [Quote from direct stakeholder] | 2 |
| External Policy & Incentives (+) > Leadership Support (+) AND Engaging: Internal Key Stakeholders (+) | *I do see you know the HIQA inspections are really what we need. This would mean hospital management would put into practice what they say they’re going to do and what they’re supportive of.* [Quote from indirect stakeholder – hospital 2]  *Like the two of us would have led it I suppose…And I took it because it was legislative.* [Quote from direct stakeholder – hospital 4] | 2, 4 |
| External Policy & Incentives (+) > Leadership Support (+) AND Engaging: Internal Key Stakeholders (+) > Available Resources (+) | *Having some sort of regulatory body monitoring these things would probably increase management support and there would then be more funding or investment in it.* [Quote from indirect stakeholder] | 2 |
| External Policy & Incentives (+) > Engaging: Internal Key Stakeholders (+) | *Oh purely the launch itself was the driver for calorie posting. You know we picked the date. It was sometime in April. It was national workplace wellbeing day. We had the CEO came down for a picture. We had to be calorie posting on that day. And it was a day pin prick in the calendar and it wouldn’t have happened without the support of health promotion, health and wellbeing and their own back room office and some support in dietitians. It absolutely wouldn’t have happened at all.* [Quote from indirect stakeholder] | 2 |
| Cosmopolitanism (-) > Access to Knowledge & Information (-) | *Well outside the hospital but within the group… they were still working in their own silos so there was no sharing of calorie information.* [Quote from indirect stakeholder] | 2 |
| Cosmopolitanism (+) > Leadership Support (+) AND Engaging: Formally Appointed Internal Implementation Leaders (+) AND Engaging: Internal Key Stakeholders (+) | *The [Hospital Group name] helped as well perhaps… calorie posting was added into the Healthy Ireland implementation plan for the [Hospital Group name] early on, as one of the KPIs for the group. So for every hospital in the group this was one of the nutrition KPIs you had to deliver on. So I think it got management support at a high level or at least on paper it did.* [Quote from indirect stakeholder] | 1 |
| Cosmopolitanism (+) > Leadership Support (+) AND Engaging: Internal Key Stakeholders (+) | *I think having a dietitian here on staff who was part of that national policy group. We were in tune with what was going on nationally. You know we have a greater insight and we were flagging it up earlier in our hospital... she would have had a lot of informal conversations with the catering department about what was going on as well.* [Quote from indirect stakeholder] | 1 |
| Cosmopolitanism (+) > Engaging: External Change Agents (+) | *… we’re gonna continue the relationship with them. So [catering manager name] has made links with colleges and we’re definitely gonna be taking a food science and a food nutritional student next year to help with implementation* [Quote from direct stakeholder] | 4 |
| Consumer Needs & Resources (OS) (+) AND External Policy & Incentives (+) > Leadership Support (+) & Engaging: Formally Appointed Internal Implementation Leaders (+) AND Tension for Change (+) | *There would be a small bit there in so far as you know you want to be at the cutting edge or ahead of it. So you want to be at least thinking the same way that policy makers are thinking. But when you look at the bigger picture of Ireland as a nation I suppose and where we are in terms of obesity and all that kind of stuff you can say well all this makes sense. So if you know that stuff is in draft form you say well its draft form, its gonna happen in a couple of years, it might be four or five, let’s just forge ahead.* [Quote from direct stakeholder] | 4 |
| Culture (OS) (-) > Engaging: Internal Key Stakeholders (-) > Compatibility (-) | *And also very much a sense that… the way JohnJoe made the shepherd’s pie was the way JohnJoe made it. You didn’t necessarily ever change the menu or weigh out his portion size so it was very difficult to standardise. And it was the sense that you were taking away his individuality and his right to create the dish the way he created it… It was a very political minefield. It wasn’t just as easy as stepping in and suddenly starting to standardise menus. Chefs have their individual way of doing things.* [Quote from indirect stakeholder – hospital 2]  *We weren’t being precise enough about the weighing of ingredients and the accuracy around the recipes…Because in the front of house we were allowing the scope for individual crafts to be demonstrated. So my skills and what I was good at and the recipes that worked for me may not necessarily have worked for somebody else so there was a lot of variation. But then with that variation there wasn’t the consistency. But we hadn’t quite grappled initially what calorie posting really actually meant so we were still carrying on with the variations. So while we were doing it it might be based on Chef A but Chef B, C, and D had different. And that’s a cultural thing.* [Quote from direct stakeholder – hospital 4] | 2, 4 |
| Educational System (-) AND Culture (OS) (-) > Engaging: Internal Key Stakeholders (-) > Compatibility (-) | *So now my curry is the same as four other chefs. All the Irish chefs do the same standard recipe for the Irish way of doing it. [Chef name] then it’s kind of you throw a bit in or a bit here and you throw a bit of this and you throw a bit of that in. And [chef name] is the same. Whereas I’d know mine off by heart. A two pound bag of flower. I’d know six pounds of marg. I’d know so many your KGs of chicken. I’d know I had one jar of chutney, one tin of puree. I’d know it and the other girls would be the same as mine. Do you know what I mean? They’d know. Your powder. They’d know exactly what I’m using and they would use the same because it’s the way we were taught.* [Quote from direct stakeholder] | 2 |
| Educational System (+) > Engaging: External Change Agents (+) > Leadership Support (+) AND Engaging: Formally Appointed Internal Implementation Leaders (+) | *And what I will say to you is I am getting better as every year goes on. Because the kids that are coming in as well teach me… because every year I find with the schools they seem to be learning more and more about it.* [Quote from direct stakeholder] | 4 |
| Economic Climate (-) > Structural Characteristics (-) AND Culture (-) > Relative Priority (-) > Engaging: Internal Key Stakeholders (-) | *We’ve gone through you know obviously the situation in the country with all the cutbacks… So your resources are cut so you can’t deliver all of your patient services. So you certainly are in less of a position to take on additional pieces of work like these.* [Quote from indirect stakeholder] | 1 |
| Engaging: External Change Agents (+) > Engaging: Internal Key Stakeholders (+) AND Access to Knowledge & Information (+) | *The girls were in here doing it, the students, and they were fairly well up on it all. They were very good like they would show you how to do it you know.* [Quote from direct stakeholder – hospital 1]  *It was easier when the students showed you how to do it if you know what I mean.* [Quote from direct stakeholder – hospital 1]  *We’ve been getting a student… she concentrated solely on standardising all our recipes and inputting all that on to Nutritics and was able to give us calorie counts on it.* [Quote from direct stakeholder – hospital 2]  *Our student will have to train our clerical officer how to do that…which helps.* [Quote from direct stakeholder – hospital 2]  *If the HSE tied in with the colleges to train up staff that would really help.* [Quote from direct stakeholder – hospital 3] | 1, 2, 3 |
| Engaging: Internal Key Stakeholders (-) > Engaging: External Change Agents (-) AND Access to Knowledge & Information (-) | *When the students would go up into the kitchen there wasn’t that much buy-in… the kids did find it difficult to access the chefs and get the information that they needed.* [Quote from direct stakeholder]  *I was the main person for the students. And the reason for that was again we did not have buy-in... So from my point of view I would have felt that they were taking one step forward and two steps back. Because I could only give them so much information but then I needed them to be able to link in with the chefs you know for what the chefs actually get the yield out of their products and that type of thing.* [Quote from direct stakeholder] | 4 |
| Engaging: Internal Key Stakeholders (+) > Engaging: Internal Key Stakeholders (+) AND Access to Knowledge & Information (+) | *We have one dietitian here on site who has done an awful lot of work with the catering department previously. She has analysed all the food and the rest of it. And so when we have our new dishes I’ll be using her please God. She’ll be able to feed into me as well and to feed the information back to me as well.* [Quote from direct stakeholder – hospital 1]  *Coming up to the launch [dietitian name] would have gone down and helped them about how to label things, how to show things… providing this information.* [Quote from indirect stakeholder – hospital 4] | 1, 4 |
| Engaging: External Key Stakeholders (-) > Engaging: Internal Key Stakeholders (-) AND Access to Knowledge & Information (-) | *I do think a HSE lead should have been put in, but they weren’t... that lead could have trained me or the likes of me up into identifying what was needed…* [Quote from direct stakeholder] | 4 |
| Engaging: External Key Stakeholder (+) > Engaging: Internal Key Stakeholders (+) AND Access to Knowledge & Information (+) | *For any of the bought products, we got in contact with the suppliers and they sent us down their sheet so that wasn’t too bad. You know like muffins or things like that. They sent down the whole thing so that was very easy.* [Quote from direct stakeholder – hospital 3]  *She [national lead on calorie posting] used to ring every now and again to ask us where we on it. And we used to tell her well we’ve just done the breakfast. And then she gave us then [name of exemplar hospital] had done the portions of mayonnaise. Yeah [name of exemplar hospital] had done the portions of mayonnaise so she had sent us down the sheet with the mayonnaise, tomato ketchup, mustard, tartar sauce and whatever else. Yeah we were all using the same products so we were. So that was so helpful.* [Quote from direct stakeholder – hospital 4] | 3, 4 |
| Engaging: Internal Key Stakeholders (+) > Reflecting & Evaluating (+) | *We asked them would they come down…with their dietetic hat on to critique… what has worked and what hasn’t in terms of signage but also the accuracy of the information that we’re displaying. Because you guys are well trained and where we would see things in relation to operational things, ye are able to say that’s just not right. And they did. In fairness with the poster they found three or four things that just didn’t square up and they said are you sure, and we had to go back. So that was when we were drafting. And we must have drafted both the big poster but also our accuracy of what we were doing. We had to kind of revise that a few times.* [Quote from direct stakeholder] | 4 |
| Engaging: Consumers (+) > Reflecting & Evaluating (+) | *The other day we had a staff member coming in and they were having their lunch and they were looking at the menu and they said how come the healthy option has more calories than another thing. But like that was an error on my behalf that I had the wrong thing or we had posted the wrong thing. But it just shows you that there is an awareness and that somebody was looking at it and feeding back to us the fact that it wasn’t as it should be or whatever. So we corrected it.* [Direct Stakeholder] | 1 |
| Engaging: External Key Stakeholders (+) AND External Policy & Incentives (+) > Available Resources (+) | *I think the award from the HSE has to be given directly to the catering department. So it could be I don’t know, money for a piece of equipment or a new display sign or something like that. I don’t think it should be given to the hospital because it might never come down through the catering budget… Funding to buy a piece of equipment yeah. Because you never have enough equipment or you always need to replace something.* [Quote from direct stakeholder] | 2 |
| Engaging: External Key Stakeholders (+) > Available Resources (+) | *HSE need to allocate resources before putting policies in place such as this.* [Quote from indirect stakeholder] | 4 |
| Engaging: External Key Stakeholders (-) > Available Resources (-) | *I think it is a good project. I just feel it was let down by not having it properly resourced and leaving it to you know departments to implement it themselves if you want to call it that. I do feel that there was no resources given to it, no accommodation, no computers, nothing.* [Quote from direct stakeholder] | 4 |
| Engaging: External Key Stakeholders (-) > Available Resources (-) AND Access to Knowledge & Information (-) | *I do feel that if they [referring to HSE] have given resources or given training… And like a day seminar isn’t enough… I do feel like they should have given a week’s training or something to each. Like catering departments have a huge influence on what patients and customers and staff eat.* [Quote from direct stakeholder] | 4 |
| Engaging: External Key Stakeholders (-) AND External Policy & Incentives (-) > Tension for Change (-) | *Where before it was never looked at by anyone in the HSE to be honest. It might have been asked about but there was never really a rush to get it done.* [Quote from direct stakeholder] | 2 |
| Engaging: External Key Stakeholders (+) AND External Policy & Incentives (+) > Tension for Change (+) | *Well we knew at some stage it would be… Look when something is coming from the HSE and it’s a policy and there’s a standard sooner or later you’re gonna have to do it* [Quote from direct stakeholder] | 4 |
| Engaging: External Key Stakeholders (+) AND External Policy & Incentives (+) > Leadership Support (+) AND Engaging: Formally Appointed Internal Implementation Leaders (+) AND Engaging: Internal Key Stakeholders (+) AND Tension for Change (+) | *And there was a national award. They went up to receive it. They got a great platform and recognition for their work. And that drove them. That led on to then the calorie posting. It became very easy.* [Quote from indirect stakeholder] | 1 |
| Engaging: External Key Stakeholder (+) > Leadership Support (+) AND Engaging: Internal Key Stakeholders (+) | *Not that I particularly recall. You know at the time we were nearly even looking for a key performance indicator. You know often times it would have been useful. I know at times the health and wellbeing commission insisted on a report and asked us which of our hospitals are calorie posting and to what level and we’d reply to them. But there was never as such you know a KPI that you could say the national acute division want to know how many of your hospitals are calorie posting and then that feeds down to the CEO and down to the general management. And then there’s the pressure because if it’s read why is it read. You know so while the health and wellbeing commission were asking us periodically where we were and what the status was to my mind a lot of the KPIs needs to come more from the operation end from the national acute because it’s seen to be part of normal business.* [Quote from indirect stakeholder] | 2 |
| Engaging: External Key Stakeholders (+) AND External Policy & Incentives (+) > Engaging: Internal Key Stakeholders (+) | *I think things like awards would be great because we can sell them to the rest of our nutrition hydration steering committee… We can sell them you know this is something we want to achieve. This is a quality status award for the hospital. It would motivate them.* [Quote from indirect stakeholder] | 1 |
| Engaging: External Key Stakeholders (-) > Structural Characteristics (-) > Engaging: Internal Key Stakeholders (-) | *The head chef of this hospital post was allowed to be vacant for eleven months, nearly a year you know. And yet they expect us to deliver on calorie posting and you know we don’t even have a head chef in place. You know why was there such a delay. There’s huge delays in recruitment.* [Quote from indirect stakeholder] | 1 |
| Engaging: External Key Stakeholder (+) > Structural Characteristics (+) > Engaging: Internal Key Stakeholders (+) | *If HSE wants dietetic involvement in this they’ve really gotta look at work post planning for dietetics. I mean obviously our profession has been looking at because other countries have catering dietitians and catering dietitian resources. There is a positions statement from our professional organisation the INDI, Irish Nutrition and Dietetic Institute of the role that a dietitian or a dietetic service that is resourced to provide some resources into catering and how that can add value and the key pieces of work that they can do if they’re resourced to do it how they can add and deliver on HSE and HIQA to help meet standards. Staff that can do these pieces of work, will really helped with implementing the likes of this policy and other similar ones.* [Quote from indirect stakeholder – hospital 1]  *We have equally from a nutrition committee put forward a submission for a food services dietitian… So the role of that person wouldn’t just be for calorie posting. It would be actually to implement you know the national policies that are out there. It could even be including things like the vending policy. Any of these things that should be mandatory. Because we’re kind of doing it but we’re not really…* [Quote from indirect stakeholder – hospital 2] | 1, 2 |
| Engaging: External Key Stakeholders (+) AND External Policy & Incentives (+) > Engaging: Internal Key Stakeholders (+) > Access to Knowledge & Information (+) | *I think having a dietitian here on staff who was part of that national policy group. We were in tune with what was going on nationally. You know we have a greater insight and we were flagging it up earlier in our hospital.* [Quote from indirect stakeholder]  *I suppose like my colleague being involved nationally we had a bit more insight here locally and she would have had a lot of informal conversations with the catering department about what was going on as well.* [Quote from indirect stakeholder] | 1 |
| Engaging: External Key Stakeholders (+) AND External Policy & Incentives (+) > Engaging: Opinion Leaders (+) | *People who make it challenging… These are people who are in positions of the influence within their own section. Now we can overstep that but you want people in sections embracing and going along with you with where you want to go. So yeah absolutely. Definitely more HSE audits will help.* [Quote from direct stakeholder] | 4 |
| Engaging: External Key Stakeholders (+) AND External Policy & Incentives (+) > Engaging: Internal Key Stakeholders (+) | *From the health and wellbeing point of view they were very supportive of calorie posting. It remains one of the few national policies we have. The calorie posting, vending and (unsure) are the only three that are nationally mandated policies we’re all meant to be working towards. So from a health and wellbeing point of view they were very supportive for it to be implemented.* [Quote from indirect stakeholder] | 2 |
| Engaging: External Key Stakeholder (+) AND External Policy & Incentives (+) > Leadership Support (+) AND Engaging: Internal Key Stakeholders (+) | *I would certainly be advocating you know because it’s our national policy the calorie posting in terms of in the HSE….so must continue with it.* [Quote from indirect stakeholder]  *And management were definitely supportive of it. And I think management driving it. This has to be done, as policy. They got on with it.* [Quote from indirect stakeholder] | 1 |
| Engaging: External Change Agents (+) AND External Policy & Incentives (+) > Engaging: Internal Key Stakeholders (+) | *The Heart Foundation award led to huge buy-in within the catering department* [Quote from indirect stakeholder] | 1 |
| Engaging: External Change Agents (-) > Engaging: Internal Key Stakeholders (-) AND Access to Knowledge & Information (-) | *And although we thought we had all the dishes calorie posted when we’d go to try and find them in the system we can’t find them you know… in our system that the students would have set up for the calorie posting. Say we have just a random figure of 200 dishes… When we go to look for that dish to give the information to the dining-room on the calorie posting a lot of the stuff we can’t find for some reason even though we did the groundwork with the students who did the Menucal. So we’re finding that we have to go and do it all again ourselves. And each dish is just taking an awful lot of time to do. They seem to have got lost in transit or lost in the system or whatever you know… or example we had to do it this morning. Myself and Val had to do it on beef and onion pie that’s on out in the dining-room. Although I know I did it with the students and the chef involved that we did this before. But yet we can’t find it in the system.* [Quote from direct stakeholder] | 4 |
| Engaging: External Change Agents (+) AND External Policy & Incentives (+) > Leadership Support (+) AND Engaging: Internal Key Stakeholders (+) > Available Resources (+) | *I think also like we’re on a couple of accreditation schemes so the fact that we could put ourselves forward for the Irish Heart Foundation healthy eating award, that kind of link in, the calorie posting was part of that… And the external accreditation holds a lot of weight and it’s very useful when you’re trying to negotiate around extra funding or funding that is linked to this kind of thing like it makes it easier to negotiate and to kind of keep a door open… particularly at the executive level with the CEO and [Name of Director of Facilities].* [Quote from direct stakeholder]  *It gives us outside accreditation. So it’s a positive thing for the department and for the hospital. But it also keeps doors open where you want them open in terms of trying to get funding for things. You’ve got a better chance when you’re performing well.* [Quote from direct stakeholder] | 4 |
| Engaging: External Change Agents (+) AND External Policy & Incentives (+) > Leadership Support (+) AND Formally Appointed Internal Implementation Leaders AND Engaging: Internal Key Stakeholders (+) AND Tension for Change (+) | *So now that you’re here it’s giving us an incentive to ask questions why isn’t it being done… there is an effort now to try and get it signed off and completed.* [Quote from direct stakeholder – hospital 2]  *And actually it was only [researcher name] with you coming in that I was able to cross the line to [production manager] and [head chef] … When you were coming [researcher name] we used it as a tool kit for us if you like to say that while it wasn’t an audit we did use the word audit. And we found which was surprising, I actually found it very surprising that some people really went checking and double checking everything which they should have been doing from day dot anyway… definitely when we told them that you were coming on site to do the audit we did see that there was a little bit of interest shown in relation to checking it.* [Quote from direct stakeholder – hospital 4] | 2, 4 |
| Engaging: External Change Agents (+) AND External Policy & Incentives (+) > Leadership Support (+) AND Engaging: Internal Key Stakeholders (+) > Incentives & Rewards (+) | *Somebody would come from the Irish Heart Foundation… if you had the calories displayed and all them boxes ticked you got the gold… I suppose that drove us… we wanted to get the gold. They [hospital management] put our picture in the paper when we won the happy heart award. And it went in the new e-zine magazine. So yeah they would recognise it… when you got the award and you got a well done from [hospital manager name] or whoever is here like you know it is nice you know… Recognition is important. Oh no it is yeah. Because as I say [catering manager names] might drive it and all the rest but it’s all your staff down the kitchen that are actually doing the work. And it’s a way of recognising them as well you know.* [Quote from direct stakeholder] | 3 |
| Engaging: External Change Agents (+) > Reflecting & Evaluating (+) | *What they would do is I think it would make sense, it would be prudent to do at least a yearly review where what we have achieved as a standard and the calorie posting implementation is sticking so it has sustained itself. That is not a case of grand we’ve implemented it and you walk away. Because a lot of change and a lot of initiatives if you don’t. It’s about the review and the monitoring. It’s like food safety. If you don’t review and monitor its just not gonna stick. And you know you create the kind of atmosphere or the vision or whatever for change. And you do it. And a lot of people say grand yeah that’s it and sit back. So the student that we would have coming in in January who has been secured now, she will be, her first task will be to actually review where we are, has there been any slippages, are there any gaps to the standard that we’re at, and what further refinements can we do to improve what we’re doing.* [Quote from direct stakeholder] | 4 |
| Engaging: External Change Agents (+) > Adapting the Organisation (+) | *[Name of external catering group] helped… They’re like a catering crowd that were in helping us assisting at the time… They were trying to implement a lot of set menus if you like so that you would know your calorie control and whatever you know what’s being used in each recipe basically so then it’s easier to do your calorie count if you like. You know if you have a set menu rather than us all doing different menus.* [Quote from direct stakeholder] | 2 |
| Engaging: Champions (-) > Networks & Communications (-) | *I think the working group is on hiatus at the moment because we had a leader of the group who was a real advocate and very vocal and you know really trying to promote all the aspects of Healthy Ireland for the hospital between activity and healthy eating and mindfulness and things like that. But she stepped down from the role as leader of the group and unfortunately nobody wanted to take over the role… So we’re not meeting at the moment. The group isn’t meeting.* [Quote from indirect stakeholder] | 3 |
| Executing (-) > Engaging: Internal Key Stakeholder (-) | *Fear how to do it. Are you doing it properly, are you doing it correctly. Do you know what I mean? Fear is a huge factor. Am I doing it wrong? If I do it wrong the responsibility is mine.* [Quote from direct stakeholder] | 2 |
| Strategy (+) > Engaging: External Key Stakeholders (+) > Available Resources (+) | *Well you know I suppose if it’s a resource issue looking at business cases to try and get the resources they need from the HSE so that they can deliver on it…. yes, business cases to highlight the resource need locally.* [Quote from indirect stakeholder] | 1 |
| Adapting the Intervention (+) > Planning (+) AND Reflecting & Evaluating (+) | *I think even with the recently launched food nutrition policy you know the way we can do a gaps analysis on that. So you identify what we’re not doing and then we have to put things in place in order to meet those deficits. Something like that… So I think that if there was a kind of a gaps analysis or something, some kind of a tool that you could use to complete to say are you compliant or not compliant you know and what is it you’re going to do… And even for ourselves that every year we can look at it and kind of go okay so have we progressed on this you know.* [Quote from indirect stakeholder] | 2 |
| Adapting the Intervention (+) > Compatibility (+) | *So I suppose in terms of resources we had to get a bit clever when the new chef came in. They were spending half an hour writing this stuff up every morning on a blackboard but now it’s printed and it’s posted so it’s much easier.* [Quote from direct stakeholder] | 1 |
| Complexity (-) > Consumer Needs & Resources (IS) (-) > Adapting the Intervention (+) | *The likes of your meat that you cook like two slices could be too small slices or you could have two big slices. It depends on if the breast of turkey, if you take two from the end they’re going to be smaller than two in the middle. So you can’t very well say two slices of turkey is so much calories… Well if you’re going to be doing it I feel you should be doing it right. And you’re misinforming people I think if you give one as I said two big slices and another one two small slices. Now you could have a thing up giving an average calorie count for what it would be and that would help.* [Quote from direct stakeholder] | 2 |
| Complexity (-) AND Available Resources (-) > Adapting the Organisation (+) | *We could do up to 35 portions of fresh fruit salad every day and it depends on what’s in it. Grapes, mandarins, whatever. And the calorie counts like there could be four grapes in this one, there could be two in this one… You’re not going to be saying one grape for you, one grape for you, one grape for you. Do you know what I mean? You just don’t have the time for that… So Noreen got fruit pots that were made up coming in because it would have said the calorie count and everything on them.* [Quote from direct stakeholder] | 2 |
| Design Quality & Packaging (-) > Access to Knowledge & Information (-) | *I wasn’t aware of any supporting material with the policy. There was a policy itself which gave some information but I’m not aware of anything else that could have help.* [Quote from direct stakeholder – hospital 1]  *No, there was no supporting information to help us. We were all kind of working at it ourselves through the internet… It was up to yourself to get on with it.* [Quote from direct stakeholder – hospital 2]  *I didn’t see anything any supporting materials with the policy. They didn’t come my way anyway. I didn’t get. You know I wasn’t shown them or I wasn’t asked my opinion on them. So I’m not even aware if there are any now. If there are it would be great to know.* [Quote from indirect stakeholder – hospital 3]  *There wasn’t I have to say… no supporting materials with the policy… nothing like that. You’d a policy and you had to implement it.* [Quote from direct stakeholder – hospital 4] | 1, 2, 3, 4 |
| Design Quality & Packaging (-) > Access to Knowledge & Information (-) > Engaging: External Change Agents (-) | *Just even recommendations or what you have to actually have in place for the calorie posting. It’s like we knew we had to have the nutritional analysis done and we were getting the energy content and the calories up for that. So therefore you know we just put them up. Whether we put them up per ounce or per portion I think it was just down to the student kind of feeling this was the most appropriate way to put it out there.* [Quote from indirect stakeholder] | 3 |
| Design Quality & Packaging (-) > Access to Knowledge & Information (-) > Adapting the Intervention (+) | *Again sometimes people don’t really know exactly what it is we’re looking for... the policy is too complicated. People need a document that’s kind of as plain as the nose on their face exactly what they’re looking for. It needs to be black or white… needs to be clear.* [Quote from indirect stakeholder – hospital 2]  *I would have thought a template. I would have thought you know regardless of what hospital you go into it should be easily identifiable straight away there should be a template for calorie posting… I’d like to see kind of a standardisation of it that that’s how it happens across the board and not as individual in different hospitals have it… I also think that makes it easier to implement when somebody says it needs to go into this format.* [Quote from indirect stakeholder – hospital 3]  *At this point in time no, we don’t look at how well it has been implemented. I’m sure there will be into the future I suppose. But normally when these policies come out the people who wrote the policy have some sort of way of auditing it. And as far as I’m aware I’ve never been given any direction in terms of an audit process for this so I don’t know if there is one. Oh I definitely think I mean if you’re gonna run something nationally the audit needs to be the same for everybody… You know so I think if there’s guidance on what the policy should be I think there’s a guidance about how we should measure it.* [Quote from indirect stakeholder – hospital 4] | 2, 3, 4 |
| Design Quality & Packaging (-) > Adapting the Intervention (+) | *Ordered clear perspex signs to display calories…the heat of the lights with the hot food was melting them. You know it was very difficult to find appropriate display merchandise. Whereas it could have just been a bulk delivery of forty or fifty display units and said these are the ones, use them, put them up, we know they won’t melt under the lights, put the Healthy Ireland branding on them. I mean we weren’t looking to be spoon fed but it could have been made a bit easier I thought.* [Quote from indirect stakeholder – hospital 2]  *The logistics of trying to keep the poster updated with new menu items and calories…just doesn’t work… We’ve actually applied for funding only last week for a digital menu board… what the electronic menu board will do it’s more a visual… once the information is typed up it will be easier to maintain.* [Quote from direct stakeholder – hospital 4] | 2, 4 |
| Design Quality & Packaging (-) > Engaging: Consumers (-) > Adapting the Intervention (+) | *There’s no need to be having it posted up all over the place because they’ll only become wallpaper… They won’t notice it then. You could have a generic one like an allergen one or something like that, do you know what I mean. But if you’re gonna have ten posters up they become wallpaper. Where you’ve one generic one that will probably be more beneficial than all those. That’s the way I would look at it anyway you know.* [Quote from direct stakeholder – hospital 1]  *We’ve it done but I don’t know if any of our staff really even notice it or even realise that it’s done. Whereas I feel that if the label was on the sandwich that if you’re picking up something, if you’re picking up a fruit with 95 calories on it or 98, whichever, and you’re picking up a scone with 450 you’re making a constructive choice because you can see it. You can’t say oh well I didn’t realise the scone had that many and the fruit is that, sure that’s a better choice. Even though we don’t have anything really that’s very high calorie. We just have regular stuff. But it means then they can choose.* [Quote from direct stakeholder – hospital 3] | 1, 3 |
| Design Quality & Packaging (-) AND Compatibility (-) > Adapting the Interventions (+) | *So you’d actually almost be looking at okay we’ve the chalk board where they write up what’s on and then you’d actually refer to the directory so you could see you know okay so there’s chicken casserole on, find chicken casserole in the directory and then you could see. Rather than it just being the recipe for that day because we felt again that was completely impractical. Somebody was gonna actually have to change it every day or every morning and afternoon or whatever… and catering staff could forget. So best to have directory which includes all menu items, not just one meal or todays menu items.* [Quote from indirect stakeholder] | 2 |
| Design Quality & Packaging (-) AND Consumer Needs & Resources (IS) (-) > Adapting the Interventions (+) | *I think there should be a different format and I don’t know what it is. But like there should be. Because going back to peanuts again and to dried fruit like these are items that are good for you on an overall basis. They’re good for your gut wellness. They’re good for you in a lot of different ways and they’re giving you different vitamins and that’s been not taken into account at all. It’s all about calorie… I think more people would buy into it then… Because instead of it being a number it’s something that relates back to them. Because like you know here do people know unless the poster is in front of them how many calories they should eat every day. But really I think we should be going on the fruit and veg, how many portions of fruit and veg do you have every day.* [Quote from direct stakeholder – hospital 1]  *You know picking calories up without anything really doesn’t mean an awful lot to people you know unless you can kind of put the evidence beside it or the consequences of exceeding your calorie intake or exceeding whatever you’re getting.* [Quote from indirect stakeholder – hospital 2]  *I think we’ve displayed the calories in a place that is suited to the catering staff to be put but actually I don’t think it’s prominent enough. When you’re stood looking at the counter the calories are behind you. I wanted them to be over the counters. I’d also like there to be when you’ve got your menu up for the day that the calories are written beside that menu for the day on a board.* [Quote from indirect stakeholder – hospital 3]  *I think there’s more of a need for its not just calorie posting because again you have to put it into context. What’s the point in telling somebody there’s X number of calories in something when they can’t put that into context? You know like well okay I know there’s 300 calories in that bun but what does that mean? How many calories am I allowed a day? And then that leads to other questions. So I think there needs to be a nutrition education program for the staff and to put things into context and saying this is what this is. The foods need everything rather than putting just emphases on calories. That’s not gonna give you an awful lot of information unless you know an awful lot about nutrition in the first place.* [Quote from indirect stakeholder – hospital 4] | 1, 2, 3, 4 |
| Design Quality & Packaging (-) > Adapting the Interventions (+) > Engaging: Internal Key Stakeholders (+) AND Engaging: Consumers (+) | *And I think there is a sense of either people reading the policy the way they want to or maybe it’s the way the policy is written but it’s just quite loose. You can display it any way you want, no clear information… I’ve seen it displayed in twenty different offices in twenty different ways…* *And I suppose I would love for it to look the same everywhere. You know if we’re the HSE and we’ve all these sites and we’re all meant to be calorie posting but it looks completely different. Some people have it on folders that you have to go and find it. Other people have it on chalk blackboards in the canteen. Other people have it on nice fancy menu boards. But I think it needs to look the same to be taken seriously that this is what’s in our food and what we’re eating…convincing the customer as well as maybe convincing the catering staff of the importance of it. And that would support the implementation so much…* [Quote from indirect stakeholder] | 2 |

**Symbols: > = leading to, (-) = barrier, (+) = facilitator**

**Abbreviations: IS = inner setting, OS = outer setting**
