# Supplementary material for: Factors Influencing Fidelity to a Calorie Posting Policy in Public Hospitals: A Mixed Methods Study
Source: Front Public Health. 2021 Aug 13;9:707668. doi: 10.3389/fpubh.2021.707668 (PMC8414889; doi:10.3389/fpubh.2021.707668)
Supplement: Supplementary file 8 [file Table_8.DOCX]

| **Additional file 8. Factors influencing fidelity** | | |
| --- | --- | --- |
| **Relationships** | **Sample Quotes** | **Hospital** |
| External Policy & Incentives (+) > Adherence to policy* | *Yeah I think audits by an external body would definitely help. It would work that it will drive internally the standards to go up… people would be more compliant.* [Quote from direct stakeholder] | 2 |
| External Policy & Incentives (-) > Lack of adherence to policy* | *So if it was an external person coming in to monitor… which I think has happened in the past it hasn’t gone down or been received very well. So you know… and they’re not there long-term and therefore you know they may try and comply while the person is there observing them but that’s not going to be a longstanding thing.* [Quote from indirect stakeholder] | 3 |
| Culture (OS) (-) > Engaging: Internal Key Stakeholders (-) > Compatibility (-) > Lack of adherence to standardised recipes > Inaccurate calorie information | *We weren’t being precise enough about the weighing of ingredients and the accuracy around the recipes…Because in the front of house we were allowing the scope for individual crafts to be demonstrated. So my skills and what I was good at and the recipes that worked for me may not necessarily have worked for somebody else so there was a lot of variation. But then with that variation there wasn’t the consistency. But we hadn’t quite grappled initially what calorie posting really actually meant so we were still carrying on with the variations. So while we were doing it, it might be based on Chef A but Chef B C and D had different. And that’s a cultural thing.* [Quote from direct stakeholder] | 4 |
| Culture (IS) (+) > Leadership Support (+) AND Engaging: Formally Appointed Internal Implementation Leaders (+) AND Engaging: Internal Key Stakeholders (+) AND Tension for Change (+) > Adherence to policy* | *And we always want to if there’s something that needs to be done or needs to be improved… It would be I suppose a quality improvement in terms of information that we’re providing, and it also makes us think more I suppose precisely and concisely around what we’re doing and why we’re doing it and the need for consistency and accuracy in what we’re doing.* [Quote from direct stakeholder] | 4 |
| Culture (IS) (-) > Reflecting & Evaluating (-) > Lack of adherence to policy* | *I suppose I’ve been working in hospitals for 24 years and this particular site is very much project orientated. So if a project is due to have a deadline or a date the focus then is on that project. But once that date passes then the focus then is put on to something else. So this week it’s calories. Next week it could be hydration. The following week it could be waste management. There’s no oversight over the course of the year. It’s very much project focused… So instead of I suppose having calories on every single week its only project focused. It might be on because you came this week the focus is on calories. But then you’re gone now so the next project focused on calories could be when your report is due out.* [Quote from direct stakeholder – hospital 2]  *I think they take on these projects and they look and sound good… They’re ticking boxes and that you know… But it’s a tick the box. It looks done. But is it really done? No one really checks… I don’t think we have fully implemented calorie posting yet...* [Quote from direct stakeholder – hospital 4] | 2, 4 |
| Consumer Needs & Resources (IS) (-) > Lack of adherence to policy* | *… like you know you’re doing all this work and then it’s just being ignored or you know there’s no uptake on it… The regulation states that we have to do it so we’ll continue to have to do it… You wouldn’t be as diligent about it.* [Quote from direct stakeholder – hospital 1] | 1 |
| Consumer Needs & Resources (IS) (-) > Lack of adherence to standardised portions > Inaccurate calorie information | *You might give a little bit more for males than female’s maybe… Probably friends I suppose as well. Associates or friends or… Yeah you’d know that you like a certain amount of pasta and she loves the pasta or she doesn’t like this and her likes and her dislikes.* [Quote from direct stakeholder – hospital 1]  *If I’m a very large man they go oh sure listen he probably needs more and they’re giving more.* [Quote from indirect stakeholder – hospital 2]  *Standardised serving utensils – ladles 2oz, 3oz or 4oz – different colour handles. As per serving staff, you can have standardised utensils but may give more or less. Noted during observation that some ladles were filled above rim or one and half ladles instead of one – generous serving for some customers (without the customer asking for same). Serving staff seemed familiar with their customers’ needs/preferences.* [Observation notes – hospital 3]  *I think the difficulty has been with portion sizes in terms of additional meals you know are the calories absolutely correct… There’s a bit of very good kind-heartedness you know. If you know them they give you a little bit more… I suppose it’s almost like inter-person reliability. You know some people just are known for giving bigger portions than others…* [Quote from indirect stakeholder – hospital 4] | 1, 2, 3, 4 |
| Consumer Needs & Resources (IS) (-) > Engaging: Consumers (-) > Lack of adherence to standardised portions > Inaccurate calorie information | *Calorie posting especially in this hospital and maybe it’s to do with portion control. Like you know if you have a portion, a portion is a portion is a portion, but it depends who’s giving the portion… So like there is a standard portion but like when somebody asks for more they receive more…* [Quote from direct stakeholder – hospital 1]  *You know or they’ll say can you give me some more, I want some more so then you give them some more you know.* [Quote from direct stakeholder – hospital 1]  *So there’s no standardisation in terms of quantity… we tried to do it at one stage and we met a lot of resistance from staff using the canteen. They don’t want standardised portions… they want to load their plate.* [Quote from direct stakeholder – hospital 2]  *But I think maybe other people might feel a little bit the other way that why are you giving me less today than you were giving me the other day and I don’t want it to be enforced.* [Quote from indirect stakeholder – hospital 3]  *Well what I would say to you in answer to that is they would all know what a portion is. But a lot of the time what happens is people will say will you give me a bit more, can I have more. A lot of the time. You’d never have anyone asking for less. So like there is a standard portion but like when somebody asks for more they receive more so the calories are skewed anyway.* [Quote from direct stakeholder – hospital 4] | 1, 2, 3, 4 |
| Compatibility (+) > Accurate calorie information | *I think the portion control sizes that you know are bought in packets or you know that are in measured, I don’t know the best word to say, you know measured items are easier obviously and are very accurate.* [Quote from indirect stakeholder] | 4 |
| Compatibility (-) > Leadership Support (-) AND Formally Appointed Internal Implementation Leaders (-) AND Engaging: Internal Key Stakeholders (-) > Lack of adherence to policy condition 1 (i.e. calorie posting is not in place for all food and drink items on sale) | *You know we live in the real world. In the real world like we have to flip menus over in the dining-room because it’s the same area that’s serving breakfast, mid-morning and lunch, so you have a different set of menus or a different set of offerings at different times of the day. So yes you’re relying on the guys who are on the ground, the guys who have the responsibility. That’s where you’re relying on people like the food and beverage manager or the dining supervisor and the girls on the ground to be doing the right thing at the right time. And you know occasionally I go down and the porridge is still up and the soup hasn’t been displayed. These are they’re kind of human errors and you can’t…* [Quote from direct stakeholder] | 4 |
| Complexity (-) > Inaccurate calorie information | *It’s different if it’s a portion of carvery of sliced beef or a piece of chicken or a piece of fish you know. That’s pure chicken or pure meat you’re getting. But when you have a saucy dish…So two ladles of chicken curry could contain different quantities of chicken or veg or whatever, more sauce or less sauce or stuff like that…So I don’t think you will ever get it accurate you know.* [Quote from direct stakeholder – hospital 1]  *So two ladles of chicken curry could contain different quantities of chicken or veg or whatever, more sauce or less sauce or stuff like that. So we are not getting an accurate calorie count there…* [Quote from direct stakeholder – hospital 4] | 1, 4 |
| Tension for Change (-) AND Relative Priority (-) > Leadership Support (-) AND Engaging: Formally Appointed Internal Implementation Leaders (-) > Lack of adherence to standardised recipes > Inaccurate calorie information | *I don’t really see the need to follow standardised recipes… Once we can upload it on to Nutritics and advertise the calories there shouldn’t be a need to take away the free hand from that. I’d be more concerned about the allergen information than the calorie information to be honest.* [Quote from direct stakeholder] | 2 |
| Structural Characteristics (-) > Leadership Support (-) AND Engaging: Formally Appointed Internal Implementation Leaders (-) > Lack of adherence to policy* | *There was only one catering manager in there with no assistant trying to run a whole big service, catering service… without an assistant catering manager implementation wasn’t done all the time.* [Quote from indirect stakeholder – hospital 1]  *There is no assistant catering manager here... role vacant… it would be there role to lead out on calorie posting… that’s why we don’t have calorie posting fully implemented here.* [Quote from direct stakeholder – hospital 2] | 2 |
| Structural Characteristics (-) > Engaging: Internal Key Stakeholders (-) > Lack of adherence to policy condition 1 (i.e. calorie posting is not in place for all food and drink items on sale) | *But then we went through a phase where we didn’t have the staff so then we had to have the calories and allergens for limited part of menu.* [Quote from direct stakeholder] | 4 |
| Structural Characteristics (-) > Available Resources (-) > Engaging: Internal Key Stakeholders (-) > Inaccurate calorie information | *And there are certain levels of inconsistency which make me wonder about accuracy. Sometimes it’s to do with resources where you’re a couple of chefs down.* [Quote from direct stakeholder] | 4 |
| Structural Characteristics (-) > Available Resources (-) AND Culture (IS) (-) > Relative Priority (-) > Engaging: Internal Key Stakeholders (-) > Lack of adherence to policy* | *But as a result then and they have gone through you know loads of periods when they’ve been really short staffed. They will prioritise getting everything 100% for the patients but the staff canteen suffers as a result. Hence calories not fully done… So patients come first. If you don’t resource your catering department properly your staff canteen will suffer.* [Quote from indirect stakeholder – hospital 1]  *There was a lack of resources and there is a lack of resources in the canteen. Those resources were always being placed first and foremost on the patient food. So this meant calorie posting wasn’t implemented.* [Quote from indirect stakeholder – hospital 2] | 1, 2 |
| Available Resources (+) > Engaging: Internal Key Stakeholders (+) > Adherence to policy* | *But a factor in terms of being able to implement and deliver you know on the desired outcomes is you have to resource the departments to deliver.* [Quote from indirect stakeholder] | 1 |
| Available Resources (-) > Engaging: Internal Key Stakeholders (-) > Engaging: External Change Agents (-) > Inaccurate calorie information | *And you know I wasn’t able to give him time to even make sure they were doing things right. Now I did crosscheck some of the analysis they came up with. And some were very wrong. And I put them on the right track. And said look you have to be very careful when you see something that’s not… You know porridge made from water is coming up at like 300 calories per portion. You need to go back and check something like that. So there was a lot of in the beginning say looking back and checking that. And it was coming to me initially. And I said look I can’t’ keep doing this, I don’t have the time… we should have been able to sit down with them and be crosschecking the students and doing checks to make sure everything was working out.* [Quote from indirect stakeholder]  *I noticed on the posters like some calories straight off as a dietitian I could tell that’s not right, that’s wrong… the students who were working on the project didn’t have the knowledge… and because there was no direct supervision from a dietitian I think there are probably a lot of errors in the calculations. We just don’t have the time to supervise them.* [Quote from indirect stakeholder] | 4 |
| Access to Knowledge & Information (-) > Lack of adherence to standardised recipes > Inaccurate calorie information | *Reason for compliance issues with standardised recipes… I think it definitely comes down to lack of training, not resistance from the catering people to be honest with you.* [Quote from indirect stakeholder] | 2 |
| Access to Knowledge & Information (-) > Lack of adherence to standardised portions > Inaccurate calorie information | *Not giving correct portions is more to do with lack of training, than resistance from the catering people.* [Quote from indirect stakeholder – hospital 2]  *You know there’s a lack of consistency in their management of portions because there’s no training for the staff.* [Quote from indirect stakeholder – hospital 4] | 2, 4 |
| Access to Knowledge & Information (-) > Inaccurate calorie information | *… sometimes I think you know to be quite honest I’d google black pudding to find out what the calories are in it but it mightn’t be the black pudding that I’m using. And like the recipe could be totally different and sure there could be a lot of other things… so you could be posting in inaccurate calorie count.* [Quote from direct stakeholder] | 1 |
| Leadership Support (-) AND Engaging: Formally Appointed Internal Implementation Leaders (-) > Lack of adherence to standardised recipes > Inaccurate calorie information | *They were going to do out some standardised recipes. They could possibly have them done somewhere. But then we were told we could do what we want.* [Quote from direct stakeholder] | 2 |
| Leadership Support (+) AND Engaging: Formally Appointed Internal Implementation Leaders (+) AND Reflecting and Evaluating (+) > Adherence to standardised portions | *What helps is [catering manager name] standing beside you saying that’s extra, so we will follow correct portions.* [Quote from direct stakeholder] | 1 |
| Leadership Support (+) AND Formally Appointed Internal Implementation Leaders (+) AND Reflecting and Evaluating (+) > Engaging: External Change Agents (+) > Accurate calorie information | *[Assistant catering manager] would have worked very closely with them on that side of it and I would have. I’d be coming in and kind of just you know you’d look at it and then you’d say okay let’s pick a few and talk us through. Just to get the accuracy right. Yeah.* [Quote from direct stakeholder] | 4 |
| Engaging: Internal Key Stakeholders (-) > Inaccurate calorie information | *I noticed the calorie counts were wrong… unfortunately the caterers wouldn’t have been able to have the knowledge to say that was right or wrong.* [Quote from indirect stakeholder] | 4 |
| Engaging: Internal Key Stakeholders (-) > Engaging: Internal Key Stakeholders (-) > Lack of adherence to policy* | *Not that the canteen management themselves didn’t want our help, but when we pulled back or stopped helping it wasn’t maintained in any way.* [Quote from indirect stakeholder]  *And looking back at it now it was probably the worst thing to do because we made a big fanfare, it was expected to be spoon fed to them and then in a month or two months we weren’t there anymore. It completely stopped.* [Quote from indirect stakeholder] | 2 |
| Engaging: External Key Stakeholders (-) > Lack of adherence to standardised recipes > Inaccurate calorie information | *So all of the recipes have been standardised but again as you know yourself again sometimes ingredients are in short supply from the suppliers and I can’t say definitively that one item is not going to be swapped in for another and therefore you know the calorie count might not reflect these changes.* [Quote from indirect stakeholder] | 2 |
| Engaging: External Key Stakeholders (-) AND Reflecting & Evaluating (-) > Lack of adherence to standardised recipes > Inaccurate calorie information | *I was out here when she questioned me on the mayonnaise, the low fat. That was another thing. We went all low fat mayonnaise for coleslaw and potato salad. And then that one day she was in she went down to the fridges and there was no low fat mayonnaise in the kitchen. It was full fat… And you see what I mean. If you don’t spot-check. It was supposed to be low fat and we had been using full fat by error… Something happened that the wrong one was delivered. [Catering supervisor name] wouldn’t have known that and no chef said it to her. And it was just automatically used and not said to us, whereas we could have changed the signage.* [Quote from direct stakeholder] | 4 |
| Engaging: External Change Agents (+) > Accurate calorie information | *Yeah we have a student coming here… They’re a dietetic student and we’re going to be using their knowledge and the rest of it… Only the fact that I’m getting these students means that I can do some work on it and it’ll be accurate.* [Quote from direct stakeholder] | 1 |
| Engaging: External Change Agents (+) > Adherence to policy condition 1 (i.e. calorie posting is in place for all food and drink items on sale) | *Without the student we wouldn't be this far at all… You might have a small section of the menu calorie posted but you wouldn't have it all done.* [Quote from direct stakeholder] | 3 |
| Engaging: External Change Agent (-) AND External Policy & Incentives (-) > Lack of adherence to policy* | *The happy heart award I think is a really good initiative but again I think it’s something that you know it shouldn’t be just reported we met this criteria in our hospital that you set out, we’ve done it so we got the award. It should be something possibly that’s double checked or every so often spot checked. If there’s no enforcement of it that award means nothing you know… standards will start to slip.* [Quote from indirect stakeholder] | 3 |
| Engaging: Consumers (+) > Reflecting & Evaluating (+) > Accurate calorie information | *The other day we had a staff member coming in and they were having their lunch and they were looking at the menu and they said how come the healthy option has more calories than another thing. But like that was an error on my behalf that I had the wrong thing or we had posted the wrong thing. But it just shows you that there is an awareness and that somebody was looking at it and feeding back to us the fact that it wasn’t as it should be or whatever. So we corrected it.* [Quote from direct stakeholder] | 1 |
| Design Quality & Packaging (-) > Lack of adherence to policy condition 1 (i.e. calorie posting is not in place for all food and drink items on sale) | *If you look at the butters now there’s little slips beside them or some of them maybe have fallen out. Even the triangle of cheese. Everything was calorie counted… But there should be little portion things beside them to show that they are… Some of them got lost. Some are up in the press. They are there*. [Quote from direct stakeholder – hospital 1]  *Now there’s still a lot of calories that’s not on the big chart. It’s difficult to keep it updated with new menu items as need to re-do poster each time.* [Quote from direct stakeholder – hospital 4] | 1, 4 |
| Design Quality & Packaging (-) > Lack of adherence to policy* | *We display calorie information for the nutritional hydration week each year but it’s taken down after because really it just is a bit busy if you know what I mean. Maybe that’s the point of where they’re doing the choice but you know if you’ve a busy counter top and you have this in front of you you know it isn’t the ideal. It really isn’t. So it’s not as though it’s something that we could kind of just leave it up because it’s not the ideal way to do it.* [Quote from indirect stakeholder] | 2 |
| Design Quality & Packaging (-) > Access to Knowledge & Information (-) > Engaging: External Change Agents (-) > Lack of adherence to policy condition 3 (i.e. calorie information is not always displayed per standard portion or per meal) | *Just even recommendations or what you have to actually have in place for the calorie posting. It’s like we knew we had to have the nutritional analysis done and we were getting the energy content and the calories up for that. So therefore you know we just put them up. Whether we put them up per ounce or per portion I think it was just down to the student kind of feeling this was the most appropriate way to put it out there... often it was a mix of per ounce and per portion on the display board.* [Quote from indirect stakeholder] | 3 |

**Symbols: > = leading to, (-) = barrier, (+) = facilitator, * = policy condition(s) not specified**

**Abbreviations: IS = inner setting, OS = outer setting**
